# The effect of combination prevention strategies on HIV incidence among gay and bisexual men who have sex with men in the UK: a model-based analysis

**DOI:** 10.1016/S2352-3018(23)00204-7

**Published:** 2023-11-01

**Authors:** Valentina Cambiano, Alec Miners, Fiona C Lampe, Sheena McCormack, O Noel Gill, Graham Hart, Kevin A Fenton, Gus Cairns, Marc Thompson, Valerie Delpech, Alison J Rodger, Andrew N Phillips

**Affiliations:** Institute for Global Health, University College London, London, UK; Department of Health Services Research and Policy, London School of Hygiene & Tropical Medicine, London, UK; Institute for Global Health, University College London, London, UK; MRC Clinical Trials Unit at University College London, London, UK; UK Health Security Agency, London, UK; Institute for Global Health, University College London, London, UK; Office for Health Improvement and Disparities (London) and NHS London, London, UK; NAM Publications, London, UK; The Love Tank CIC, London, UK; UK Health Security Agency, London, UK; Institute for Global Health, University College London, London, UK; Institute for Global Health, University College London, London, UK

## Abstract

**Background:**

In the UK, the number of new HIV diagnoses among gay and bisexual men who have sex with men (GBMSM) has decreased substantially. We aimed to understand the contribution of different interventions in reducing HIV incidence so far; to estimate future HIV incidence with continuation of current policies and with further scaling up of current interventions; and to estimate the maximum additional annual cost that should be spent towards these interventions for them to offer value for money.

**Methods:**

We calibrated a dynamic, individual-based, stochastic simulation model, the HIV Synthesis Model, to multiple sources of data on HIV among GBMSM aged 15 years or older in the UK. Primarily these were routine HIV surveillance data collected by the UK Health Security Agency. We compared HIV incidence in 2022 with the counterfactual incidence: if HIV testing rates stopped increasing in 2012 and the policy of antiretroviral therapy (ART) at diagnosis was not introduced in mid-2015; if pre-exposure prophylaxis (PrEP) was not introduced; if condom use was low from 2012 in all GBMSM, at levels similar to those observed in 1980; and in the first and second scenario combined. We also projected future outcomes under the assumption of continuation of current policies and considering increases in PrEP and HIV testing uptake and a decrease in condomless sex.

**Findings:**

Our model estimated a 77% (90% uncertainty interval [UI] 61–88) decline in HIV incidence since around 2014, with an estimated 597 infections ([90% UI 312–956]; 1·1 per 1000 person-years [90% UI 0·6–1·8]) in men aged 15–64 years in 2022. Both PrEP introduction and increased HIV testing with ART initiation at diagnosis each had a substantial effect on HIV incidence. Without PrEP introduction, we estimate there would have been 2·16 times the number of infections that actually occurred (90% UI 1·06–3·75) between 2012 and 2022; without increased HIV testing and ART initiation at diagnosis there would have been 2·18 times the number of infections that actually occurred (1·18–3·60), and if condomless sex was at the levels before the HIV epidemic, there would have been 2·27 times the number of infections that actually occurred (0·9–5·4). If rates of testing, ART use, and PrEP use remain as they are currently, there is a predicted decline in incidence to 388 HIV infections in 2025 (90% UI 226–650) and to 263 (137–433) in 2030. Increases in HIV testing and PrEP use were predicted to accelerate the decline in HIV incidence. Given the quality-adjusted life-year (QALY) benefit and a cost-effectiveness threshold of *£*30 000 per QALY gained, in order to be cost-effective an additional *£*1·62 million could be spent per year to increase testing levels by 34% (90% UI 25–46) and PrEP use by 55% (10–107). To achieve that, a 16% reduction in the cost of delivery of testing and PrEP would be required.

**Interpretation:**

Combination prevention, including a PrEP strategy, played a major role in the reduction in HIV incidence observed so far in the UK among GBMSM. Continuation of current activities should lead to a continued decline; however, it is unlikely to lead to reaching the target of fewer than 50 HIV infections per year among GBMSM by 2030. It will be important to reduce costs for testing and PrEP for their continued expansion to be cost-effective.

**Funding:**

National Institute for Health Research under its Programme Grants for Applied Research Programme and Medical Research Council—UK Research and Innovation.

## Introduction

In Europe and many other high-income settings, including the UK, sex between men is the primary mode of HIV transmission.^1,2^ In recent years, a decline in new HIV diagnoses among gay and bisexual men who have sex with men (GBMSM) has been observed in western Europe,^2^ in most cases in the context of an increase in HIV testing.^3^ In the UK, the number of new HIV diagnoses among GBMSM decreased by two thirds from a peak of 3214 in 2014^4^ (2977 in England) to 979 in 2021 in England, although a very small increase was observed from 2020 to 2021 among GBMSM first diagnosed in England.^1^

Among GBMSM, after a reduction in condomless sex in the 1980s,^5,6^ a resurgence was reported after the introduction of antiretroviral therapy (ART) in the mid-1990s^7,8^ and an increase has been observed since then.^9^ In the UK, the offer of free sexual health consultations and condom provision in sexual health clinics and in the community has been available since the mid-1980s. However, it is in the past decade that, over time, a combination of additional policies has been implemented. Frequent repeat HIV testing has been offered for men with higher risk sexual behaviours and in various health-care settings (sexual health services, special sexual health services, general practices, and hospitals) and in the community by using newer technologies, such as HIV self-sampling and HIV self-testing.^1^ ART at diagnosis has been offered for people living with HIV^10,11^ and pre-exposure prophylaxis (PrEP) has been offered to people without HIV to prevent its acquisition,^12,13^ with particular effort in targeting those at highest risk. All these interventions are now offered free of charge and confidentially in the UK. Regarding PrEP in particular, in England it was offered to a capped number (26 000) of people until mid-2020 through the PrEP Impact trial^14^ and is now freely available through the National Health Service (NHS), although barriers to accessing it have been reported.^15^ PrEP has also been available through self-sourcing on the internet since 2015.

Despite these efforts, new HIV transmissions are still occurring, estimated at 620 (95% credible interval 390–910) among GBMSM in England in 2021.^16^ The UK Government has committed to end HIV transmissions in England by 2030 (for GBMSM in England this is defined as <50 transmissions).^17^ The HIV commission has suggested a new interim milestone of an 80% reduction in HIV infections by 2025 compared with 2019, equating to fewer than 250 new HIV infections among GBMSM in England. The commission also identified widespread HIV testing, made available routinely across the NHS and delivered as opt-out in emergency departments, as the single most important intervention to meet the 2025 and 2030 goals. In December, 2021, the UK Government announced it would fund opt-out HIV testing largely in emergency departments in all areas of very high HIV prevalence, with a *£*20 million financial package. The Government also launched a new HIV Action Plan^18^ with four main objectives: (1) to ensure equitable access and uptake of HIV prevention programmes; (2) to scale up HIV testing in line with national guidelines (including postal or collection HIV self-sampling services); (3) to optimise rapid access to treatment and retention in care; and (4) to improve quality of life for people with HIV and address stigma.

We have previously used the HIV Synthesis Model for GBMSM to investigate what was required to reduce HIV incidence in GBMSM to below 1 per 1000 person-years (ie, <535 new infections per year) by 2030, and whether that was likely to be cost-effective.^19^ We found that to achieve that target, which is less ambitious than the current 2030 goal, the proportion of GBMSM living with HIV with viral suppression needed to increase from less than 60% (as it was estimated to be in 2015) to 90%, assuming no increase in condomless sex. For 90% of all GBMSM living with HIV to be virally suppressed, substantial increases in HIV testing needed to occur, such that more than 90% of GBMSM are diagnosed within a year of infection, and ART needed to be initiated at diagnosis. We also used the same model to evaluate the impact and cost-effectiveness of the introduction in 2016 of a PrEP programme with sexual-event-based use of emtricitabine plus tenofovir for GBMSM in the UK.^20^ We found that the introduction of a PrEP programme that would be interrupted when the overall HIV incidence dropped below 1 per 1000 person-years would be cost-saving over 80 years, but that these findings were particularly sensitive to the cost of antiretrovirals for treatment and PrEP and to the underlying trend in condomless sex.

We aimed to understand the contribution of the components of combination prevention to the decrease in HIV incidence observed among GBMSM in the UK and to assess whether the UK is on course to reach the target set out by the HIV commission. We also aimed to estimate the impact of further scaling up of current interventions and to estimate the maximum additional annual cost that should be spent towards these activities for them to offer value for money.

## Methods

### Study design and model

We used a dynamic, individual-based, stochastic simulation model (the HIV Synthesis Model) that recreates the lifetime HIV risks and, for those acquiring HIV, HIV progression and treatment outcomes, of the GBMSM population in the UK. We simulated a fraction (4·5%) of the UK GBMSM population. The model and the calibration approach have been described in detail elsewhere,^19–21^ and have now been updated to include observed data to the end of 2021 (see appendix p 3 for a brief description of the model, appendix pp 12–20 for details on the calibration, and appendix p 21 onwards for full model details).

In brief, we modelled in all GBMSM aged 15 years or older: age (this is the only variable modelled from age 0 years), condomless sex with primary (long-term) and short-term (eg, casual) partners, presence of other sexually transmitted infections, and HIV testing patterns and diagnosis. In GBMSM living with HIV aged 15 years or older, we modelled viral load, CD4 cell count, use of specific antiretroviral drugs, adherence to ART, presence of specific resistance mutations, risk of AIDS, and death, including death from non-AIDS conditions. We accounted for the possibility that GBMSM living in the UK might have sex with GBMSM living with HIV who are non-resident in the UK and that these individuals might be less likely to be virally suppressed. The parameter values determining population growth, sexual behaviour (including whether this is with partners not resident in the UK), the transmission rate (including of virus with resistance mutations), testing patterns, the extent to which HIV diagnosis leads to a reduction in condomless sex, PrEP uptake, and interpatient variation in rate of CD4 cell count increase were varied with each model simulation run by sampling from distributions. The resulting outputs from the model were compared with various data sources (appendix pp 17–19). We ran the model more than 300 000 times up to the end of 2022 and selected the 302 simulations able to reproduce an epidemic close to that observed in reality.

### Analyses

We conducted two main analyses. The first investigated the effect of specific interventions on the decline in HIV incidence in the period between 2012 and 2022, referred to as the counterfactual scenario analysis. The second analysis assessed whether the targets will be achieved if interventions continue to be delivered at the current rate, and estimated the maximum additional annual cost that should be spent towards these activities, in order for them to offer value for money, referred to as the cost-effectiveness evaluation.

In the counterfactual scenario analysis, having reconstructed the epidemic to date, we compared the HIV incidence in 2022 with that in counterfactual scenarios in which interventions from 2012 were either not introduced or were introduced to a lower extent, to understand the role of the different interventions.

For the cost-effectiveness evaluation analysis, we projected forward from 2023 to 2103, with each run starting from a randomly selected one of the 302 simulations with a good fit to observed data. In total, 1000 simulations were run going forward. We considered the benefit over the first 20 years and over the full 80-year timeframe.

#### Counterfactual scenario analysis (2012–22)

We compared a reference scenario to four counterfactual scenarios. The reference scenario, in which we aimed to mimic the epidemic as it has progressed in reality, assumes that: condomless sex, regardless of HIV status, has been increasing over time (linearly since 1998; appendix pp 4, 14); that the rate of HIV testing, defined as the probability of testing per 3 months in an individual not previously diagnosed with HIV, has been increasing (since 2006; appendix pp 4, 14); that a policy of ART at diagnosis was introduced in mid-2015, increasing the probability of starting ART per 3 months from 0·075 to 0·35, and that this doubled in mid-2018; and that PrEP was introduced in 2013 at a small scale through the PROUD study^22^ and then expanded. The extent to which condom use has been declining and the testing rate has been increasing was determined by the calibration process. Regarding PrEP, we assumed, similarly to the PROUD study,^22^ that GBMSM are eligible if: they had a negative HIV test at PrEP initiation; they had reported condomless anal intercourse in the previous 3 months (unless the only partner they had condomless sex with was a long-term partner virologically suppressed on ART); and they had had an additional documented negative HIV test in the preceding year. In addition, we assumed that men on PrEP test for HIV every 3 months, as recommended by the British Association for Sexual Health and HIV.^12^ In practice, one of the first routes to access PrEP was self-sourcing it.

The counterfactual scenarios considered for the period 2012–22 are as in the reference scenario except for: HIV testing rates stopped increasing in 2012 and the policy of ART at diagnosis was not introduced in mid-2015; PrEP was not introduced; condomless sex was high from 2012, at levels similar to those observed in 1980; HIV testing rates stopped increasing in 2012; and the policy of ART at diagnosis was not introduced in mid-2015 and PrEP not introduced. The implementation of these scenarios is shown in the appendix (p 6). The assumptions on the efficacy of the prevention interventions are summarised in the table.

#### Cost-effectiveness evaluation (2023–2103)

To assess the probability that the targets will be achieved if interventions continue to be delivered at the current rate, we calculated the proportion of simulations where the target is achieved. In the cost-effectiveness evaluation, the scenarios compared from 2023 onwards were: no change in interventions (ie, sexual behaviour, HIV testing behaviour, the probability of being on ART, and the probability of initiating and remaining on PrEP are fixed to the level reached in 2022); increase in the rate of HIV testing; increase in PrEP use; decrease in condomless sex; and increase in HIV testing and PrEP use. The HIV prevention interventions evaluated in this analysis were those that were identified through a systematic review to have substantive evidence of effectiveness through a randomised controlled trial design.^25^ These were interventions to reduce condomless anal intercourse, such as one-to-one counselling, group interventions (theory-driven, interactive, and behaviour change group sessions), and online interventions (using cognitive or behavioural interventions involving video games, interactive modules, short videos, or online chat); and PrEP. We assumed that the PrEP programme will be stopped and not re-initiated when the overall HIV incidence in the GBMSM population decreases below 1 per 10 000 person-years for 5 years. We also considered an increase in HIV testing, leading to immediate ART initiation in those diagnosed with HIV, that could be achieved, for example, through the distribution of HIV self-testing, as evaluated in the SELPHI study.^26^ This randomised controlled trial showed that the availability of free HIV self-testing significantly increased the rate of HIV testing, although this did not translate to an increase in HIV diagnoses.^26^

We calculated the maximum additional cost for HIV testing and PrEP in order for each scenario to be cost-effective as the difference between the product of the cost-effectiveness threshold (*£*30 000 per quality-adjusted life-year [QALY] gained) and the QALYs gained and the difference in costs other than HIV testing and PrEP. The unit costs assumed for HIV testing, prevention, and care are listed in appendix pp 8–11. In summary, the cost of HIV testing was assumed to be *£*32 per test; the cost of clinical care for people living with HIV included an assumed annual cost of ART of *£*2667 and an estimated annual cost of providing care ranging from £1174 to £6482;^27^ assumed annual cost of antiretrovirals for PrEP if used daily was *£*183^28^ and of monitoring people on PrEP was *£*194 in the first year and *£*104 afterwards; and the cost of a course of antiretrovirals for post-exposure prophylaxis was assumed to be *£*486^28,29^ with a cost of *£*248 for the visit to prescribe it. We did not include in the analysis the costs for the activities (such as one-to-one counselling, group interventions, and online interventions) enabling improvements in prevention and HIV testing uptake, or costs for the demand creation activities. The unit costs were assumed to remain at the current level for the entire time period, although all costs and health outcomes are discounted at an annual rate of 3·5%.^30^ A period of 80 years was used, given the National Institute for Health and Care Excellence recommendations considering a lifetime horizon.^30^

We used SAS (version 9.4) for our model program.

### Role of the funding source

The funders of the study had no role in study design, data collection, data analysis, data interpretation, or writing of the report.

## Results

**Figure 1:**
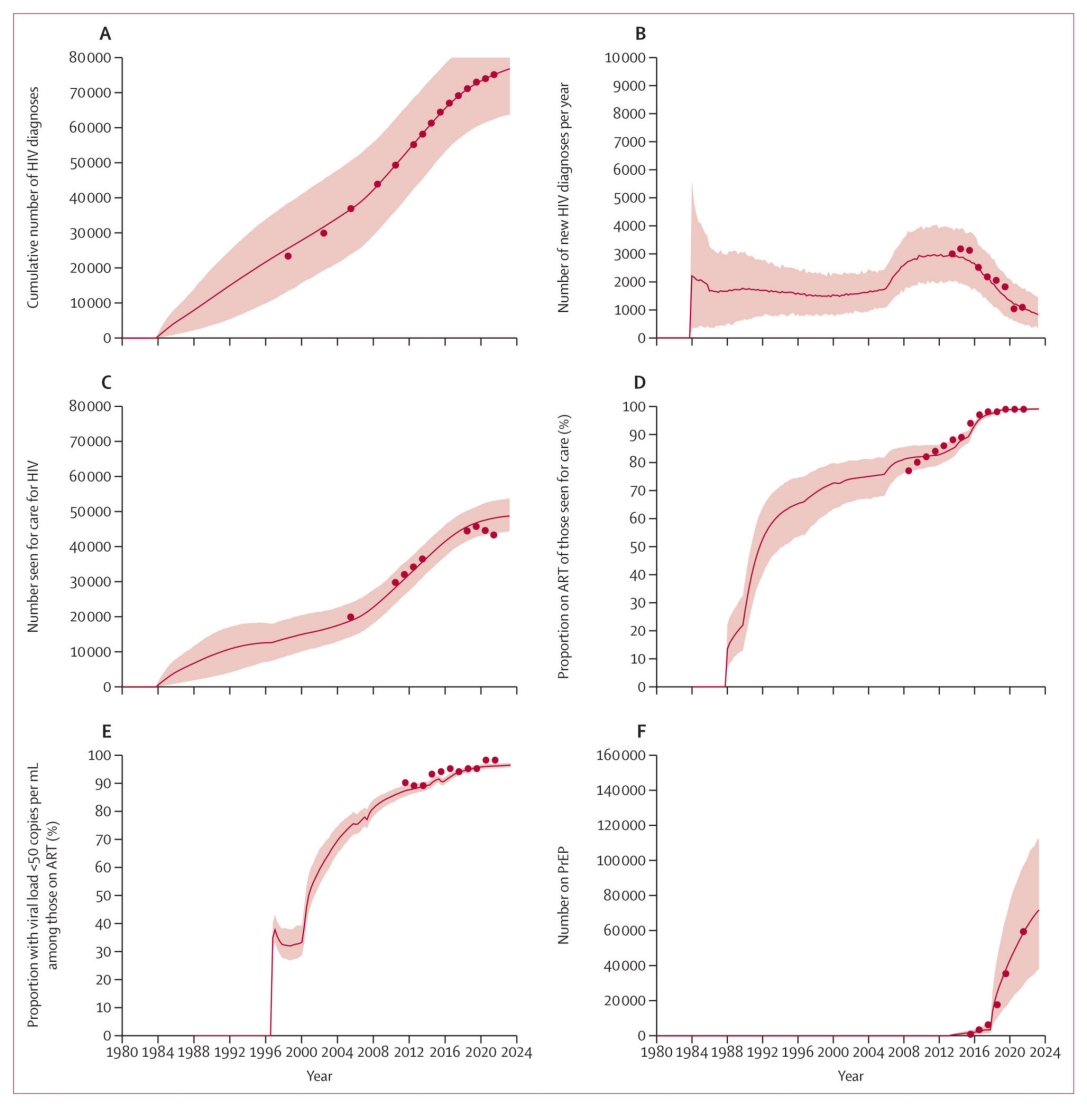
Comparison of model outputs among GBMSM in the UK, with observed data from the UK Health Security Agency Line is median over model runs, shading represents 90% uncertainty interval. (A) Cumulative number of HIV diagnoses. (B) Number of new HIV diagnoses per year for GBMSM aged 15–64 years. (C) Number of GBMSM aged 15 years and older seen for care for HIV. (D) Proportion on ART of those seen for care. (E) Proportion of those on ART with a viral load less than 50 copies per mL. (F) Number of GBMSM on PrEP. ART=antiretroviral therapy. GBMSM=gay and bisexual men who have sex with men. PrEP=pre-exposure prophylaxis.

**Figure 2:**
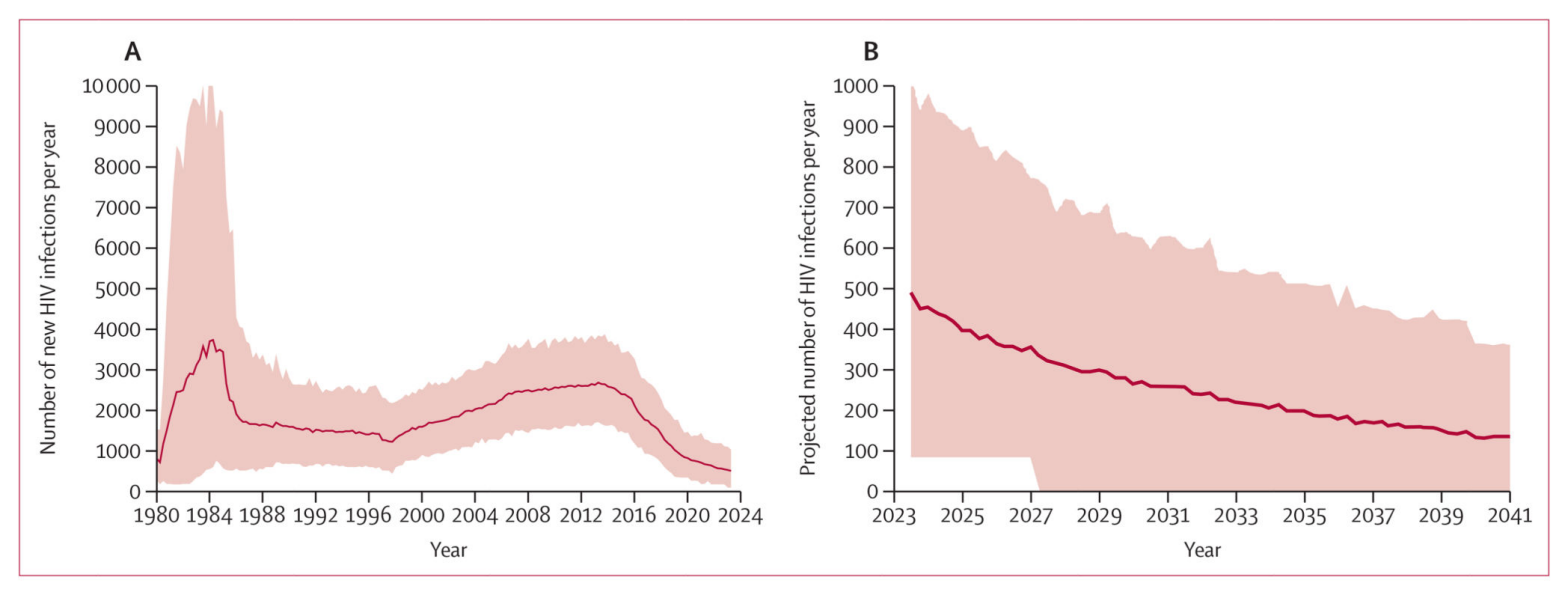
Estimated trends in HIV incidence among GBMSM in the UK Line is median over model runs, shading represents 90% uncertainty interval. (A) Inferred HIV incidence (number of new infections per year) from 1980 to 2022. (B) Projected future number of infections per year over 20 years from 2023 if levels of interventions remain as they were in 2022 (note difference in scale). GBMSM=gay and bisexual men who have sex with men.

The model was calibrated to observed data from the UK Health Security Agency (UKHSA) for each of the model outputs (figure 1; appendix pp 4–5). The number of GBMSM living with HIV in the UK estimated by our model (appendix p 5) suggested a 77% (90% uncertainty interval [UI] 61–88) decline in incidence since around 2014 (figure 2A). The inferred estimated HIV incidence among GBMSM aged 15–64 years was 597 infections (90% UI 312–956; figure 2A) in 2022, or 1·1 infections per 1000 person-years (90% UI 0·6–1·8).

**Figure 3:**
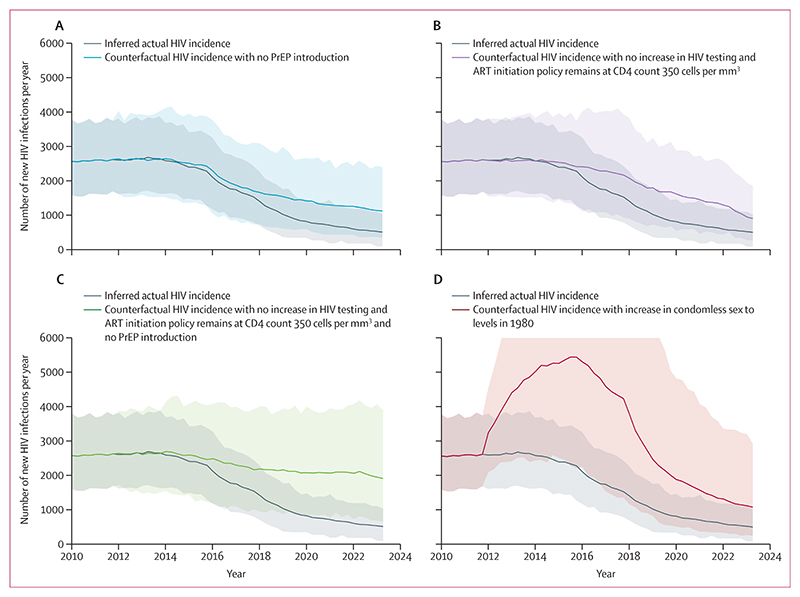
Inferred actual HIV incidence and HIV incidence for each counterfactual scenario since 2012 among GBMSM in the UK Line is median over model runs, shading represents 90% uncertainty interval. (A) Inferred actual HIV incidence and counterfactual HIV incidence with no PrEP introduction. (B) Inferred actual HIV incidence and counterfactual HIV incidence with no increase in HIV testing and ART initiation policy remains at CD4 count 350 cells per mm^3^. (C) Inferred actual HIV incidence and counterfactual HIV incidence with no increase in HIV testing and ART initiation policy remains at CD4 count 350 cells per mm^3^ and no PrEP introduction. (D) Inferred actual HIV incidence and counterfactual HIV incidence with increase in condomless sex to levels in 1980. ART=antiretroviral therapy. GBMSM=gay and bisexual men who have sex with men. PrEP=pre-exposure prophylaxis.

Results of our counterfactual-scenario analysis suggest that PrEP introduction, reduction in condomless sex (compared with the levels in 1980), and increased HIV testing with ART initiation at diagnosis each had a substantial effect on HIV incidence (figure 3). We estimate that in 2022, without PrEP introduction, there would have been a mean 1228 infections ([90% UI 577–2153], 2·16 times the number that actually occurred [90% UI 1·06–3·75]); without increased HIV testing with ART initiation at diagnosis, there would have been a mean 1237 infections ([588–2224], 2·18 times the number that actually occurred [1·18–3·60]), and without either intervention, there would have been a mean 2044 infections ([958–3941], 3·64 times the number that actually occurred [1·67–6·55]; figure 3). We also considered what the incidence would have been had condomless sex increased in 2012 to the level in 1980, before the HIV epidemic (appendix p 6). This would have led to a substantial rise in new infections from 2013, with some decrease by 2022 due to greater HIV testing, ART at diagnosis, and PrEP. However, in 2022 there would still have been a mean 1299 (466–3215) infections (2·27 times the number that actually occurred; 0·9–5·4).

**Figure 4:**
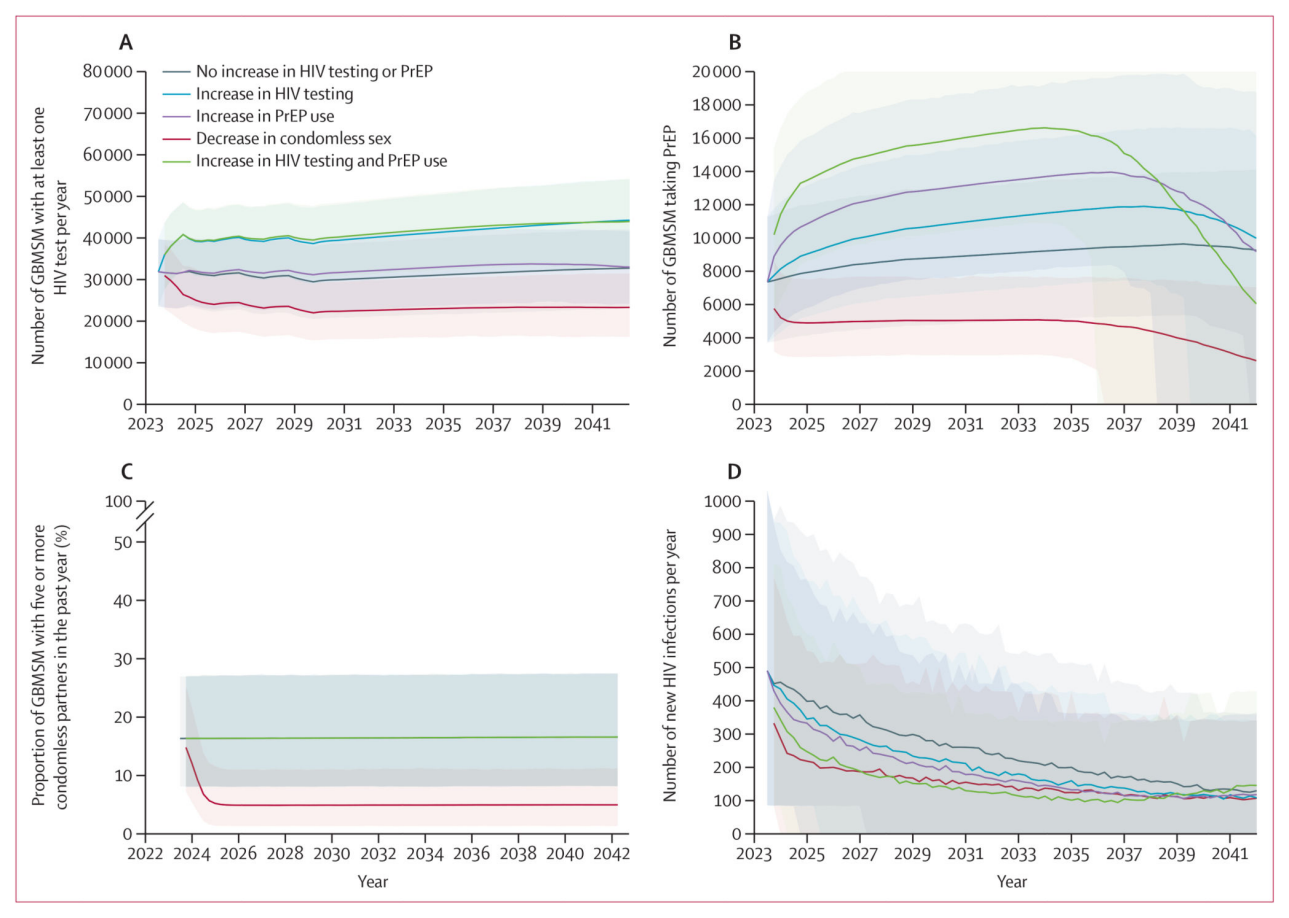
Projected outcomes over 20 years from 2023 according to whether testing and PrEP use are increased beyond current levels Line is median over model runs, shading represents 90% uncertainty interval. (A) Number of GBMSM with at least one HIV test per year. (B) Number of GBMSM taking PrEP. (C) Proportion of GBMSM with five or more condomless partners in the past year. (D) Number of new HIV infections per year. GBMSM=gay and bisexual men who have sex with men. PrEP=pre-exposure prophylaxis.

We estimated the predicted trend in HIV incidence over the coming 20 years if interventions continue to be provided at the current rate (figure 2B). The projection suggests a continued decline in HIV incidence in GBMSM, to 0·20 per 1000 person-years (90% UI 0·09–0·37), around 100 new infections per year in 2043, with a 23% chance of achieving the 2025 target of fewer than 250 new HIV infections among GBMSM in England and 0% chance of achieving the 2030 target of fewer than 50 new HIV infections among GBMSM in England. If rates of testing, ART use, and PrEP use remain as they are currently, the incidence is predicted to decline to 388 HIV infections in 2025 (90% UI 226–650) and to 263 (137–433) in 2030. Trends over this period in other model outputs are shown in figure 4 and the appendix (p 7).

We estimated the potential impact of four modifications from 2023: an increase in HIV testing levels (by 32% [90% UI 25 to 43], from 39% to 52% tested in the past year); an increase in PrEP use (by 39% [90% UI 13 to 59]); a decrease in condomless sex such that 9% of GBMSM (compared with 24% without the change) had at least one condomless sex partner in the past 3 months (and 5%, compared with 16% without the change, had at least five); and the increase in HIV testing (by 34% [25 to 46]) and PrEP use (by 55% [10 to 107]) combined (figure 4). Note that the steep decline in the number on PrEP is due to our assumption that the PrEP programme will be stopped and not re-initiated when the overall HIV incidence in the GBMSM population drops below 1 per 10 000 person-years for 5 years. Each of these four modifications is predicted to lead to a lower HIV incidence. The increase in testing is projected to lead to a mean change in HIV incidence over 20 years of –15% (90% UI –32 to 6). The increase in PrEP use is projected to lead to a mean change in HIV incidence of –23% (–6 to –39); testing and PrEP combined to a mean change of –34% (–15 to –52); and the decrease in condomless sex to a mean change of –36% (–19 to –54). Of note, the increase in testing and PrEP combined could lead to a 76% chance of achieving the target in 2025, but still only a 3% chance of achieving the target in 2030.

Considering an 80-year time horizon, the increase in testing and PrEP is projected to lead to a mean of 21 deaths averted per year, of an annual mean of 704 HIV deaths among GBMSM in the UK, and 89 infections averted per year, corresponding to 54 discounted QALYs gained per year. Assuming a cost-effectiveness threshold of *£*30 000 per QALY gained, this means up to an additional *£*1·62 million could be spent on HIV testing and PrEP per year in order to be cost-effective. This can be achieved if the cost of delivery of testing and PrEP is reduced by at least 16%.

## Discussion

Our results suggest that both PrEP introduction (with its consequent repeat HIV testing in people at risk of contracting HIV, as people on PrEP are assumed to be testing every 3 months), increased HIV testing with ART initiation at diagnosis, and condom use each had a substantial effect on HIV incidence among GBMSM in the UK. Without any one of them, the number of HIV infections in 2022 would have been roughly double, so the importance of scaling up such interventions in settings with similar epidemics is not in doubt. The exact number of GBMSM receiving PrEP in the UK either through the NHS or privately is unknown, but according to a convenience sampled survey (from the mailing list of iwantPrEPnow, gay dating apps, social media promotion, and community-based organisations), 32% in 2018 and 22% in 2020 of men who had ever used PrEP were buying it from the internet.^31^ In the reference scenario, we considered a relatively wide range for the number of people on PrEP. We had previously found that the greater the use of PrEP among eligible GBMSM, the greater the health benefit.^20^

Our results also suggest that HIV incidence is going to decline if the current level of interventions is maintained, with a 23% chance of reaching the target of fewer than 250 new HIV infections among GBMSM in England by 2025 and a 0% chance of reaching the target by 2030 of fewer than 50 infections. A CD4 back calculation model estimated an 80% chance of a decline in incidence between 2019 and 2021 in this population.^16^ In contrast to our results, Brizzi and colleagues^32^ estimated a 40% probability of achieving the elimination of HIV transmissions, defined as fewer than one newly acquired infection per 10 000 GBMSM per year, by 2030 (corresponding to fewer than 50 infections among GBMSM).

Interventions leading to around a 30% increase in HIV testing (ie, corresponding to around 400 000 men having tested in the past year), a substantial increase in PrEP (up to 140 000 at its peak, compared with the current 70 000), or decreases in condomless sex (from around 17% of GBMSM having five or more condomless partners in the past year to 5%) would further substantially reduce HIV incidence, by 15%, 23%, and 36%, respectively. A combined substantial increase in HIV testing and PrEP could avert 34% of infections. However, at the current cost-effectiveness threshold, a 16% reduction in the cost of delivery of testing and PrEP would be required for this scenario to offer value for money.

Testing rates have rapidly increased in the UK in recent years, especially in some clinics and in combination with the offer of ART at HIV diagnosis. According to the UKHSA, in 2020, 96% (95% credible interval 94–97) of GBMSM living with HIV in England were diagnosed with HIV.^14^ The COVID-19 pandemic contributed to more people accessing testing remotely. Among GBMSM, the numbers of HIV tests conducted via internet testing tripled from around 32 600 in 2019 to around 97 400 in 2021,^33^ of a total of 178 500 tests conducted through sexual health services. However, to diagnose early, preferably at acute infection, testing those currently underserved is key and so new modalities that increase access and lower barriers to testing, such as stigma, would have to be further expanded. SELPHI, an internet-based, open-label, randomised controlled trial that recruited men interested in HIV self-testing showed that men who were offered free, regular HIV self-testing kits were much more likely to test in the previous 3 months (range 85–88% across surveys) compared with men in the control group (34–42%);^26^ however, given the low incidence in the UK, this did not translate into a difference in diagnoses. Such approaches will have to be expanded in order to achieve such an increase in the uptake of HIV testing.

PrEP awareness has been increasing dramatically among GBMSM and a survey conducted in the UK in 2019 reported that 84% had heard about PrEP.^34^ Among those accessing specialist sexual health services, two-thirds were considered as having a need for PrEP, only four of five of these were identified as such and seven of ten of those in need initiated or continued PrEP.^1^ The main barriers in accessing PrEP through the NHS seem to be: being able to book an appointment for PrEP online, difficulties getting through to clinics by telephone or through email booking systems, and appointment availability. A quarter of those who were able to get through to clinics on the telephone reported being turned away because of lack of appointment availability.^15^ This suggests that the main challenges to increase the number of GBMSM on PrEP are on the supply side. We included the change in condomless sex as a comparator to the other policies considered, but we are not arguing that this change is likely.

Our study has several limitations. First, our estimates were obtained using a mathematical model, the HIV Synthesis Model, which is a simplification of reality. Second, there is uncertainty over some values of parameters used in the model and we have addressed this by sampling a number of parameters from distributions (appendix pp 15–16). Third, because of computer capacity, the population simulated by the model was 4·5% (on average) of the UK GBMSM population, which increases the stochastic variability of our results. To tackle this issue we have presented, for the simulations from 2023 onwards, the mean across simulations starting from the same simulation in 2022. Nevertheless, we cannot exclude the possibility that the variability reported is greater than the variability due to the uncertainty in the parameters and the stochastic variability if we had modelled the whole UK GBMSM population. Fourth, we recognise that when evaluating whether interventions to prevent HIV offer value for money, the quality-of-life measures used might not fully capture all aspects of the sociocultural costs.

In conclusion, our analysis has shown that combination prevention, including a PrEP strategy, played a major role in the reduction in HIV incidence observed so far in the UK among GBMSM. Our model suggests that continuation of current activities should lead to a continued decline; however, the chances of reaching the targets set for 2025 and 2030 of fewer than 250 and fewer than 50 new infections among GBMSM in the UK are very low: 23% and 0%. Enhancing testing and PrEP provision can accelerate the progress to reach zero infections; however, it will be important to reduce costs for testing and PrEP for their continued expansion to be cost-effective. This could at least partially be achieved by expanding self-testing and promotion of event-based PrEP.

## Supplementary Material

Supplementary appendix

## Figures and Tables

**Table:** Summary of assumptions on prevention efficacy

	Effectiveness assumed	Source
Condom	100% (breakage is considered as non-use)	Assumption
Antiretroviral therapy	Effect is mediated through viral load	PARTNER2^23^
Tenofovir disoproxil fumarate plus emtricitabine oral PrEP	86%, as found in the PROUD trial, conducted among GBMSM in the UK; however, the protection conferred by PrEP is assumed to be the same as the level of adherence to PrEP (measured on a scale from 0, meaning no drugs taken, to 1, corresponding to perfect adherence)	PROUD trial^22^ and IPERGAY^24^

GBMSM=gay and bisexual men who have sex with men. PrEP=pre-exposure prophylaxis.

## Data Availability

The model program is available on figshare at https://figshare.com/articles/software/msm_2023_sas/23889750. Because, this is a modelling study, no new primary data were collected. Source code for the models can be found on the HIV Synthesis website.

## Research in context

### Evidence before this study

England has achieved the UNAIDS 95-95-95 targets: in 2021, 95% of people living with HIV were diagnosed, 99% of those diagnosed were on treatment, and 98% of those on treatment had an undetectable viral load. England is committed to achieve less than one newly acquired infection per 10 000 gay and bisexual men who have sex with men (GBMSM) per year (corresponding to fewer than 50 HIV infections) by 2030, guided by an action plan and with investments on HIV testing and prevention. In 2013, two modelling studies reported that HIV incidence among GBMSM in the UK had increased up to 2010, despite high coverage of antiretroviral therapy (ART). Treatment for all at diagnosis was recommended in 2015, following the findings of the START trial, and pre-exposure prophylaxis (PrEP) was progressively introduced among GBMSM from 2016. Since 2015, a decrease in the number of new diagnoses has been observed year on year. However, the extent to which this was due to reduced HIV incidence was unclear, and which factors were the main drivers. Modelling can help to address this question and assess the impact on HIV incidence of continuation of current policies. We searched Web of Science, with no restrictions on language or calendar year, using the search terms: (“Gay and bisexual men” OR “men having sex with men” OR “MSM”) AND “HIV*” AND (“UK” OR “United Kingdom” OR “England”) AND “incidence” on Jan 25, 2023. The search resulted in 82 manuscripts and two were identified as relevant. One estimated the HIV incidence among adult GBMSM between 2009 and 2018 and found a steep decrease in the annual number of new infections among GBMSM, from 2013 to 2015, followed by a steadier decrease from 2016 to 2018. However, these studies did not quantify the contributions of different interventions to decreasing HIV incidence. In terms of projecting future HIV incidence, this study concluded that the probability of achieving elimination of HIV transmission by 2030, defined as less than one newly acquired infection per 10 000 GBMSM per year was 40%. The second study, published in 2015, using the HIV Synthesis Model, investigated what was required to achieve an incidence of 10 per 10 000 person-years by 2030 and concluded that the proportion of all HIV-positive GBMSM with viral suppression needed to increase from below 60% (what it was in 2015) to 90%, assuming no rise in levels of condomless sex.

### Added value of this study

This study uses an existing individual-based model of HIV, calibrated to a wide range of data to the end of 2021 (latest currently available) to identify the contribution of different interventions in reducing HIV incidence so far and to assess the impact on HIV incidence of continuation of current policies and of increases in HIV testing and PrEP in the future. Our modelling suggests that oral tenofovir disoproxil fumarate plus emtricitabine PrEP, increased HIV testing with ART initiation at diagnosis, and reduction in the level of condomless sex each played an important role and that the current HIV incidence would have been double what it is if any one of them had not occurred. We also estimated the chances for the UK to reach the UK Government target of less than 50 newly acquired infections per year among GBMSM by 2030. We predict that this will not be achieved (0% chance) if current rates of testing, ART use, and PrEP use remain as they are. For additional resources to offer value for money, the additional annual budget (compared with the budget for maintaining the current level of interventions) should be £1·62 million.

### Implications of all the available evidence

High levels of HIV testing, ART at diagnosis, and use of PrEP have together resulted in substantial declines in HIV in GBMSM. We project that these will continue, although it is unlikely that the UK Government target will be reached. Further increases in testing and PrEP will accelerate the decline, although costs of these will need to decrease somewhat to remain cost-effective.
