# Supplementary material for: The effect of combination prevention strategies on HIV incidence among gay and bisexual men who have sex with men in the UK: a model-based analysis
Source: Lancet HIV. Author manuscript; Available in PMC 2024 Dec 2. (PMC7616872; doi:10.1016/S2352-3018(23)00204-7)
Supplement: Supplementary appendix [file EMS200027-supplement-Supplementary_appendix.pdf]

THE LANCET
HIV

## Supplementary appendix

This appendix formed part of the original submission and has been peer reviewed. We post it as supplied by the authors.

Supplement to: Cambiano V, Miners A, Lampe FC, et al. The effect of combination prevention strategies on HIV incidence among gay and bisexual men who have sex with men in the UK: a model-based analysis. *Lancet HIV* 2023; **10:** 713–22.

# Appendix of the manuscript “The impact of combination prevention strategies on the HIV incidence among gay and bisexual men in the UK: a model-based analysis”

## Contents

Appendix I. Supplementary material ....................3
1 Brief description of the mathematical model used ....................3
2 Further details ....................3
2.1 Counterfactual scenarios (2012-2022) ....................3
2.2 Scenarios compared going forward (2023-2103) (expanded version) ....................3
2.3 Assumptions on disability weights and costs ....................8
2.4 Inflation....................11
Appendix II. Calibration approach of the Synthesis Model ....................12
Appendix III. Technical details of the Synthesis Model ....................21
1 Demography, sexual risk behaviour and transmission of HIV ....................21
1.1 Demographic model ....................22
1.2 Model of sexual risk behaviour and risk of HIV acquisition....................23
1.3 Tables and figures referred to in Part 1 ....................29
2 Untreated HIV infection....................32
2.1 Fixed parameters at time of infection ....................33
2.2 Determination of viral load....................34
2.3 Determination of CD4 count....................35
2.4 CDC B / AIDS / Death ....................36
2.5 Independent effects on all rates, except death from non-HIV causes (i.e. AIDS / CDC B Symptoms / HIV-related deaths) ....................37
2.6 Rate of occurrence of death from non-HIV causes ....................38
2.7 Summary table of rates (per year)....................39
2.8 Model fits which are relevant to the natural history ....................40
3 Effect of ART ....................43
3.1 Adherence....................44
3.2 Use of antiretroviral therapy (ART) ....................46
3.3 Effect of ART ....................46
3.4 Model fits which are relevant to the effect of ART ....................56
4 HIV drug resistance....................59
4.1 Resistance mutations; introduction ....................60
4.2 ART-naïve patients....................61
4.3 Patients on ART....................62
4.4 Model fits which are relevant to resistance ....................75
5 Other details ....................77

5.1 Diagnosis of HIV ..... 78
5.2 Management of people living with HIV in care ..... 79
5.3 Interruption of ART ..... 81
5.4 Toxicities ..... 85
5.5 Regimen switching ..... 87

# Appendix I. Supplementary material

## 1 Brief description of the mathematical model used

A dynamic individual-based simulation model (the HIV Synthesis Model) is used to simulate longitudinal changes in patterns of condomless anal sex over time and, for those acquiring HIV, HIV progression and treatment outcomes for a random sample of the GBMSM population in the UK, since the start of the HIV epidemic in 1980. The calibration approach and technical details of the model have been previously described in detail (1, 2) and are presented respectively in Appendix II and III. In brief, the model takes into account age, condomless sex with long- term (primary) and short-term (casual) partners, presence of other STIs, HIV testing patterns, and then in those infected with HIV, viral load, CD4 count, use of specific antiretroviral drugs, adherence, presence of specific resistance mutations, risk of AIDS and death, including death from non-AIDS conditions. Such variables are updated in three month time periods. The parameter values determining sexual behaviour, the transmission rate, testing patterns, and the extent to which HIV diagnosis leads to a reduction in condomless sex, are sampled from distributions when calibrating the model. The resulting outputs from the model were compared with various data sources - in all we ran the model over 300,000 times and selected 302 sets of parameter values that are able to reproduce an epidemic close to that observed. These 302 sets of parameter values that lead to outputs that fit closely to observed data were re-used when projecting forward. Basically instead of sampling across all the simulation we sampled from one of the 302 set of parameters producing close fit to the data.

## 2 Further details

### 2.1 Counterfactual scenarios (2012-2022)

1) HIV testing rates stopped increasing in 2012 and the policy of ART at diagnosis was not introduced in mid-2015 (therefore the probability of starting ART per 3 months for people with CD4 count > 350 continued to be 0.075; (see Supplementary Figure 2c and 2d)
2) PrEP was not introduced in 2013 (through PROUD, self-sourcing, the Impact trial and lately general commissioning) with consequent lower levels of testing (as people on PrEP are assumed to be testing every 3 months) and ART initiations (see Supplementary Figure 2e and 2c).
3) Condom use was low from 2012, at levels similar to those observed in 1980 (21% of GBMSM having condomless sex partner(s) in the last year with 5 partners or more compared to on average 14% over 2013 – 2020 in our reference scenario, see Supplementary Figure 2b)
4) HIV testing rates stopped increasing in 2012 and policy of ART at diagnosis not introduced in mid-2015 and PrEP not introduced (see Supplementary Figure 2c-2e).

### 2.2 Scenarios compared going forward (2023-2103) (expanded version)

1) No change in interventions - sexual behaviour, HIV testing behaviour, and the probability of being on ART, and of initiating and remaining on PrEP are fixed to the level reached in 2022 (note that this does not mean that the proportion on men on ART, PrEP etc necessarily stays at 2022 levels) and that PrEP use stops if the overall HIV incidence among GBMSM population drops below 1/10,000; this also has an effect on the number of HIV tests.
2) Increase in the rate of HIV testing, so that around 400,000 men have had a test in the past year (see Figure 4a in the manuscriptSupplementary Figure 3);
3) Increase in PrEP use, so that in 2034 (at its peak) around 160,000 GBMSM are on PrEP (see Figure 4b in the manuscript);
4) decrease in the level of condomless sex (to 1983 levels, the lowest observed), such that the proportion of GBMSM who had at least one condomless sex partner in the last 3 months is reduced to 9% (compared with 24% without the change); This could be achieved through interventions to reduce HIV

transmission risk behaviours, mainly through promotion of condom use and reduction in the number of partners

5) Increase in the rate of HIV testing, as described above combined with an increase in PrEP use

**Supplementary Figure 1. Other model outputs from 1980 to 2022 beyond those shown in Figure 1 of the main manuscript. Line is median over model runs, shading represents 90% uncertainty interval.**

(a) Number of men tested for HIV per year

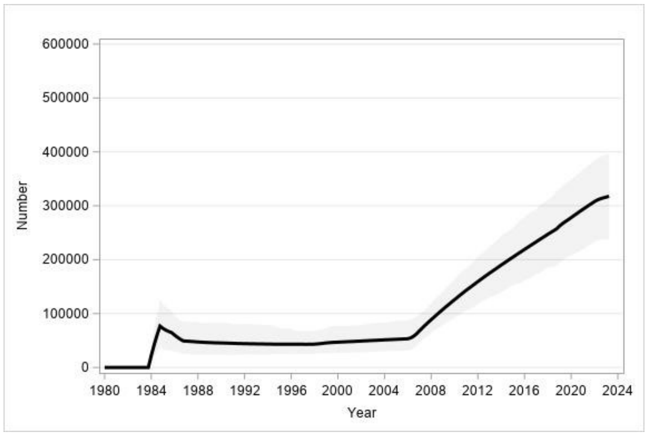


(b) Number of men living with undiagnosed HIV

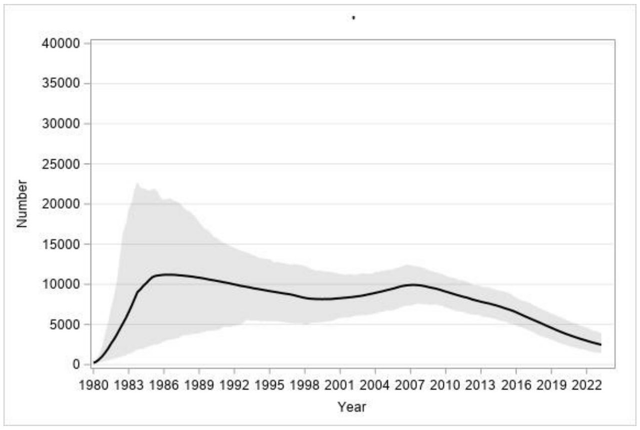


(c) Proportion of men on ART with CD4 count < 350 /mm$^3$

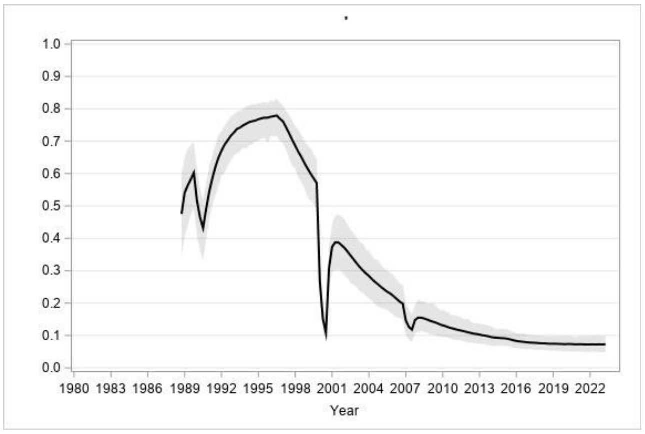


(d) Proportion of men having 5 or more condomless sex partners in the past year

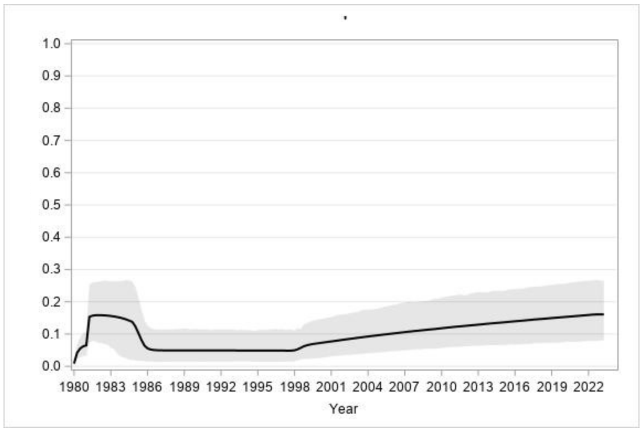


(e) Proportion of all men with HIV with VL <50 copies/ml

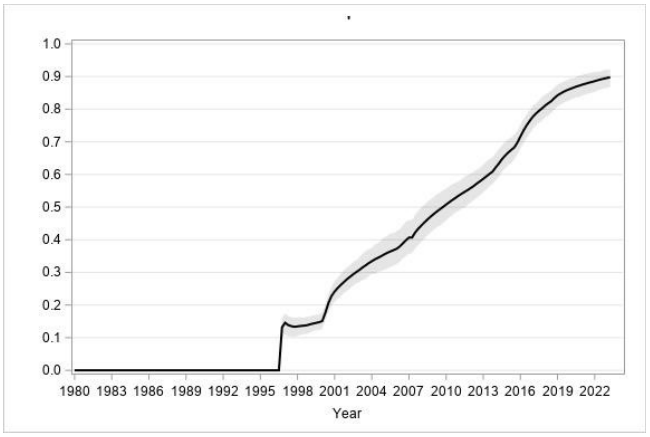


(f) Proportion of men having condomless sex with a person not resident in the UK

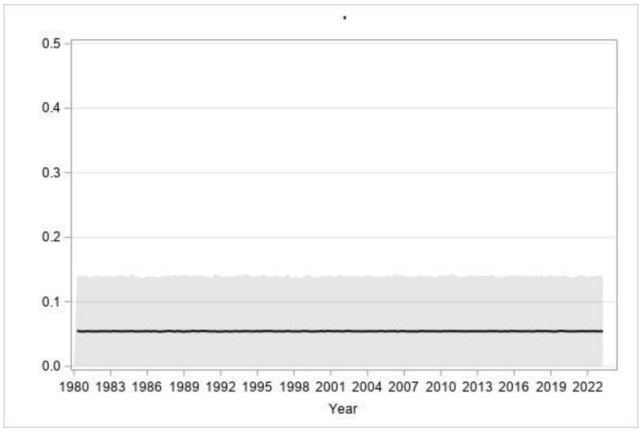

(g) Proportion of new infections from men not resident in the UK

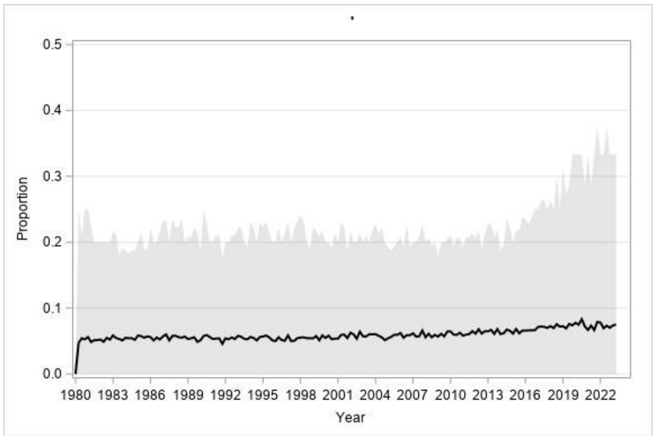


(h) Number of new diagnoses of AIDS per year

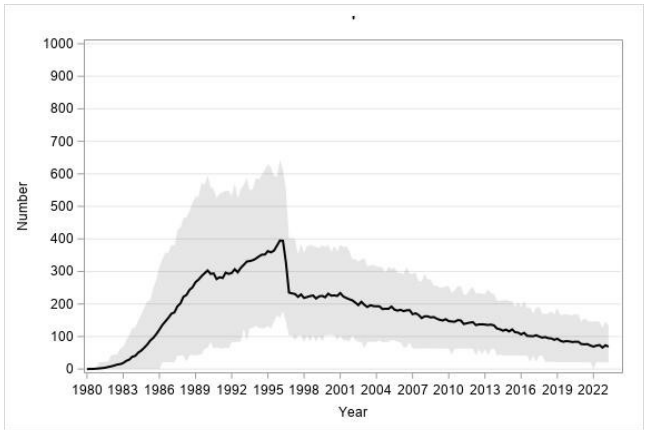


(i) Proportion of men with HIV who are diagnosed

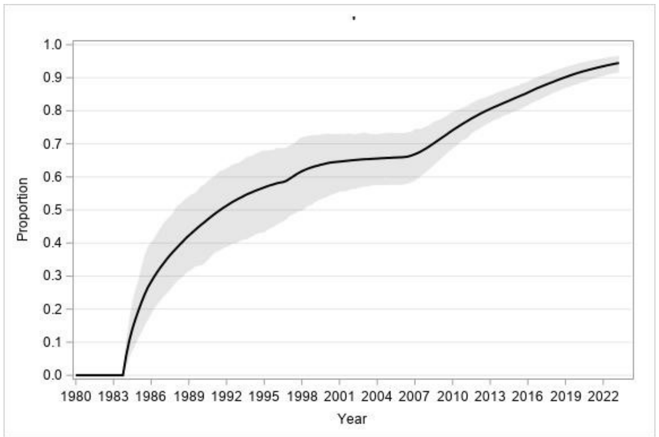


(j) Proportion of men on ART of those diagnosed

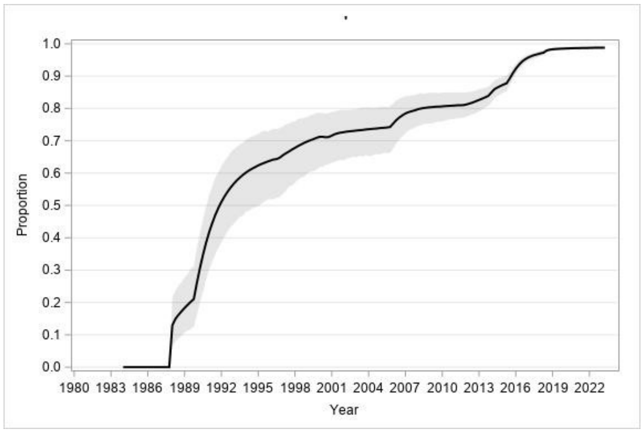


(k) Number of gay and bisexual men living in UK

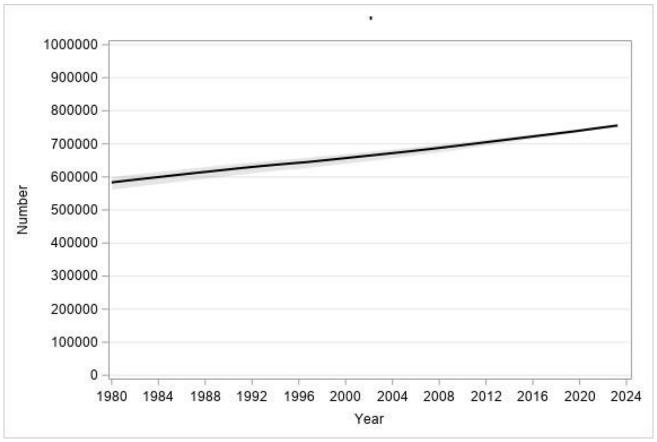


(l) Number of men living with HIV

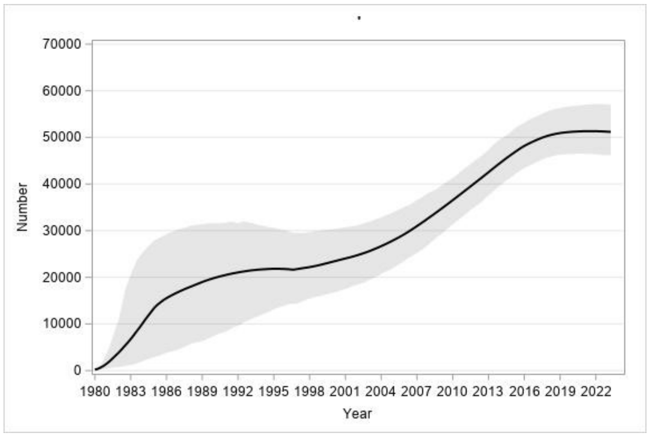

**Supplementary Figure 2. Other model outputs from 2012 to 2022, beyond those in Figure 3, for actual and counterfactual scenarios. Line is median over model runs, shading represents 90% uncertainty interval.**

(a) Proportion of men with HIV who are diagnosed

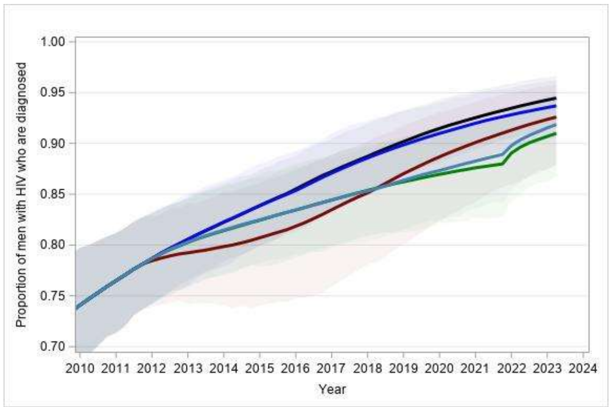


(b) Proportion of men with 5 or more condomless sex partners in past year

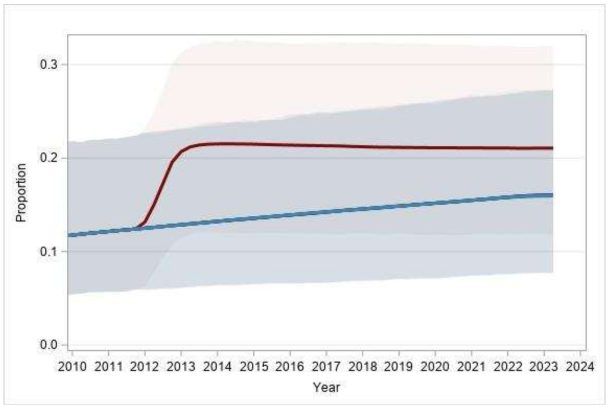


(c) Number of men tested for HIV in the past year

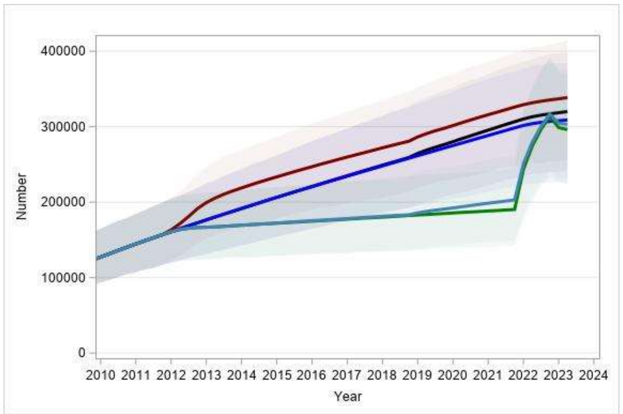


(d) Proportion of men with diagnosed HIV who are on ART

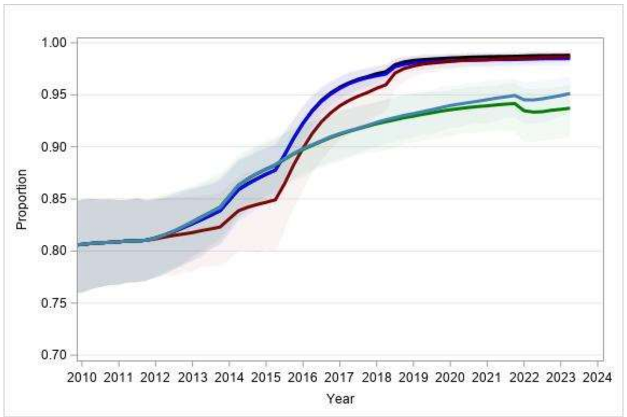


(e) Number of men on PrEP

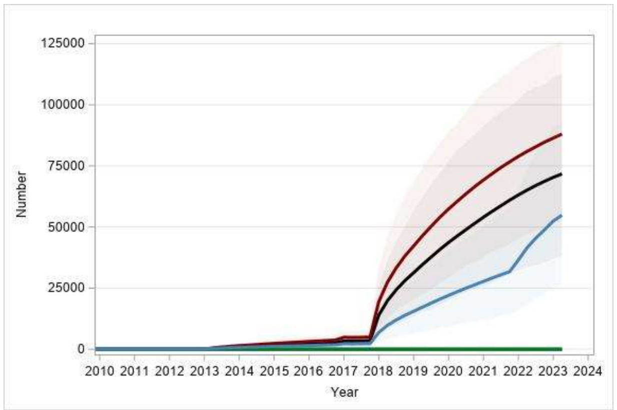


Actual
Counterfactual: no PrEP introduction
Counterfactual: no increase in HIV testing and ART initiation policy remains CD4 count 350 /$mm^3$
Counterfactual: no increase in HIV testing and ART initiation policy remains CD4 count 350 /$mm^3$ and no PrEP
Counterfactual: decrease in condom use to levels in 1980

**Supplementary Figure 3.** Other model outputs from 2023 to 2043 beyond those shown in Figure 4 of the main manuscript. Line is median over model runs, shading represents 90% uncertainty interval.

(a) Proportion of men having 5 or more condomless partners in the past year

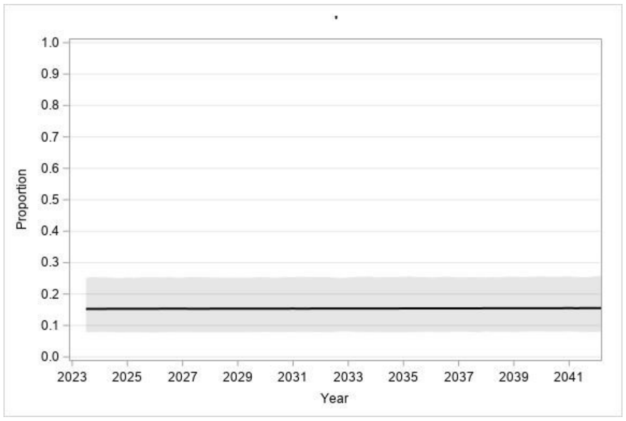


(b) Proportion on ART of those seen for care

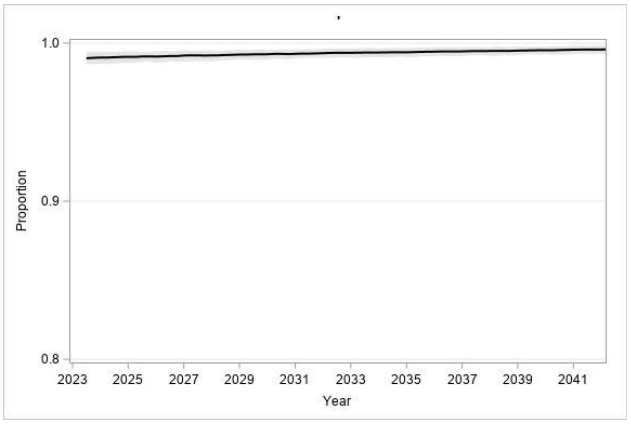


(c) Proportion with VL <50 copies/ml among those on ART

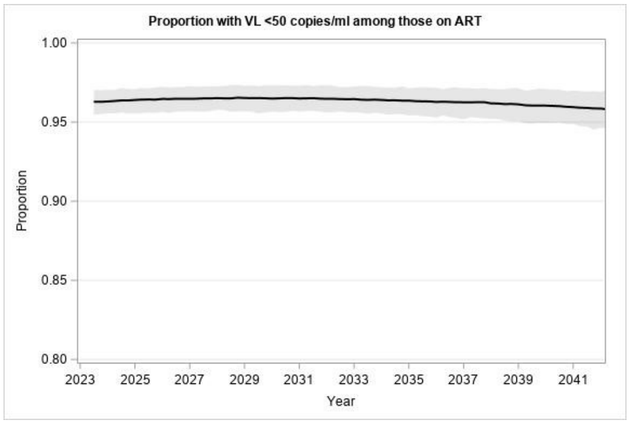

## 2.3 Assumptions on utility weights and costs

The disability weights used are summarized in the table below. In summary they apply to people living with HIV and they depend on the CD4 count and on whether the individual experiences a WHO stage 4 or 3 disease. In addition these utilities will be adjusted by age (-0·004 per additional year starting from an age of 0)(3). The effect of age applies to the whole populations regardless of the HIV status.

**Supplementary Table 1. Assumptions on disability weights**

| Status | Utility weight | Source |
|---|---|---|
| HIV-negative | 1 | Assumption |
| HIV-positive undiagnosed | 1 | Assumption |
| HIV-positive diagnosed with CD4>200 cells/mm$^3$ | 0.9 | (3) |
| HIV-positive diagnosed with CD4≤200 cells/mm$^3$ | 0.85 | (3) |
| HIV-positive diagnosed with HIV with WHO4^ | 0.45 | (4) |
| HIV-positive diagnosed with HIV with WHO3^ | 0.78 | (4) |

^ Applied only in the three month period when experiencing that condition.

Four main components of cost are included:

- Cost of HIV testing
- Cost of clinical care for people living with HIV
- Cost of antiretrovirals for people living with HIV
- Cost of Pre-Exposure Prophylaxis

### 2.3.1 Cost of HIV testing

The cost of HIV testing is assumed to be £31.84 (this value is adjusted to 2019/20; please see section 2.4). This is estimated from sheet “routine GUM clinic” in the document “CONFIDENTIAL DATA HIV PrEP patient management pathway v24082015. It is based on dividing the cost of £115 by 4, which is the cost per attendance (including testing for 4 diseases: Chlamydia, Gonhorrea, Syphillis and HIV).

### 2.3.2 Cost of clinical care for people living with HIV

The estimated cost of clinical care (excluding treatment) for GBMSM living with HIV is estimated using the regression model developed by Alec Miners and copied below. This cost data was analysed using generalised estimating equations (model 3), using STATAs xtgee command and are expressed in 2018/19 value. A gamma distribution and identity link were used to allow for the skewness of the data and because costs are bounded by zero at the lower end (i.e. they cannot be negative). Missing unit costs were estimated using a generalised linear regression model, assuming a gamma distribution and identity-link, based on the length of admission. The results are reported as mean costs per quarter. The regression coefficient for the variables included in the model are indicated in Supplementary Table 2. For further details please search for the manuscript entitled "Estimating the hospital costs of adults diagnosed with HIV in England using routinely collected data" (unpublished data; Miners), under review at the time of submission).

**Supplementary Table 2. Estimated costs per quarter of clinical care for people living with HIV using generalised estimating equations**

| | Model 3 | | |
|---|---|---|---|
| **Category** | **Coef. (£)^** | **95% CIs (£)** | **p-value** |
| **Constant** | 518 | 450; 587 | <0.0001 |
| **Age in years** | - | - | 0.008 |
| 18-30 (base) | - | - | - |
| 31-50 | -77 | -129; -26 | - |
| 51-70 | -72 | -127; -17 | - |
| 71+ | -18 | -101; 65 | - |
| **Gender, sexual orientation and ethnicity** | - | - | <0.0001 |
| GBMSM (base) | - | - | - |
| Black-African heterosexual men | -74 | -120; -29 | - |
| Non black-African heterosexual men | -48 | -97; 2 | - |
| Black-African women | -19 | -61; 22 | - |
| Non black-African women | 39 | -19; 96 | - |
| **IVDU** | - | - | - |
| No (base) | - | - | - |
| Yes | 26 | -57; 109 | 0.54 |
| **Quarterly period+** | -5 | -6; -4 | <0.0001 |
| **Initial 6 months from Trust contact** | - | - | <0.0001 |
| No (base) | - | - | - |
| Yes | 654 | 578; 730 | <0.0001 |
| **CD4 count (cells/µL)** | - | - | <0.0001 |
| ≤50 | 618 | 348; 887 | - |
| 51-200 | 295 | 235; 355 | - |
| 201-500 | 62 | 45; 78 | - |
| 501+ (base) | - | - | - |
| **History of VL failure and current VL (copies/mL)** | - | - | <0.0001 |
| No VL failure history and VL<200 (base) | - | - | - |
| VL failure history and VL <200 | 90 | 61; 119 | - |
| Current VL ≥200 irrespective of VL failure history | 165 | 126; 204 | - |

+where Q1 2010 = 0 and Q4 2017 = 31

*within the model we assumed someone as being in the "Initial 6 months from Trust contact" if this is the first 6 months attending the clinic since being linked to care following diagnosis, or the 6 months of care following an interruption of care.

Cis: confidence interval; IVDU: intravenous drug user; VL: viral load;

As an example the clinical care cost (for a quarter) for a 40 years old GBMSM, who is not an intravenous drug user (IVDU) (this is not modelled and we are assuming none of them is an IVDU), in the first quarter of 2011 ( so quarter number 4), who has started ART 3 months ago, with current CD4 count of 100, VL failure history and current VL of 1000 copies/ml is £1,535, calculated as 518 – 77*1 + 0 + 0 + (-5)*4 + 654*1 + 295*1 + 165*1 = 1,535.

To estimate the cost of clinical care for people who are living with HIV but undiagnosed or diagnosed but ART naive and not in-care we use the regression analysis detailed in Supplementary Table 2. Therefore, for the variable "Initial 6 months from Trust contact" they will have a value of "No", for the "CD4 count" it is assumed that it is "501+" and for the "History of VL failure and current VL" we assumed it is "No VL failure history and VL<200". For those diagnosed not in care but ART-experience the same will apply except for the fact that their current CD4 and viral load will be used to determine the cost. The reason for assuming a cost of clinical care for these people is based on the fact that HIV infection raises the risk of common clinical conditions.

These costs are then adjusted for inflation to the 2019/20 (please see section 2.4).

### 2.3.3 Cost of antiretrovirals for people living with HIV

The annual cost of antiretroviral drugs for people living with HIV currently on treatment is assumed to be £2,667. This was calculated as the weighted average based on the distribution of regimens among GBMSM on treatment on the 1st January 2018 in the UK CHIC data (personal communication; see Supplementary Table 3) and applying the cost from the drugs and pharmaceutical electronic market information tool (eMIT; https://www.gov.uk/government/publications/drugs-and-pharmaceutical-electronic-market-information-emit), if available or the British National Formulary if not (https://bnf.nice.org.uk/).

**Supplementary Table 3. Distribution of antiretroviral regimens among GBMSM on treatment in the UK CHIC data and their cost**

| Regimen | n | % | Annual cost | Source | Number of pills |
|---|---|---|---|---|---|
| Tenofovir + Emtricitabine + Efavirenz | 3,231 | 23.9% | £328 | emit 2022 | 1 |
| Tenofovir + Emtricitabine + Rilpivirine | 1,832 | 13.6% | £2,622 | BNF | 2 |
| Tenofovir + Emtricitabine + Raltegravir | 1,822 | 13.5% | £3,053 | emit 2022 & BNF | 2 |
| Abacavir + Lamivudine + dolutegravir | 1,722 | 12.7% | £6,300 | emit 2022 & BNF | 2 |
| Tenofovir + Emtricitabine + Ritonavir + Darunavir | 824 | 6.1% | £803 | emit 2022 | 3 |
| Tenofovir + Emtricitabine + Nevirapine | 757 | 5.6% | £248 | emit 2022 | 2 |
| Tenofovir + Emtricitabine + Darunavir + cobicistat | 660 | 4.9% | £4,046 | emit 2022 & BNF | 2 |
| Tenofovir + Emtricitabine + elvitegravir + cobicistat | 587 | 4.3% | £10,708 | BNF | 1 |
| Abacavir + Lamivudine + Raltegravir | 455 | 3.4% | £3,098 | emit 20212 & BNF | 2 |
| Abacavir + Lamivudine + Efavirenz | 445 | 3.3% | £289 | emit 2022 | 2 |
| Tenofovir + Emtricitabine + Ritonavir + Atazanavir | 338 | 2.5% | £654 | emit 2022 | 4 |
| Tenofovir + Emtricitabine + Dolutegravir | 327 | 2.4% | £6,256 | emit 2022 & BNF | 2 |
| Abacavir + Lamivudine + Nevirapine | 283 | 2.1% | £292 | emit 2022 | 2 |
| Ritonavir +Darunavir | 236 | 1.7% | £619 | emit 2022 | 2 |
| Total | 13,519 | 100% | | | |

### 2.3.4 Cost of HIV Pre-Exposure prophylaxis

**Supplementary Table 4. Cost components of Pre-Exposure prophylaxis**

| Item | Mean cost (adjusted to 2019/20) | Source for the cost assumed |
|---|---|---|
| Antiretrovirals for PrEP (per 1 year of daily use) | £183.31 | The cost of 365.25 Truvada pills is £183.31 in the eMIT 2022. This is multiplied by the actual use, given it is offered as event-based, to obtain the actual cost. |
| Additional cost of monitoring people on PrEP in the 1st year compared to the following years | £90.54 | £82 is the additional cost compared to GBMSM at similar risk (£176 over the 1st 12 months)^^ This was then adjusted for inflation. |
| Monitoring of people on PrEP per year (following the first year) | £103.79 | £94 is the additional cost compared to GBMSM at similar risk^^. It included HIV test, CT/GC 3 site plus syphillis test, Hep A/B test, creatinine, staff time for PrEP assessment, check of contraindication, drug management, counselling. This was then adjusted for inflation. |
| Cost of visit to prescribe Post Exposure Prophylaxis | £248.44 | £225 is the Sexual health clinic tariff for PEP(5). This was then adjusted for inflation. |
| Post Exposure Prophylaxis course (PEP;28 days regimen) | £486.47 | It includes the cost of the antiretrovirals used for PEP: Truvada (£15.06 for 30 pills; Source: Emit 2022); and Raltegravir (£471.41 for 60 pills) from the BNF (last updated December 2022; https://bnf.nice.org.uk/medicinal-forms/raltegravir.html; accessed 27/01/2023). |

^^based on PHE calculation using GUM pathway for PrEP from the PROUD trial protocol and collaborated with updated laboratory pathology prices (two PHE laboratories, and a University College London Provider to provider price list) and consumables prices obtained from NHS England Supply Chain.

eMIT: Drugs and pharmaceutical electronic market information; GUM: Genito Urinary Medicine; PEP: Post-Exposure Prophylaxis; PrEP: Pre-Exposure Prophylaxis;

When considering the impact of introducing PrEP the cost of the potential social campaigns to achieve that uptake are not considered.

## 2.4 Inflation

The costs were adjusted for inflation to the value in 2019/20 using the annual % increase in HCHS pay and price until 2015/2016 and in NHSCII pay and price from 2016/17 (page 163 (6)

## Appendix II. Calibration approach of the Synthesis Model

The Synthesis model is calibrated using an Approximate Bayesian Computation Approach(7). We consider distributions for the parameters that determine levels of condomless sex to reflect uncertainty in the value (26 parameters in total are sampled from distributions in this way). For each of over 300,000 runs of the simulation model we sample from each of these distributions to obtain a set of parameter values to be used and generate a full reconstructed epidemic scenario for 1980-2022. We then consider the parameter sets that provide the closest calibration to the observed data.
The sampling from distributions for the parameters relating to sexual risk behaviour and transmission rate means that we explore a range of possible scenarios, including ones in which the ratio of short to long term partners increases or decreases or the number of short term partners occurring in the same period as a man has a longer term partner (concurrency) increases or decreases.
In any given period, the probability of an uninfected person having a condomless sex partner who is infected with HIV depends on their number of partners and on the prevalence of HIV amongst partnerships formed by other men in the population, accounting for patterns of age mixing. On exposure to an infected partner, the probability of transmission depends on the viral load level of the partner (obtained by sampling from the distribution of viral load levels in partnerships formed by HIV infected GBMSM, accounting for age), the estimated risk of transmission at that viral load and presence of a concurrent sexually transmitted infection. We assume a very low rate of transmission (0.00001 per 3 months) when the viral load is undetectable(8) but, again, we consider a distribution of possible values reflecting uncertainty(1). For GBMSM who have become infected with HIV the variables modelled include; primary infection (a period of raised infectivity of duration 3 months), viral load, CD4 count, adherence to ART, risk of AIDS and death. Resistance acquisition and transmission is also incorporated, and its effects accounted for, although this is not a focus in the present paper. The model of progression of HIV and the effect of ART has been shown to provide a generally close fit to observed data relating to natural progression of HIV infection and the effect of ART(1, 9-12). Based on data from NATSAL(13, 14), we assume that in mid-2016 there were 586,355 GBMSM aged 15-64. This estimate is obtained as the product of the male population age 15-64 in mid 2016 (21,036,300)(15) and the proportion who reported at least one sexual partner of same sex in the past 5 years among 16-64 (2.79%)(16). As for the UK population as a whole, we assume the GBMSM population increases in size in line with the population increase.

Details of distributions for all parameters sampled in the calibration process are given in Supplementary Table 5. Seven parameters determine the sexual behaviour: relative average sexual behaviour with short-term partners ($\mu_7$), the skewness in the distribution of number of short-term condomless sex partners ($\mu_2$ and $\mu_3$, with the former determining the most extreme numbers of short-term sexual partners), the rate with which new long term condomless sex partnerships are formed ($\mu_4$), and the proportions of men who have a lifetime reduced likelihood of short term condomless sex partners ($\mu_6$). A correlation is induced by the sampling of the parameter $\mu_7$ in order to provide a focus on parameter space most likely to give low values of the overall fit. For example, if the sampling of $\mu_1$-$\mu_5$ (see below for description of $\mu_1$ and $\mu_5$) is such that values chosen are at the higher end of the distribution and $\mu_6$ is at the lower end of the distribution then the simulation run will produce an epidemic which is too large, unless there is some compensation when selecting the value of $\mu_7$. To indicate what these distributions mean in terms of the proportion of men with a condomless anal sex partner (short or long term) in the past year, they are such that the distribution ranges from 0.10 to 0.92 (90% uncertainty interval: 0.22-0.50) in 1995.

Parameters relating to transmission are the rate of transmission through anal sex per 3 months for a short term condomless sex partner with VL<2.7 log ($\pi_1$), for a short term partner in primary infection ($\pi_2$), the fold change in transmission rate per 3 months for a given VL ($\mu_1$) with a distribution representing the level of uncertainty and the fold higher risk of transmission per 3 months from a long term partner compared with short term (due to more sex acts - $\mu_5$). As illustrated in Supplementary Figure 4, we specified changes in relative sexual risk behaviour (compared with that in 1980) from 1980 to 1998, with a decline in the early 1980's (as was observed (17)), followed by a prolonged period to 1998 of reduced sexual risk behaviour. There is evidence of significant increases in sexual behaviour in the late 1990's(18-21), and likewise there was likely an increase in testing in 2001 with introduction of opt-out testing in GUM clinics(22). We therefore parameterized changes in sexual risk behaviour and testing assuming a step increase in 1998 for sexual risk behaviour followed by a linear change,

and a linear change in underlying testing rate from 2001. Parameters relating to natural history of HIV and the effect of ART were fixed, using values which have previously been shown to give a close fit to observed data(10, 11).

Model outputs were formally compared with data obtained as part of HIV surveillance activities carried out by the UK Health Security Agency (former Public Health England) for each year between 2011 and 2021 included, unless stated:

- proportion of men having at least one condomless sex partner in the past year in 2000 and 2010
- Proportion of GBMSM reporting having tested for HIV in the past year in 2010
- Number of HIV diagnoses per year for GBMSM aged 15-64 years in the following periods: 1984-1998, 1999-2002, 2003-2005, 2006-2008, 2009-2010, 2011-2012 and then in each year between 2013 and 2020
- proportion diagnosed within 4 months of infection (within 6 months up to 2013) in each year between 2011 and 2018
- Proportion with CD4 count below 350 at diagnosis in each year between 2011 and 2020
- Numbers of GBMSM aged 15 years and older seen for care for HIV between 2011 and 2020
- Proportion on ART of those seen
- Proportion starting ART within 3 months of diagnosis in each year between 2011 and 2018 (included)
- Proportion of men on ART with CD4 count <350
- Proportion with VL <50 copies/ml among those on ART

Supplementary Table 6 shows the observed values of these data. For each data item the fit was calculated as: $\frac{|O-M|}{O}$, where O indicates the observed value and M the model output value. We divided here by the observed rather than the model output value as this provided greater stability. The overall fit was calculated as the sum of the fit to each of the data items with the weights shown in Supplementary Table 6. The weights were chosen a priori, in discussion with Public Health England (now UK Health security agency) and chosen to reflect considerations of the importance placed that on the model fitting the various different data items and the confidence in the data.
The model was run >300,000 times, independently sampling the 26 parameter values relating to sexual behaviour, testing and transmission(1) from the distribution indicated in Supplementary Table 5. We recorded parameter sets which provided a fit value <6. To generate uncertainty bounds we present the median and 90% uncertainty interval (5%-95% centiles) over runs in this fit value range. 522 model runs to 2023 with close model fit were identified and then we selected at random from these runs to move from 2023 on to 2104. This was done 1000 times.

**Supplementary Figure 4. Parameterization of trend in condomless sex (CLS) with short term partners**

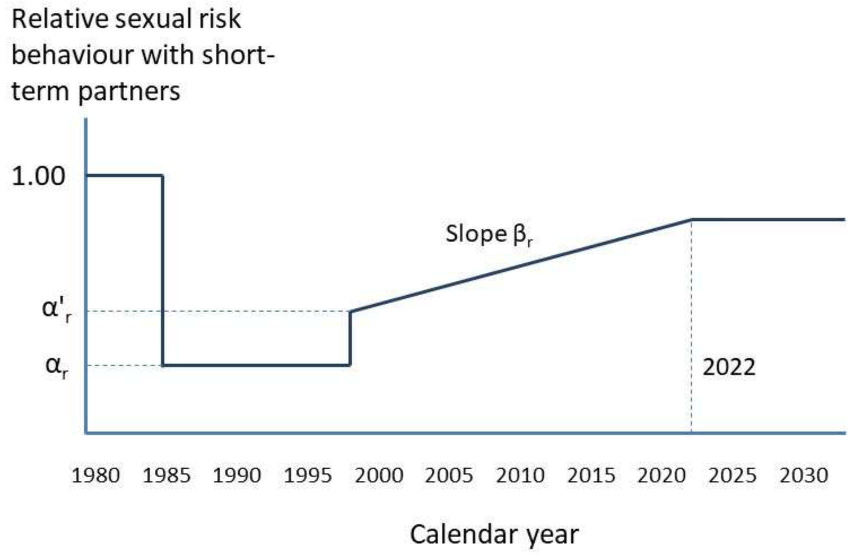


**Supplementary Figure 5. Parameterization of trend in HIV testing rate**

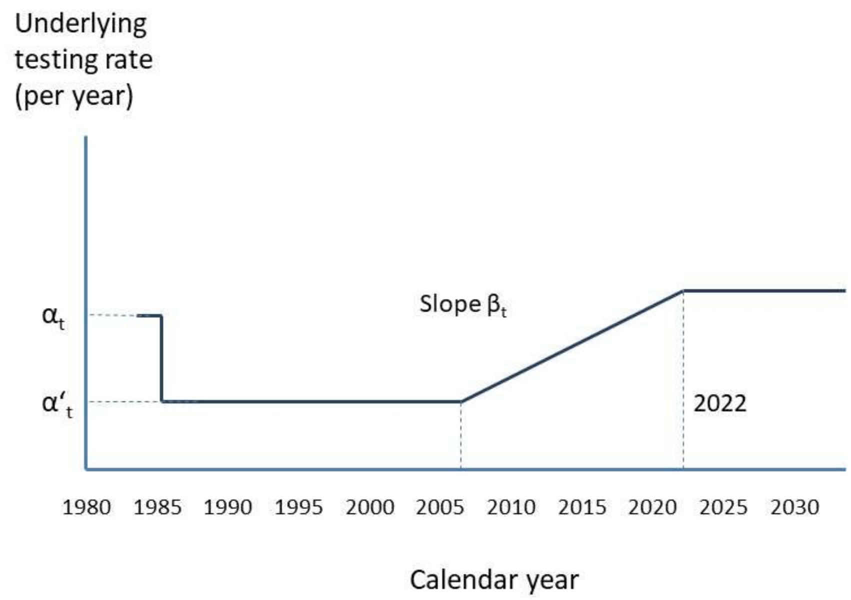

**Supplementary Table 5. Parameter values and distributions sampled as part of the calibration process, reflecting uncertainty in parameter values⁺.**

| Parameter [*name in SAS code*] | Value / distribution | Basis for choice |
|---|---|---|
| **Parameters related to population growth** | | |
| Category of population growth [*inc_cat*] | 1: 33%<br>2: 33%<br>3: 33% | Range of assumptions to reflect uncertainty(23) |
| **Parameters related to sexual risk behaviour and transmission** | | |
| Fold change in infectivity for given VL ($\mu_1$) [*zx*] | exp (Normal(0, 0.25) | |
| Value of multiplicative factor determining numbers of partners for those in highest short term partner group (µ2; see Appendix III) [*swn*] | 7 x exp (Normal(0, 0.25) | (13, 24-30) |
| Value of and fold change in multiplicative factor partners for those in second highest short term partner group (µ3; see Appendix III) [*newp2_10_p*] | 3.5 x exp (Normal(0, 0.25) | |
| Probability of new long term condomless sex partners (3 months) (µ4) [*eprate*] | 0.06 x exp (Normal(0, 0.25) | Assumption |
| Fold higher risk of transmission to long term condomless sex partner compared with short term (due to a greater number of sex acts) (µ5) [*fold_ep_tr*] | 4 x exp (Normal(0, 0.25) | Assumption & preliminary fitting~ |
| Proportion of individuals with a 50% (75%) reduced lifetime likelihood of short term partners (µ6) [*prop_life_red_rb*] | 0.4 x exp (Normal(0, 0.25)<br>(above value / 2) | Assumption & preliminary fitting~ |
| Factor determining median number of short term partners (µ7) * [*newp_factor*] | ( µ6 /(0.4*µ1*µ2/7*µ3/0.06*µ4/0.06*µ5/4)x exp (Normal(0,0.10) | Preliminary fitting~ |
| Fold change in rate of transmission when viral load undetectable for short term partners (per 3 months) [*ui*] | exp (Normal(0,0.35) | Perceived uncertainty |
| αr (see Supplementary Figure 4, it applies from 1985 to 1998)^ [*crb83*] | 0.27 x exp (Normal(0, 0.35)) | (17), Preliminary fitting~ |
| α'r (see Supplementary Figure 4, it applies in 1998) [*crb98*] | 0.35 x exp (Normal(0, 0.35)) | Preliminary fitting~ |
| βr (see Supplementary Figure 4) [*beta_r*] | Normal (0.03, 0.015) | (18-21, 31) |
| Proportion of men who substantially reduce number of short term partners (by 90%) after HIV diagnosis [*ch_risk_diag_newp*] | Beta (3,7) | (30, 32) |
| Proportion of men who substantially reduce probability of condomless sex with long term negative partner (by 90%) after HIV diagnosis [*ch_risk_diag*] | Beta (3,7) | (32) |
| **Parameters related to testing and diagnosis** | | |
| αt (see Supplementary Figure 5, it applies in 1984 and 1985) [*rt_84_85*] | 0.03 x exp (Normal(0, 0.25)) | Preliminary fitting~ |
| α't (see Supplementary Figure 5, it applies from 1986 to 2005) [*rt_86_00*] | 0.28 x exp (Normal(0, 0.25)) | Preliminary fitting~ |
| βt (see Supplementary Figure 5, it applies from 2006 to 2021) [*beta_t*] | Normal (0.005, 0.003) | (22) |

| | | |
|---|---|---|
| Fold reduction in probability of testing if no condomless sex partner in past year [*lt*] | Normal (3.5, 1.0) (left truncated at 0.1) | Assumption, based on preliminary fitting~ |
| Fold increase in probability of testing if at least one condomless sex partner in past year [*ht*] | Normal (3.0, 1.0) (left truncated at 0.1) | Assumption, based on preliminary fitting~ |
| Proportion with reluctance to test (4-fold reduced testing rate) [*rate_hardreached*] | 0.25 x exp (Normal(0, 0.25) | (13, 31) |
| Proportion not testing (if no AIDS) [*rate_noreached*] | 0.25 x exp (Normal(0, 0.25) | (31) |
| **Parameters related to PrEP** | | |
| Uptake PrEP [*uptake_prep_max*] Note: this is further reduced depending on the number of partners, presence of STI and PEP use | Beta (4,1) | |
| **Parameters related to treatment and transmission of resistant mutations** | | |
| Inter-patient variation in rate of CD4 rise - when CD4 is rising [*patient_cd4_rise_art*] | exp(Normal(0,0.2)) | |
| Multiplicative factor to determine the probability of resistant mutations not being transmitted, if a person gets infected from a person with resistant mutations in majority virus [*res_trans_factor*] | Uniform (0.5,1) | |
| **Parameters related to risk of HIV infection from partners not resident in the UK** | | |
| RR that the the CLS partner, if from abroad, is not virally suppressed, compared to a UK resident partner *[external_exp_factor]* (This applies only to 50% of simulations) | Uniform (1.5,2.5) | Based on the fact that in 2018 among GBMSM the proportion of PLHIV with VL suppressed was: 88% in the UK an 75% in Europe (33) |
| Probability per 3 months that the CLS partner is not a UK resident [*rate_exp_set_lower_p_vl1000*] | Uniform(0.05, 0.15) | |

+ Further details on parameter values is given in Appendix III. Distributions to some extent reflect perceived level of uncertainty, which are subjective. Parameter values are derived directly from the cited literature in some cases but in most the cited literature did not present estimates of parameter values exactly corresponding to parameterization of the model and was thus used to inform the presence and approximate magnitude of relationships but not to directly provide the parameter value used. Nonetheless, our inferences are based on sets of parameter values which result in a good fit to the data, as illustrated in Figure 1 in the manuscript. The resulting fit of the model to data on HIV natural history and the effect of ART is shown in Appendix III.

***** the correlation induced by the sampling of this parameter is to provide a focus on parameter space most likely to give low values of the overall fit. For example, if the sampling of $\mu_1$-$\mu_5$ is such that values chosen are at the higher end of the distribution and $\mu_6$ is at the lower end of the distribution then the simulation run will produce an epidemic which is too large, unless there is some compensation when selecting the value of this parameter.

^ The exact timing (between 1983 and 1985) of the reduction in sexual risk behaviour in the 1980s is dependent on the number of people infected such that the reduction occurs when 7000 men have been infected. This is due to the stochastic run-to-run variability (for the same parameter value set).

**~** preliminary fitting refers to the informal stage of model building from our highly parameterized starting model in which the structure is derived, based on a decision to hold certain parameters fixed and thus form part of the structure, and the plausible range of values for other parameters is ascertained. This process of how to simplify a complex model into a simpler one to address a given question is in many ways equivalent to the process of building a new model to address a given question.

**Supplementary Table 6. Fit value used to measure model calibration to observed data, with observed data.**

Note: The number tend to change slightly from year to year, we are going to use the most recent. We are going to give each item the weight indicated in the column, therefore the number of data point does not affect the overall weight of a certain item.

| **Data items used in the calibration score** | | | |
|---|---|---|---|
| **Data item** | **Weight** | **Year** | **Observed Value/Range** |
| Proportion of GBMSM reporting condomless anal sex in the past year[a] | 0.1 | 2000 | 0.37 |
| | | 2010 | 0.44 |
| Proportion of GBMSM reporting having tested for HIV in the past year[a] | 0.3 | 2010 | 0.27 |
| Number of HIV diagnoses (in people aged 15-64)[b] | 0.4 | 1984-98 | 23,384 |
| | | 1999-2002 | 6,587 |
| | | 2003-05 | 7,039 |
| | 0.6 | 2006-08 | 6,958 |
| | | 2009-10 | 5,373 |
| | 0.8 | 2011-12 | 5,806 |
| | | 2013 | 3,001 |
| | 1.0 | 2014 | 3,172 |
| | | 2015 | 3,116 |
| | | 2016 | 2,512 |
| | | 2017 | 2,168 |
| | | 2018 | 2,044 |
| | | 2019 | 1,813 (1,699+114) |
| | | 2020 | 1,032 (918+114) |
| Proportion with CD4 count below 350 at diagnosis[c] | 0.3 | 2011 | 0.35 |
| | | 2012 | 0.36 |
| | | 2013 | 0.30 |
| | 0.5 | 2014 | 0.29 |
| | | 2015 | 0.29 |
| | | 2016 | 0.32 |
| | | 2017 | 0.35 |
| | | 2018 | 0.34 |
| | | 2019 | 0.34 |
| | | 2020 | 0.34 |
| Proportion diagnosed within 4 months of infection[d] | 0.3 | 2011 | 0.23 |
| | | 2012 | 0.27 |
| | | 2013 | 0.30 |
| | 0.5 | 2014 | 0.35 |
| | | 2015 | 0.27 |
| | | 2016 | 0.29 |
| | | 2017 | 0.30 |
| | | 2018 | 0.26 |
| Numbers seen for care for HIV (in people aged 15+)[e] | 0.8 | 2011 | 32,052 |
| | | 2012 | 34,285 |
| | | 2013 | 36,467 |
| | 1.0 | 2014 | 38,838 |
| | | 2015 | 41,328 |
| | | 2016 | 42,899 |

| | | | | |
|---|---|---|---|---|
| | | 2017 | 44,145 | |
| | | 2018 | 44,516 | |
| | | 2019 | 45,771 | |
| | | 2020 | 44,660 | |
| Proportion on ART of those seen[e] | 0.3 | 2011 | 0.84 | |
| | | 2012 | 0.86 | |
| | | 2013 | 0.88 | |
| | 0.5 | 2014 | 0.89 | |
| | | 2015 | 0.94 | |
| | | 2016 | 0.97 | |
| | | 2017 | 0.98 | |
| | | 2018 | 0.98 | |
| | | 2019 | 0.99 | |
| | | 2020 | 0.99 | |
| | | 2021 | 0.99 | |
| Proportion starting ART within 3 months of diagnosis[f] | 0.3 | 2011 | 0.38 | |
| | | 2012 | 0.44 | |
| | | 2013 | 0.49 | |
| | | 2014 | 0.53 | |
| | | 2015 | 0.74 | |
| | | 2016 | 0.76 | |
| | | 2017 | 0.72 | |
| | | 2018 | 0.78 | |
| Proportion of men on ART with CD4 count <350[g] | 0.3 | 2011 | 0.17 | |
| | | 2012 | 0.14 | |
| | | 2013 | 0.12 | |
| | | 2014 | 0.13 | |
| | | 2015 | 0.13 | |
| | | 2016 | 0.09 | |
| | | 2017 | 0.08 | |
| | | 2018 | 0.08 | |
| | | 2019 | 0.07 | |
| | | 2020 | 0.06 | |
| | | 2021 | 0.08 (in England among men) | |
| Proportion with VL <50 copies/ml among those on ART[h] | 0.3 | 2011 | 0.90 | |
| | | 2012 | 0.89 | |
| | | 2013 | 0.89 | |
| | 0.5 | 2014 | 0.93 | |
| | | 2015 | 0.94 | |
| | | 2016 | 0.95 | |
| | | 2017 | 0.94 | |
| | | 2018 | 0.95 | |
| | | 2019 | 0.95 | |
| | | 2020 | 0.98 | |
| | | 2021 | 0.98 | |
| **Data used to inform the model but not directly in the calibration** | | | | |
| Number of HIV diagnoses (in people aged 15+)[b] | NA | 2021 | 1081 | |
| Proportion with CD4 count below 350 at diagnosis[c] | NA | 2021 | 0.40 | |
| Numbers seen for care for HIV (in people aged 15+)[e] | NA | 2021 | 43,371 | |
| | NA | 2012 | 25 | |

| | | | | |
|---|---|---|---|---|
| Number on PrEP (by the end of the year unless indicated)[i] | | 2013 | 250 | |
| | | 2014 | 500 | |
| | | 2015 | 500 | 885 |
| | | 2016 | 500 | 3,540 |
| | | 2017 | 4000 | 11,200 |
| | | 2018 | 10,000 | 17,700 |
| | | 2019 | 10,000 | 35,400 |
| | | 2020 | ----- | ----- |
| | | 2021 | ----- | 59,236 |
| Number of GBMSM tested for HIV in the last year in the UK[j] | NA | 2010 | 51,000 | |
| | | 2011 | 64,270 | |
| | | 2012 | 72,699 | |
| | | 2013 | 79,598 | |
| | | 2014 | 93,286 | |
| | | 2015 | 101,612 | |
| | | 2016 | 106,274 | |
| | | 2017 | 116,071 | |
| | | 2018 | 132,770 | |
| | | 2019 | 157,710 | |
| | | 2020 | 146,900 | |
| | | 2021 | 178,000 England | |
| Number of HIV tests among GBMSM in the UK [k] | NA | 2009 | 60,000+8210[h] | |
| | | 2010 | 68,000+9109[h] | |
| | | 2011 | 84,000 | |
| | | 2012 | 94,000 | |
| | | 2013 | 106,000 | |
| | | 2014 | 130,000 | |
| | | 2015 | 143,000 | |
| | | 2016 | 148,000 | |
| | | 2017 | NA | |
| | | 2019 | 261,582 | |

Note: All data are from Public Health England, except where stated. We compared the outputs of the final model with a larger array of data .

a - NATSAL 2000(13) and 2010 (16) .

b - For the period 2010-2018 the source is Table 13 of Key_Population_Tables_2020 available from https://www.gov.uk/government/statistics/hiv-annual-data-tables#history [accessed 04/11/2020] and it refers to the age 15-64. For 2019-2020 the source is Table 13 of Key_Population_Tables_2021 available from https://www.gov.uk/government/statistics/hiv-annual-data-tables [accessed 06/01/2022] , which does not include Wales, Northern Ireland and Scotland, so for 2019 I added the value reported in the previous report for these 3 countries (n=114) and assumed they stayed the same in 2020. For 2021 the source is "Table 1c: New HIV diagnoses by probable exposure category and gender" in file "2022-HIV-country-region-data-tables-version-2 -.ods" [accessed 28/12/2022]. Before 2003 the diagnoses for gay and bisexual men were not reported specifically for the age group 15-64, but the number of diagnoses outside of this age range was likely to be very low.

c - In the previous calibration median CD4 at diagnosis was included instead; for the years 2005, 2008, 2010 and 2012. The source is Table 13 of Key_Population_Tables_2021 available from https://www.gov.uk/government/statistics/hiv-annual-data-tables [accessed 06/01/2022]. In the main report the % of late diagnosis (corrected) is reported which is a lot lower. For 2021 the source is sheet "Men_care" in file "2022-HIV-key-populations-data-tables-version-2no

d - Information not available in; Information for 2011,2014 and 2018 available in (34); for 2017 in (35); for 2016 in (36); for 2015 (37); for 2012 and 2013 (38); Until 2013 by 6 months, from 2014 by 4 months; In the previous calibration years 2009 and 2010 were included.

e - Data from Table 15 (39). In the previous calibration for number seen for care the values in 2005, 2010 and 2012 were included. For 2020 Table 13 of Key_Population_Tables_2021 available from https://www.gov.uk/government/statistics/hiv-annual-data-tables [accessed 06/01/2022], does not include Wales, Northern Ireland and Scotland. I have multiplied the number seen for care by 1.101 (in 2019 Wales, Northern Ireland and Scotland had a number seen for care which was 0.101 the number seen in England). For 2021 the source is " Table 2b: All people seen for HIV care by probable exposure category" in file "2022-HIV-country-region-data-tables-version-2 -.ods" [accessed 28/12/2022].

f - This information is not available in (39), when available is not specific to GBMSM. For 2018 and 2014 in (34), for 2017 and 2013 in (35), for 2016 (36). Source for 2008-2016: Gill, O.N., et al, The fall in HIV infections in GBMSM in England during 2012 through 2016: When did it begin and what caused it? in IUSTI World and European Congress. 2018. Dublin, Ireland. [Poster]]. This is excluding people who died or who were not linked to HIV care.

g - The source is Table 15 of Key_Population_Tables_2020 available from https://www.gov.uk/government/statistics/hiv-annual-data-tables#history [accessed 04/11/2020] and it refers to GBMSM aged 15+

h - In these data missing are considered at random. The source is Table 6 of National_Tables_2020 available from https://www.gov.uk/government/statistics/hiv-annual-data-tables#history [accessed 04/11/2020] and it refers to GBMSM aged 15+. For 2021 the source is (40).

i - The limits are based for the year 2012-2014 in (41) and for 2015-2018 on (42). The lower limit is based on the actual number of people know to be on PrEP because of PROUD or the PrEP impact trial, the upper limit includes the estimates of GBMSM on private PrEP. The upper limit has been scaled up (using a multiplicative factor of 1.18) to obtain an estimate for the whole UK.

We considered as well estimating the upper limit of the range by calculated the proportion of gay men on PrEP in Scotland, given PrEP is available through the national health service, as we thought that would give us an idea of how many people would be on PrEP if it was for free and available for everyone. In Scotland we estimated there are 48,097 gay and bisexual men (calculated as 0.028 [proportion who reported at least one sexual partner of same sex in the past 5 years among 16-64 in NATSAL] x 1,717,769 (male pop aged 16-64 in Scotland in mid-2016 https://www.nrscotland.gov.uk/statistics-and-data/statistics/statistics-by-theme/population/population-estimates/mid-year-population-estimates/mid-2018).

In Scotland during the first year of implementation (July 2017-July 2018) the NHS PrEP service provided PrEP to 1872 individuals [Source: Health Protection Scotland. Implementation of HIV PrEP in Scotland: First year report., Feb 2019.;], so this gives a % of gay and bisexual men on PrEP of 3.9%.

Using the same approach we estimated that there are 580,132 gay and bisexual men in the UK (calculated as 0.028 x male population age 15-64 in mid 2016 (21,036,300) C:\Users\ValentinaCambiano\Projects\PANTHEON\MSM model\Literature\Census\Figure 2 Age structure of the UK population, mid-2016 and mid-2041]. Therefore we estimated that the maximum number of men on PrEP in 2018 could have been as high as 22,625 (0.039x580,132).

For 2019 we used the information provided by John Sanders by email on the 18th May:

- At end of 2019, there were approx. 16,000 participants enrolled in the Impact Trial. About 95% of these would be GBMSM (this does not include Scotland, Wales and NI), so 15,200
- In Scotland in June 2019 625 prescriptions were issued, 675 in May and 570 in April. Assuming the prescriptions are for 3 months, this would mean that 1870 people were on PrEP. They report that 98% of prescriptions are for GBMSM, so corresponding to 1832.
- The 2019 PrEP user survey (across the UK, 99% of participants are GBMSM) reports that for every 5 trial/programme sourcing PrEP users there are 3 privately sourcing PrEP users (see: O'Halloran et al.)

Based on the estimates above ((16,000*0.95)+(1,870*0.98))/5*8=27,250, which was approximated to 30,000. This does not include Wales and NI.
For 2021, in the 2022 report(40) it is reported that 50,152 GBMSM had initiated or continued PrEP in 2021 in England. This was rounded to 50,200 and multiplied by 1.18 to obtain an estimate for the UK.

j - The lower limit is based on the number of GBMSM tested for HIV in the last year in GUM clinics in England (source 2010(43),2011(44) ,2012-2017(35)). For 2018-2020, these values refers to the number of gay, bisexual and other men who have sex with men who had an HIV test (page 4 (45)) SHS https://assets.publishing.service.gov.uk/government/uploads/system/uploads/attachment_data/file/1037215/hiv-2021-report.pdf. For 2021 the source is (40) where it is reported that 178,466 GBMSM tested in England.

k - The lower limit is based on the number of HIV test performed among GBMSM in GUM clinics in England(41) and for some years it is available the estimate on the number of HIV tests performed in Wales (not specific to GBMSM)(46). For 2019 the estimates are based on the information provided by Nicky Connor on 21/05/2021. She provided information on England: 235,000 tests in GBMSM attending all SHS (192,111 of which were in specialist SHS), 12,532 HIV tests in GBMSM reported from community testing services and 14,050 tests in GBMSM reported from the national HIV self sampling service for a total of 261,582.

## Appendix III. Technical details of the Synthesis Model

### 1 Demography, sexual risk behaviour and transmission of HIV

## 1.1 Demographic model

### 1.1.1 Age distribution and death rates for general population

The model runs for 124 years from 1980, with variables updated in 3 month periods. Three months is an arbitrary choice of time period which allows sufficient sensitivity in capturing changes over time and is also computationally feasible. Each run of the simulation program creates 100,000 simulated people, although only between 25,000 and 28,000 (depending on the category of population growth) are alive and aged 15 or more in 1980. Due to decreasing death rates over time and the general increase in the UK adult population, the GBMSM population size increases over time from 1980-2022 by approximately 27% (See Supplementary Figure 1k).

Age specific death rates for uninfected people (based on UK male death rates in 2008) are as follows:

| Age group | Annual death rate |
|---|---|
| 15-19 | 0.00043 |
| 20-24 | 0.00065 |
| 25-34 | 0.00087 |
| 35-44 | 0.00159 |
| 45-54 | 0.00345 |
| 55-64 | 0.00860 |
| 65-74 | 0.02200 |
| 75-84 | 0.06140 |
| ≥85 | 0.16230 |

These rates are modified as follows to account for the decrease in death rates over time.

Annual death rate at date *d* = Annual death rate x $1.015^{(2008 - d)}$

These death rates are modified by a factor 1.5 for smokers and by 0.75 for non-smokers (see section 2). This is due to the known effects of smoking on all-cause mortality (47). It is assumed 50% of the GBMSM population smokes and that they do not change their habit.

The actual probability of dying in a 3 month period then is estimated as: 1 – *exp* (-0.25*annual death rate)

The initial age distribution is sample between three options of population growth (indicated as inc_cat)

| | Category of population growth (In_cat) | | |
|---|---|---|---|
| | 1 | 2 | 3 |
| Age group [from,to] | Probability of being in age group in 1980* | | |
| [-106,-96] | 0.062 | 0.06 | 0.0651525 |
| [-95,-86] | 0.0617 | 0.05998 | 0.0651525 |
| [-85,-76] | 0.0614 | 0.05996 | 0.0651525 |
| [-75,-66] | 0.0611 | 0.05994 | 0.0651525 |
| [-65,-56] | 0.0608 | 0.05992 | 0.0651525 |
| [-55,-46] | 0.0605 | 0.0599 | 0.0651525 |
| [-45,-36] | 0.0602 | 0.05988 | 0.0651525 |
| [-35,-26] | 0.0599 | 0.05986 | 0.0651525 |
| [-25,-16] | 0.0596 | 0.05984 | 0.05892 |
| [-15,-6] | 0.0593 | 0.05982 | 0.057535 |
| [-5,4] | 0.059 | 0.0598 | 0.05615 |
| [5,14] | 0.0587 | 0.05978 | 0.054765 |
| [15,24] | 0.058393 | 0.059562 | 0.053229026 |
| [25,34] | 0.051922 | 0.052961 | 0.047329911 |

| | | | |
|---|---|---|---|
| [35,44] | 0.043522 | 0.044393 | 0.039673376 |
| [45-54] | 0.040973 | 0.041793 | 0.037349726 |
| [55-64] | 0.038344 | 0.039111 | 0.034952785 |
| [65-74] | 0.029407 | 0.029995 | 0.026806086 |
| [75-84] | 0.011497 | 0.011727 | 0.010480024 |
| [>85] | 0.001742 | 0.001778 | 0.001589 |

* the actual age of a person in a given group in 1980 is determined by sampling from a Uniform distribution

This distribution is chosen to be consistent with the male population of the UK.

Thus around three quarters of simulated people have an age below 15 in 1980. Besides calendar year, the only variable that is modelled and updated up to reaching the age of 15 (when becoming potentially sexually active) is age itself. The "youngest" person in 1980 is age -106 (i.e. will be born in 2086 and reach age 15 in 2101, when the modelling period ends).

## 1.2 Model of sexual risk behaviour and risk of HIV acquisition

Sexual behaviour is characterized by two variables representing, respectively, the number of short term condomless sex partners and whether the man has a current longer term condomless sex partner in the 3 month period. The status of longer term partners is tracked over time (i.e. if they are infected, diagnosed, on ART, etc.). The rationale for explicitly considering short and long term partners is that we know that people have short term and long term partnerships and we wished to track long term partnerships so that the HIV status of the partner (as well as their diagnosis and ART status) could be modelled as this is an important potential source of infection.

### 1.2.1 Determination of number of short term partners at period t

Numbers of short term partners in a given period was generated at random, according to which of four sexual risk behaviour groups the person was in for this period (see also Table 1). Changes in the sexual risk behaviour group from *t-1* to *t* were determined by transition probabilities between 4 groups: no short term condomless partners in 3 month period, 1 short term partner, 2-9 short term partners, and 10 or more short term partners. Transition probabilities $p_{ija}$ of moving from partner group i at *t-1* to partner group j at *t* are given by:

$$p_{ija} = f_{ij} \Bigg/ \left( f_{gi1} + \sum_{j=2-4} (f_{ij} r_a) \right) \qquad for\ j = 1$$

$$p_{ija} = f_{ij} r_a \Bigg/ \left( f_{gi} \ + \sum_{j=2-4} (f_{ij} r_a) \right) \qquad for\ j = 2-4$$

where *a* = 1-10 for age groups 15-, 20-, 25-, 30-, 35-, 40-, 45-, 50-, 55-, 60-, respectively.
Values of $f_{ij}$ and $r_a$ are given in Table 1 and Table 2, respectively.

Values of $r_a$ are modified at time *t* by a factor 0.2 if the subject has a current AIDS defining disease and by a factor *i_ch_risk_diag* for the first six months since the HIV diagnosis, and the square root of *i_ch_risk_diag* afterwards. *i_ch_risk_diag* assumes a value if 0.1 in a certain proportion of the population determined as *1-ch_risk_diag_newp* (distribution beta(3,7)). In addition, there is a person-fixed modification factor. For a random 20% (distribution 0.40 x exp (Normal(0, 0.20) / 2) of men values of $r_a$ are modified by a factor 0.25, and for a further 20% ($\mu_6$ in supplementary Table 3; distribution 0.40 x exp (Normal(0, 0.20) / 2) of men by a factor 0.50, to reflect the fact that a proportion of people experience only very low sexual risk activity throughout their life. Finally, as illustrated in Supplementary Figure 4, we specified changes in relative sexual risk behaviour (compared with that between 1980 and 1985, *crb80*) from 1985 to 1998, with a decline in the 1985 (*crb83*, as was observed (17)), followed by a prolonged period to 1998 of reduced sexual risk behaviour (*crb98*). There is

evidence of significant increases in sexual risk behaviour in the late 1990's(18-22). We therefore parameterized changes in sexual risk behaviour assuming a step increase in 1998 followed by a linear change up to the end of 2021 (the increase per year is indicated by the parameter *beta_r*).

Actual transitions between groups were determined by random sampling. For the first two groups the number of partners in the period is given (i.e. no short term partners, 1 short term partner, respectively). When a person was in the 2-9 short term partners group the number of partners was determined by sampling from a Poisson with mean ($\mu_3$) sampled from the following distribution: 3.5 x exp (Normal(0, 0.20)). When the transition was to ≥ 10 short term partners the number of partners was determined by sampling from a Poisson(2) and multiplied by a factor called *swn* ($\mu_2$ in supplementary Table 3) (distribution: 7 x exp (Normal(0, 0.20)).

### 1.2.2 Determination of having a longer term (condomless) partner at period t

Again, only condomless sex partnerships are modelled. Thus if a person has a longer term partner but condoms are used on all occasions of sexual intercourse then this is not counted as having a longer term partner. In 1980, before any population level reductions in sexual risk behaviour, at each period men with no current longer term condomless sex partner have probability 0.40 of starting having condomless sex with a longer term partner. This can be due to (re-)starting having condomless sex with an existing longer term partner or starting a new partnership which involves condomless sex.

At the time a condomless longer term (CLLT) partnership is started, it is classified into 3 duration groups, each with a different tendency to endure. The percent of people in each group is dependent on age and is shown in Table 3. At time period, *t*, for people with a longer term partner, the probability of the partnership continuing is 0.75 if duration category is 1, is 0.95 if duration category is 2, and 0.98 if duration category is 3. The probability of partnership continuing are increased by the inverse of *ch_risk_diag* (which value was determined during the calibration process) if the subject the long term partner has a relationship with or the long term partner get newly diagnosed with HIV.
To capture the fact that when the HIV epidemic spreads among GBMSM (in the model this is when around 10,000 are living with HIV) the number of long term condomless sex partnerships reduced due to an increase in condom use, it is assumed that, when this happens, in each 3 month period 20% of men with a long term partner interrupt their condomless sex relationship with the long term partner. In addition, at the time a CLLT partnership is formed, it is randomly determined whether a CLLT partner is monogamous (in terms of condomless sex) based on the age specific prevalence of monogamous relationship (no short term partnership) in subjects with a CLLT partner.

The parameter values relating to sexual behaviour are based on comparing model outputs to observed data (13, 18-21).

### 1.2.3 Sexual mixing and determination of number of short term partners who are HIV infected at time t

For each short term partner that a man has at time *t*, the probability that the partner is infected is calculated. This is dependent on the prevalence of HIV in the GBMSM population, taking consideration of age mixing in sexual partnerships. If the subject is of age group *a*, then for each short term partner the first step is to determining by random sampling the age group, *a'*, of the short term partner (for simplicity, all the subject's short term partners during period *t* are assumed to be in this same age group). The age mixing probabilities used to determine this are given by values in Table 4.

Then, for the given partner (of age group *a'*), the risk that the partner is infected is then given by:

$$h_a(t) = \sum_{a'} L^1(t-1) \Big/ \sum_{a'} L(t-1)$$

where $\sum_{a'}$ is the sum over all subjects of age group *a'*, $L^1(t-1)$ is the number of short term partnership formed by people infected with HIV of age group a' at time *t-1*, and $L\,(t-1)$ is the number of short term partnership formed by all people of age group a' at time *t-1*.

Since we assume that all short term partners in period of time *t* are in this same age group, the total number of infected short term partners that the subject has at time $t$, $L^1(t)$, is then given by:

$$L^1(t) = Min\left(Poisson\big(h_a(t)L(t)\big), L(t)\right)$$

### 1.2.4 Determination of probability that a longer term partner is HIV infected at time t

$E^1(t)$ indicates whether the subject has a longer term (condomless sex) partner who is infected ($E^1(t) = 1$ if infected, else $E^1(t) = 0$). A longer term partner at time *t* can be infected either because (i) a new longer term partnership has been formed and the partner was already infected, (ii) because a longer term partner at *t-1*, which has remained a longer term partner at time *t*, has become infected, or (iii) because an infected longer partner has remained as a longer term partner.

In the first scenario (i), where a new condomless sex longer term (CLLT) partnership has been formed: if the CLLT partner is not "monogamous", the probability that he becomes infected with HIV is derived from the HIV incidence at *t-1* for age group a (i.e. the same age group) among those who have a CLLT partnership and at least one short term partner, $i_a(t-1)$.
If the subject is infected with HIV we determine the probability that he is going to transmit HIV to the CLLT at time *t* in the same way as we determine the probability that a CLLT transmit HIV to the subject (see section 1.2.6).

In the second scenario (ii), a CLLT partner at *t-1*, which has remained a CLLT partner at time *t*, has become HIV infected. If this CLLT is monogamous (and we assume this does not change over time), this CLLT partner can be infected only by the subject we track and the probability of transmission is based on the VL, as described in section 1.2.6).
If the CLLT partner is not "monogamous", the probability that he becomes infected is derived from the HIV incidence at *t-1* for age group a (i.e. the same age group) among those who have a CLLT partnership and at least one short term partner, $i_a(t-1)$.
$E^1(t) = 1$ if a sampled random variable from Uniform(0,1) < $i_a(t-1)$, else $E^1(t) = 0$

In order to maintain balance, between the number of uninfected people with a CLLT partner who is HIV infected, and the number of HIV infected people with a CLLT partner who is uninfected, this incidence , $i_a(t-1)$ is modified at time *t* dependent on the degree of balance at time *t-1*.

In the third scenario (iii), where a CLLT relationship with a partner infected with HIV has remained as such.
It is assumed that if a person had a CLLT relationship with a partner HIV infected at time *t-1*, and he is in a CLLT partnership at time *t*, the partner is the same and therefore he is HIV infected.

If $E^1(t-1) = 1$ and $E\,(t) \geq 1$ then we assign $E^1(t) = 1$

### 1.2.5 Determination of the risk of infection from a short term partner / assortativeness

For each HIV infected short term partner his viral load group *v* is obtained by sampling from the viral load distribution of the population of infected men, weighted by the number of partnerships formed. Thus we sample from Uniform(0,1), where the probability of the partner having viral load in group *v* is given by

$$\sum_v L^1(t-1) \Big/ \sum L^1(t-1)$$

where $\sum_v$ is the sum over all HIV-infected subjects in viral load group *v* and $\sum$ is the sum over all HIV-infected subjects.

Viral load groups are:

(1) < 2.7 log cps/mL
(2) 2.7-3.7 log cps/mL
(3) 3.7-4.7 log cps/mL
(4) 4.7-5.7 log cps/mL
(5) $\geq$ 5.7 log cps/mL
(6) primary infection.

Once the viral load group, *v*, of the infected partner is determined, the probability, $t_v$, of the subject being infected by the partner is sampled from the following distributions: $t_1$ = Normal ($\pi_1$,0.000025), $t_2$ = Normal (0.01$\mu_1$,0.0025), $t_3$ = Normal (0.03$\mu_1$,0.0075), $t_4$ = Normal (0.06$\mu_1$,0.015), $t_5$ = Normal (0.1$\mu_1$,0.025), $t_6$ = Normal ($\pi_2$,0.075). $\pi_1$ is sampled from a 0.00001*exp(Normal(0,0.0875), $\pi_2$ is sampled from a 0.2*exp(Normal(0,0.25) and $\mu_1$ is sampled from exp (Normal(0,0.25) (See Supplementary Table 3 for further details). The sampling from the Normal distribution here is to reflect the variable number of sex acts.

These are based on (24-28). These probabilities are increased on average by 2.5-fold if the person has an existing STI (risk of a new STI in any one three month period is given by the number of short term condomless partners / 15 (1 if > 15 short term partners), while the risk of having an STI at time t if the person had a STI at time t-1 is given by the number of short term condomless partners divide by 5) (48).

Realization of whether the subject is infected by each short term partner is determined by sampling from Uniform(0,1).

Choice of who is the partner for a man having a short term partner (which is relevant for sampling the viral load of the partner) is based on the number of partnerships had by others in the population, so if for example one person has 10 short term partners in a 3 month period and another has 1 short term partner then, when sampling to select the viral load and primary infection status of the partnerships being formed in the population in the period, the viral load of the former contributes 10 times the observations to the viral load distribution. In this sense, sexual mixing is assumed to be assortative. We consider it unlikely it could be less assortative than this.

### 1.2.6 Determination of the risk of infection from a longer term partner

Infected longer term partners at time *t* are classified by whether they are in primary infection (if infection occurred at *t-1*), whether they are diagnosed with HIV, whether they are on ART, and whether their current viral load is < 2.7 cps/mL or not.
If a subject had a CLLT living with HIV at time *t-1* , it is assumed it is the same CLLT partner, so if he was diagnosed with HIV, he remains diagnosed at time *t*, if he was on ART it is assumed that in 98% of cases he is still on ART at time *t*, and, if he was suppressed, in 97% of cases he is still suppressed at time *t*.

The proportion of CLLT with HIV who have HIV diagnosed at time *t*, $p_e^D(t)$, is determined with reference to the difference, $d_e^D(t-1)$, in the proportion of subjects with HIV who are diagnosed, $T^D(t-1)/T^1(t-1)$, and $p_e^D(t-1)$ :
$$d_e^D(t-1) = T^D(t-1)/T^1(t-1) - p_e^D(t-1)$$

where $T^D(t-1)$ is the total number of subjects diagnosed with HIV at time *t-1*and $T^1(t-1)$ is the total number of subjects with HIV (diagnosed and undiagnosed) at time *t-1*.
If $d_e^D(t-1) > 0$ then for each subject with a new CLLT partner the probability of the longer term partner being diagnosed is $p_e^D(t)$ with the realization for the individual being determined by sampling from a Uniform (0,1) distribution.

The proportion of those diagnosed who are on ART, and the proportion of those on ART who have viral load < 2.7 log cps/mL are determined in a similar manner. In this way the proportions diagnosed with HIV, on ART, and with current viral load is < 2.7 cps/mL are kept similar for the CLLT as in the simulated subjects themselves.

Risk of infection from a CLLT infected partner at time *t* is determined by probabilities given by sampling from Normal ($\pi_2$ $\mu_5$, 0.075) if the existing partner is in primary infection (i.e. infected at *t-1*), Normal ($\pi_1$ $\mu_5$, 0.000025) if the existing partner has viral load < 2.7 cps/mL, and Normal (0.03, 0.0125) otherwise. The transmission rate is assumed to be higher from CLLT partners than short term partners (due to a higher number of sex acts) by a factor determined by sampling from the distribution 4 x exp (Normal(0, 0.20). $\pi_2$ is sampled from a 0.2*exp(Normal(0,0.25), $\mu_5$ is sampled from 4 x exp (Normal(0,0.25) (See Supplementary Table 3 for further details) and $\pi_1$ is sampled from a 0.00001*exp(Normal(0,0.0875),.

Similarly to the risk of infection from a short term partner, these probabilities are increased on average by 3-fold if the person has an existing STI (see section above regarding prevalence of STI) (48).

### 1.2.7 Infection from partner not resident in the UK

In 50% of simulations it is assumed that individuals in the model can get infected from partners not resident in the UK, regardless of where the sex act happens, but trying to reflect the fact that people resident abroad might have a lower likelihood to be virologically suppressed than those resident in the UK. In these, we sample at a simulation level the probability per 3 months that the CLS partner is not a UK resident [*rate_exp_set_lower_p_vl1000*]. If an individual is having a CLS short term partner who is not resident in the UK, the chance that this part is not virally suppressed is increased by a factor, indicated as *external_exp_factor*. This is to reflect the fact that in 2018 among GBMSM the proportion of PLHIV with VL suppressed was:
88% in the UK, while 75% in Europe (33)

### 1.2.8 Superinfection

Individuals already HIV-positive can be infected again by another individual. This only affects whether an individual acquires transmitted drug resistant virus and therefore subsequently acquires additional resistance mutations. It is assumed that only 20% of those super infected can get transmitted drug resistance, this is based on the fact that the evidence suggests it is not very common that people are observed to gain mutations over time before starting ART.

### 1.2.9 Post-exposure prophylaxis

The use of Post-exposure prophylaxis (PEPSE) is included in the model, mainly to capture the fact that people who receive PrEP and are adherent to it will be less likely to use post-exposure prophylaxis than if they had not been on PrEP. Men are assumed to be eligible for PEPSE if they have at least one short term partner or a long term partner who is not diagnosed with HIV or who is diagnosed with HIV but not virologically suppressed at time *t* and received a test at time *t*. 15% of the GBMSM population is assumed is willing to use PEPSE when the eligibility criteria indicated above are fulfilled, but at each time the probability of initiating PEPSE is 0.2, 0.4 If they have 5 short term partners or more at time *t*.

For men on PrEP, the probability of using PEPSE is 0 if they are adherent to PrEP (>80%) and reduced by 92% otherwise.

### *1.2.10* Pre-Exposure prophylaxis

In the model PrEP is assumed was introduced in 2013, given the PROUD study started at the end of 2012

It is assumed that GBMSM are eligible for PrEP if:

a. they had a negative HIV test at PrEP initiation
b. they had reported condomless anal intercourse in the previous three months (unless the only partner they had condomless sex with was a long-term partner virologically suppressed on ART, due to the low transmission risk (8)),
c. they had an additional documented negative HIV test in the preceding year, very similarly to the PROUD study (49)

These eligibility criteria closely follow the criteria proposed by the PrEP subgroup within the HIV Clinical Reference Group (CRG) and are very similar to those used in the PROUD study (49). The PrEP subgroup prepared a PrEP policy proposition for consideration by the NHS England. Membership of the group included clinicians, academia, public office, public health and HIV advocacy groups.

We assumed a correlation between the probability of starting PrEP per three months and the number of condomless sex partners in the last three months (0·3 if 1 condomless sex partner in the last three months, 0·6 if two to four, 0·7 if five to nine, 0·8 if ten or more) and the presence of rectal STIs (0·8 if currently having an STI or in the last year), so that people with a higher number of partners or presenting with a STI are more likely to initiate PrEP, as observed in demonstration projects(50, 51).

Once a man has satisfied the eligibility criteria and has started PrEP, it is assumed he will use PrEP in any subsequent three month period when having at least one condomless sex partner (unless the only condomless sex partner is a long term partner who is virologically suppressed on ART), unless the person decides to discontinue PrEP or the overall HIV incidence in the GBMSM population drops below 1/10,000 for 5 years. In this latter case it is assumed that the PrEP programme is stopped and is not re-initiated. We assumed that PrEP will be used only in three month periods of condomless sex and that the proportion of days during those three month periods in which they take PrEP is sampled from a distribution with mean 0.76 (95% range: 0.50-0.93).

We assume men on PrEP test for HIV every three months, as recommended by Centers for Disease Control and Prevention for people on PrEP(52) and as recommended by British Association for Sexual Health and HIV for men with such sexual behaviour, regardless of whether they are on PrEP or not(53). This requirement is to minimize the time spent on PrEP in people who have acquired HIV infection despite being on PrEP and therefore reduce the chance of resistance developing. In the eventuality that men become HIV positive they would be diagnosed with HIV at the next test and PrEP is stopped.

On average the effectiveness of PrEP is assumed to be 86%, as found in the PROUD trial, conducted among GBMSM in the UK(49). However, the protection conferred by PrEP is assumed to be the same as the level of adherence to PrEP (measured on a scale from 0 meaning no drugs taken to 1 corresponding to perfect adherence). This assumption is based on the iPrEx OLE findings where no infections were observed at visits where tenofovir diphosphate concentration was 700 fmol per punch or more, consistent with the use of four to seven tablets per week(51). The adherence distribution follows a Beta distribution with parameter alpha=8 and parameter Beta 1.6, so that on average the adherence is 0.83. This adherence distribution corresponds to an average effectiveness of 86%.

### 1.3 Tables and figures referred to in Part 1

**Table 1. Values of $f_{ij}$ (values determining probability of transitioning between short term partner sexual risk behaviour groups)**

| Short term partners in period t-1 | Short term partners in period t | | | |
|---|---|---|---|---|
| | 0 | 1 | 2-9 Poisson mean 3.5* | ≥ 10 Poisson mean 2 x 7* |
| 0 | 0.80 | 0.19 | 0.01 | 0.00 |
| 1 | 0.45 | 0.52 | 0.03 | 0.00003 |
| 2-9 | 0.15 | 0.35 | 0.50 | 0.0001 |
| ≥ 10 | 0.03 | 0.07 | 0.20 | 0.70 |

* x exp (Normal(0, 0.30)

**Table 2. Values of ra (factor determining relative level of sexual risk activity by age)**

| Age group (a=1,10) | $r_a$ |
|---|---|
| 15- | 1.20 |
| 20- | 1.50 |
| 25- | 1.80 |
| 30- | 2.00 |
| 35- | 1.00 |
| 40- | 0.75 |
| 45- | 0.60 |
| 50- | 0.50 |
| 55- | 0.35 |
| 60- | 0.25 |

**Table 3. Percent of newly formed longer term partnerships classified into each of three duration groups, each of which has a different tendency to endure (higher class, more durable)**

| | Duration group | | |
|---|---|---|---|
| Age group | 1 | 2 | 3 |
| 15-44 | 30% | 30% | 40% |
| 45-64 | 30% | 50% | 20% |

**Table 4. The proportion of short term partnerships formed by subjects in age group $a_s$ which are with men of age group $a_p$**

| | Partner Age group ($a_p$) | | | | |
|---|---|---|---|---|---|
| Subject Age group ($a_s$) | 15-24 | 25-34 | 35-44 | 45-54 | 55-64 |
| 15-24 | 0.60 | 0.29 | 0.07 | 0.03 | 0.01 |
| 25-34 | 0.30 | 0.46 | 0.18 | 0.05 | 0.01 |
| 35-44 | 0.10 | 0.30 | 0.38 | 0.20 | 0.02 |
| 45-54 | 0.09 | 0.14 | 0.31 | 0.33 | 0.13 |
| 55-64 | 0.06 | 0.08 | 0.08 | 0.25 | 0.53 |

**Figure 1. Total numbers of short term partners in one period (first quarter of 2000) by age group for subjects (black line) and expected numbers based on age mixing for sexual partners (grey line).**

So, for example, the number of partners of men of age 15-25 matches the number of partners had by men of all ages with men of age 15-25, etc.

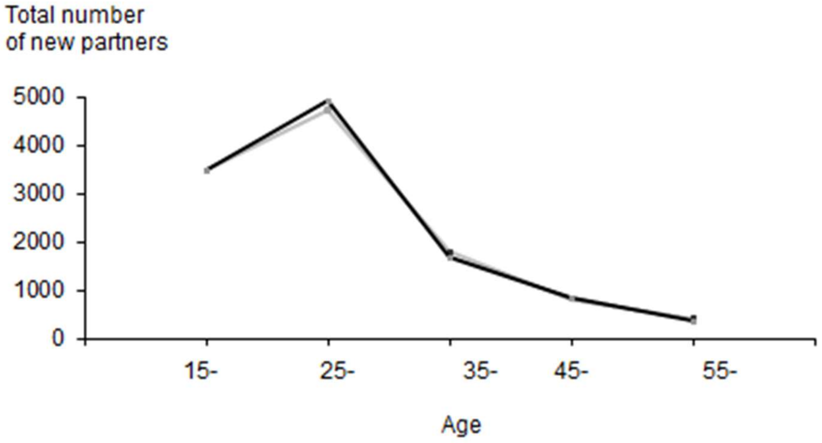

**Figure 2a. Proportion of men age 15-45 with at least one condomless sex partner in the past year by HIV status**

Early in the epidemic those infected are people who generally still within the phase of high risk activity that was the cause of their infection. As the epidemic matures, the average level of risk activity among those infected declines. This is due to the fact that the population of infected people is increasingly made up of people who were infected despite not having high risk activity (e.g. people infected by a longer term partner) and the fact that those who were infected during a period of high risk activity will tend to have reduced levels of sexual activity due to natural variability over time and reductions in risk activity with age.

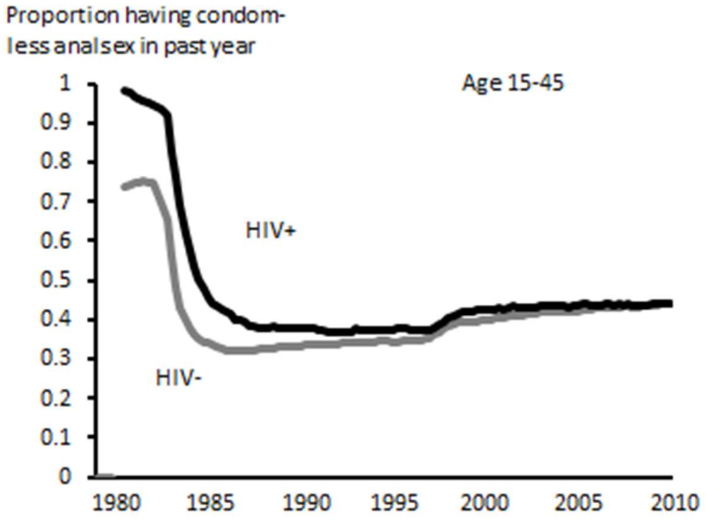


**Figure 2b. Proportion of HIV-infected men (age 15-65) with at least one condomless sex partner in the past year by diagnosis status**

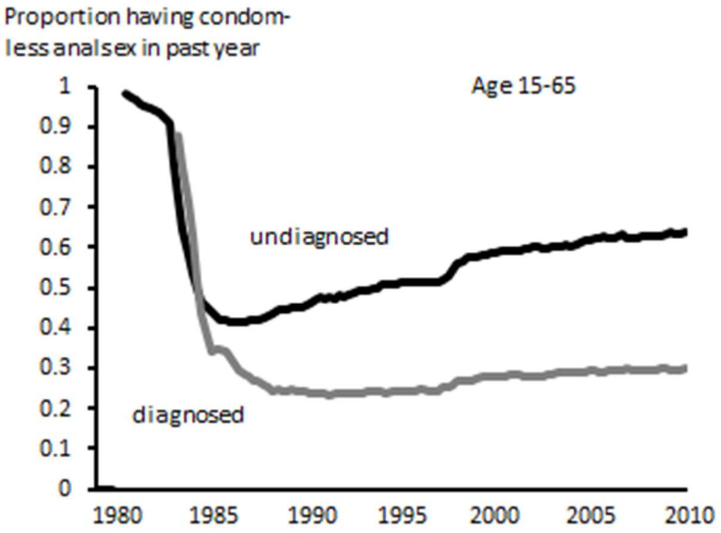

## 2 Untreated HIV infection

## 2.1 Fixed parameters at time of infection

### 2.1.1 Initial viral load

The initial viral load 'set point' (log copies/ml) is distributed as follows (i.e. Normal(4,0.5)):

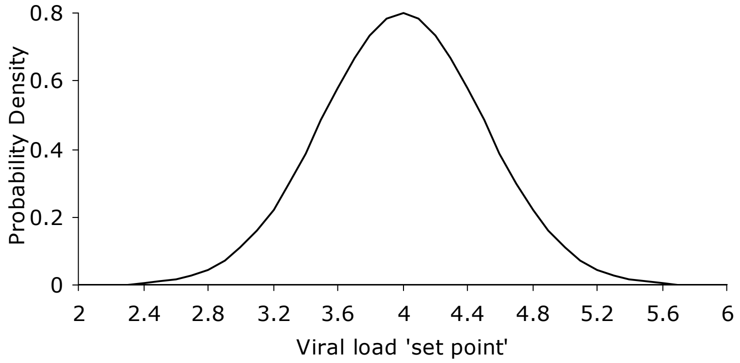


The maximum viral load 'set point' is defined to be 6.5.
The viral load at the time of infection is equal to the initial viral load 'set point'.

### 2.1.2 Initial CD4 count

Initial CD4 count (cells/mm$^3$) is determined on the square root scale. It also depends on the person's viral load 'set point' as follows:
√ CD4 = 32 – (2 x viral load set point) + Normal(0,2)

Or equivalently, the CD4 count (not on square root scale) is approximately distributed as follows:

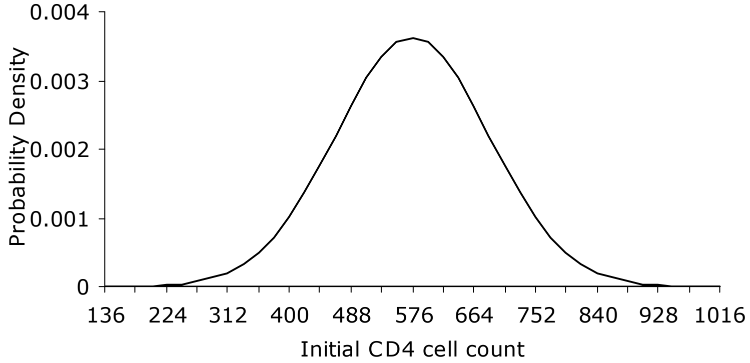


The minimum and maximum initial CD4 count is defined to be 324 and 1500 respectively.

### 2.1.3 Shift to X4 virus

Initial virus is assumed to be R5-tropic.

Shift to presence of X4 virus is assumed to depend on viral load. The probability of shift in a 3-month period is given by $10^v$ x 0.0000004, where v is the most recent viral load, and is thus distributed as follows:

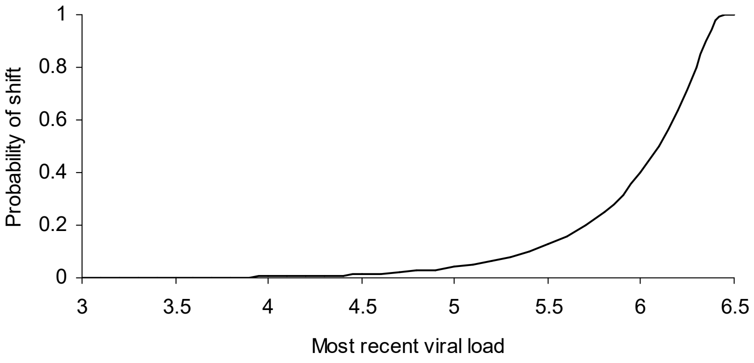


**Comment:** This translates into a rate of 5% per year in a person with viral load 30,000 cps/mL and 16% per year in a person with 100,000 copies/mL, which are broadly consistent with observed data (54).

### 2.1.4 Use of PCP Prophylaxis

In any given 3-month period, there is a 90% probability that a patient will be on PCP prophylaxis given they attend clinic visits and

- Their measured CD4 count is <200 cells/mm$^3$
- or if they have ever been diagnosed with a CDC B symptom or with AIDS

### 2.1.5 Subtypes

Currently in the GBMSM transmission model, the viral subtype is not specified; data to which it is fitted are mainly from Europe and so will reflect the subtypes in circulation – mainly B.

## 2.2 <u>Determination of viral load</u>

### 2.2.1 Changes in viral load

Viral load change (log copies/ml) from period *t-1* to *t* (i.e. in a 3 month period) is given by sampling from a normal distribution with standard deviation 0.05 and mean 0.02275:

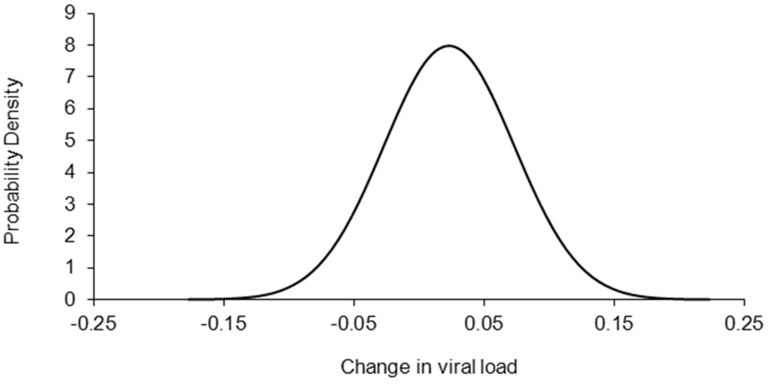


The maximum viral load is defined to be 6.5.

### 2.2.2 Measured viral load

The measured viral load is given by the real viral load plus error (which is sampled from a normal distribution with mean 0 and standard deviation 0.2). This is to reflect the measurement error which arises each time a patient's viral load is measured.

## 2.3 Determination of CD4 count

### 2.3.1 Changes in CD4 count

CD4 change (cells/mm$^3$) from period *t-1* to *t* (i.e. in a 3 month period) is dependent on viral load at *t-1* and is given by sampling from a normal distribution with standard deviation 1.2 and mean as follows:

| Viral load at t-1 | Mean √ CD4 change (per 3 months) |
|---|---|
| < 3 | -0.027 |
| 3.0- | -0.072 |
| 3.5- | -0.135 |
| 4.0- | -0.180 |
| 4.5- | -0.450 |
| 5.0- | -0.900 |
| 5.5- | -1.800 |
| 6.0- | -2.250 |

In addition, the change in CD4 count is affected by current age as follows:

| Age | Additional √ CD4 change (per 3 months) |
|---|---|
| <20 | +0.15 |
| 20- | +0.09 |
| 25- | +0.06 |
| 30- | 0 |
| 35- | 0 |
| 40- | -0.06 |
| 45- | -0.09 |
| 50- | -0.15 |
| 60- | -0.20 |

People with X4 virus present experience an additional change in √ CD4 of -0.25.
The minimum CD4 is defined to be 0.

### 2.3.2 Measured CD4 count

The measured CD4 count is given by the real CD4 plus error (on the square root scale, which is sampled from a normal distribution with mean 0 and standard deviation 2.0). This is to reflect the measurement error which arises each time a patient's CD4 count is measured.

The model also includes the concept of a temporary drop in CD4 count close to the time of infection (to reflect the drop during primary infection), but only for measured CD4 counts and not for the underlying CD4 count as described in section 2.4.1. The drop is distributed ~N(-200,50) and it applies during the six months from infection. Note that this drop in measured CD4 only relates to ART-naïve patients who have been diagnosed and are thus attending clinic (and therefore getting their CD4 counts measured).

The minimum measured CD4 is defined to be 0.

**Comment:** These estimates for viral load and CD4 count in sections 2.2 to 2.4 were selected in conjunction with other relevant parameter values to provide a good fit to the incubation period (to AIDS, death, CD4 thresholds) distribution, and to data on viral load and CD4 count which are given in the model fit. The estimates are derived based on the synthesis of evidence from natural history studies (55-62). Differences that have been found in initial viral load by age are not currently incorporated in the model.

## 2.4 CDC B / AIDS / Death

### 2.4.1 Rate of occurrence of AIDS disease

The rate according to (most recent) CD4 count is as follows:

| CD4 count | Rate (per year) | CD4 count | Rate (per year) |
|---|---|---|---|
| > 650 | 0.002 | 150 - 174 | 0.10 |
| 500 - 649 | 0.010 | 125 - 149 | 0.13 |
| 450 - 499 | 0.013 | 100 - 124 | 0.17 |
| 400 - 449 | 0.016 | 90 - 99 | 0.20 |
| 375 - 399 | 0.020 | 80 - 89 | 0.23 |
| 350 - 374 | 0.022 | 70 - 79 | 0.28 |
| 325 - 349 | 0.025 | 60 - 69 | 0.32 |
| 300 - 324 | 0.030 | 50 - 59 | 0.40 |
| 275 - 299 | 0.037 | 40 - 49 | 0.50 |
| 250 - 274 | 0.045 | 30 - 39 | 0.80 |
| 225 - 249 | 0.055 | 20 -29 | 1.10 |
| 200 - 224 | 0.065 | 10-19 | 1.80 |
| 175 - 199 | 0.080 | < 10 | 2.50 |

### 2.4.2 Rate of occurrence of CDC Category B

The occurrence of CDC category B is taken to be 5-fold higher than the rate for AIDS (as in 2.4.1).

20% of cases of these CDC B category B symptoms are assumed to be tuberculosis (TB).

### 2.4.3 Rate of occurrence of HIV-related deaths

The occurrence of HIV-related deaths is taken to be 4-fold lower than the rate for AIDS (as in section 2.4.1).

**Comment:** The factor 4 was chosen to provide results consistent with observed data, including on the incubation period for death and the time from AIDS to death (in untreated people)(62-65).

#### 2.4.3.1 Cause of deaths

The occurrence of deaths, which are explicitly NOT due to non-HIV causes, is closely related to CD4 count. Some of these deaths however, although related to CD4 count, will not be HIV-related (e.g. other cancers but not including liver deaths as these are modeled separately – see section 2.4.3.2). Therefore, of the CD4-related

deaths, a proportion (15%) will be classified as non-HIV deaths, and the remaining 85% will be classified as HIV-related deaths.

#### 2.4.3.2 Effect of hepatitis co-infection

It is assumed that liver death is closely related to CD4 count.

The rate of death from HIV is divided by 6 to obtain the rate of liver death for those co-infected with hepatitis B or C. These increases in the rate of liver deaths are based on data from observational studies (63, 66-69). Prevalence of HCV and HBV are assumed to be respectively 2% and 3%.

## 2.5 Independent effects on all rates, except death from non-HIV causes (i.e. AIDS / CDC B Symptoms / HIV-related deaths)

### 2.5.1 Independent effect of viral load

Rates are independently affected by (most recent) viral load:

| Viral load (log) | Multiply rate by |
|---|---|
| < 3 | 0.2 |
| 3 - 3.99 | 0.3 |
| 4 - 4.49 | 0.6 |
| 4.5 - 4.99 | 0.9 |
| 5 - 5.49 | 1.2 |
| >= 5.5 | 1.6 |

### 2.5.2 Independent effect of age

Rates increase with age. Multiply rate by $(age/38)^{1.2}$.
For example:

| Age | Multiply rate by |
|---|---|
| 20 | 0.46 |
| 30 | 0.75 |
| 40 | 1.06 |
| 50 | 1.39 |

### 2.5.3 Independent effect of PCP prophylaxis

If on PCP prophylaxis, multiply rates of AIDS and death from HIV (but not rates of CDC B Symptoms) by 0.8.

### 2.5.4 Independent effect of being on ART

The rates depend on the number of drugs in the current regimen:

- Multiply rate by 0.9 if on single drug regimen
- Multiply rate by 0.85 if on 2 drug regimen

- Multiply rate by 0.6 if on 3 drug regimen to reflect that being on HAART has a positive effect on risk of AIDS and death, independent of latest CD4 count and viral load

**Comment:** The estimates for section 2.5 are broadly based on references (70-74).

#### 2.5.5 Independent effect of having TB

When an individual currently has TB or has had TB in the last 3 months, and they have not been diagnosed with AIDS yet, then there is a 50% increased risk of death (but not of AIDS).

#### 2.5.6 Independent effect of having a current ADC

The risk of death (all causes of death, excluding non-HIV causes) increases by 50% if they currently have an ADC.

### 2.6 Rate of occurrence of death from non-HIV causes

Rates from UK national mortality statistics are used (see section 1.1).

#### 2.6.1 Increased risk of death for people with HIV

There is increasing evidence that people with HIV infection itself may have a raised risk of common clinical conditions such as non-AIDS cancers, renal and liver disease and cardiovascular diseases (75-80). Data from observational studies suggest that there is a modest increased risk of death for HIV-positive people with CD4 count greater than 500/mm$^3$, compared to the general population, of the order of approximately 2 (81, 82) but lower for people receiving ART, assumed 1.3 fold higher. Hence, we also assumed that there was a 2-fold increased rate of all non-HIV causes of death throughout life.

#### 2.6.2 Effect of toxicities

Some ART toxicities (See section 5.4) are assumed to increase the chance of deaths:

- Nephrolithiasis by 0.005
- Lactic acidosis by 0.1
- Pancreatitis by 0.1

### 2.7 Summary table of rates (per year)

| Independent effect on rates | | AIDS | CDC B Symptoms | HIV-related deaths | Liver death (for those with HBV or HCV) | Non-HIV deaths |
|---|---|---|---|---|---|---|
| | | Dependent on most recent CD4 count (see 2.4.1) | Rate of AIDS x 5 | Rate of AIDS ÷ 4 | Rate of HIV -related deaths ÷ 6 | Rates from country -specific national mortality statistics x 2 |
| Effect of VL (log/copies) | < 3 | x 0.2 | x 0.2 | x 0.2 | | |
| | 3 - 3.99 | x 0.3 | x 0.3 | x 0.3 | | |
| | 4 - 4.49 | x 0.6 | x 0.6 | x 0.6 | | |
| | 4.5 - 4.99 | x 0.9 | x 0.9 | x 0.9 | | |
| | 5 - 5.49 | x 1.2 | x 1.2 | x 1.2 | | |
| | ≥ 5.5 | x 1.6 | x 1.6 | x 1.6 | | |
| Effect of Age | | x $(age \div 38)^{1.2}$ | x $(age \div 38)^{1.2}$ | x $(age \div 38)^{1.2}$ | | |
| Effect of PCP Prophylaxis | | x 0.8 | | x 0.8 | | |
| Effect of number of drugs in regimen | 1 drug | x 0.9 | x 0.9 | x 0.9 | | |
| | 2 drugs | x 0.85 | x 0.85 | x 0.85 | | |
| | 3 drugs or more | x 0.6 | x 0.6 | x 0.6 | | |
| Effect of having TB currently or in the last 3 months (if not diagnosed yet with AIDS) | | | | x 1.5 | | |
| Effect of having a current ADC | | | | x 1.5 | | |
| Effect of smoking | Smoker | | | | | x 1.5 |
| | Non-smoker | | | | | x 0.75 |

## 2.8 Model fits which are relevant to the natural history

### 2.8.1 Incubation period to AIDS and death from seroconversion (no ART) Observed data from reference(83).

| Year from s/c | % with AIDS | | % died | |
|---|---|---|---|---|
| | Observed | Model | Observed | Model |
| 1 | 0.6 | 0.7 | 0.3 | 0.6 |
| 2 | 2.0 | 2.4 | 1.4 | 1.6 |
| 3 | 4.3 | 5.6 | 3.1 | 3.3 |
| 4 | 8.1 | 10.9 | 5.8 | 6.5 |
| 5 | 13.4 | 17.2 | 9.8 | 11.5 |
| 6 | 19.8 | 23.6 | 14.8 | 17.1 |
| 7 | 25.9 | 30.3 | 20.5 | 23.4 |
| 8 | 32.3 | 37.0 | 27.0 | 30.5 |
| 9 | 38.8 | 43.4 | 33.8 | 38.1 |
| 10 | 46.1 | 49.7 | 40.5 | 45.2 |
| 11 | 53.0 | 55.4 | 48.3 | 51.5 |
| 12 | 58.1 | 60.6 | 55.4 | 57.5 |
| 13 | 63.0 | 66.0 | 62.4 | 63.9 |

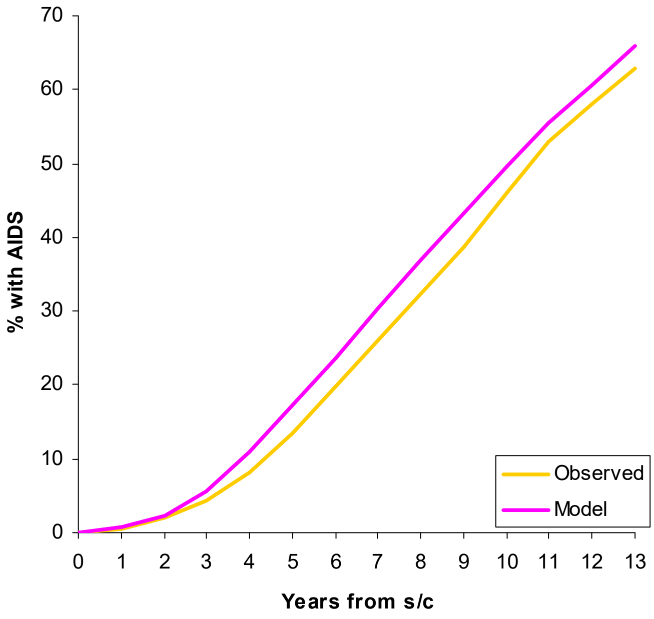


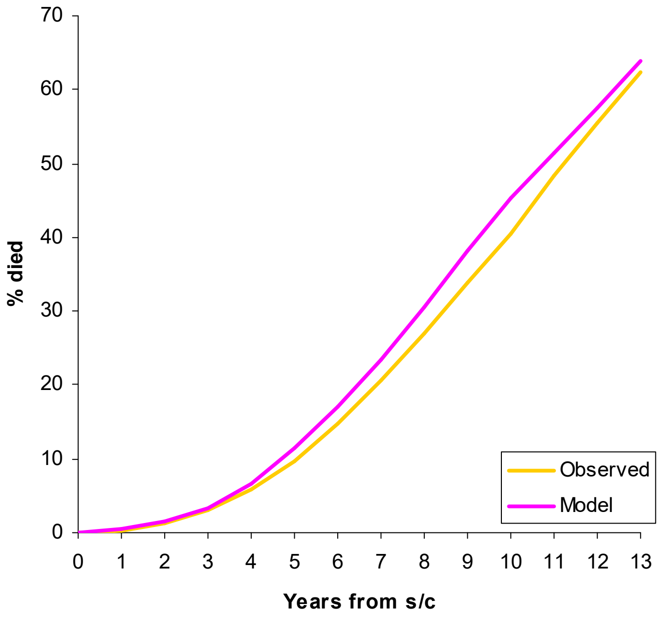

### 2.8.2 Incubation period to CD4 <200, <350, <500 (no ART) Observed data from reference(84).

| Year from s/c | % CD4 < 200 | | % CD4 < 350 | | % CD4 < 500 | |
|---|---|---|---|---|---|---|
| | Observed | Model | Observed | Model | Observed | Model |
| 1 | 8.8 | 2.4 | 26.1 | 16.2 | 48.0 | 47.1 |
| 2 | 12.2 | 10.7 | 33.2 | 32.2 | 55.9 | 61.2 |
| 5 | 32.3 | 38.7 | 55.0 | 60.0 | 72.7 | 78.7 |

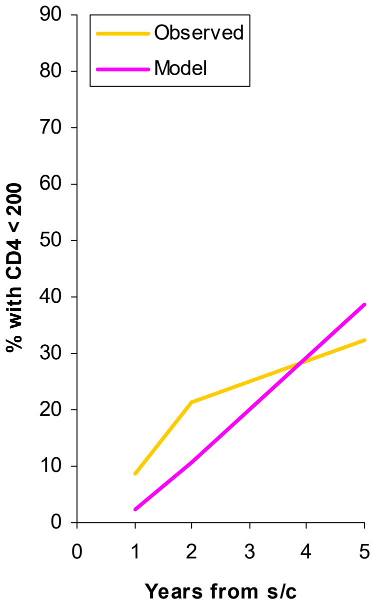


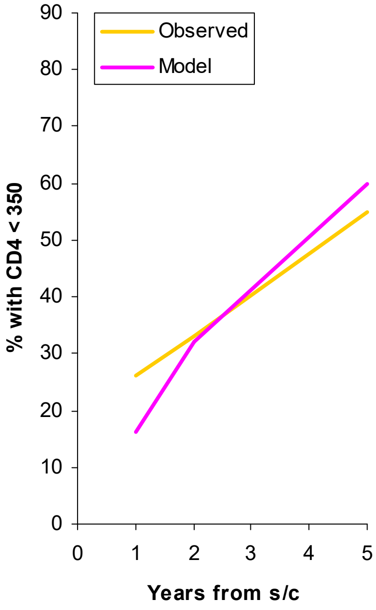


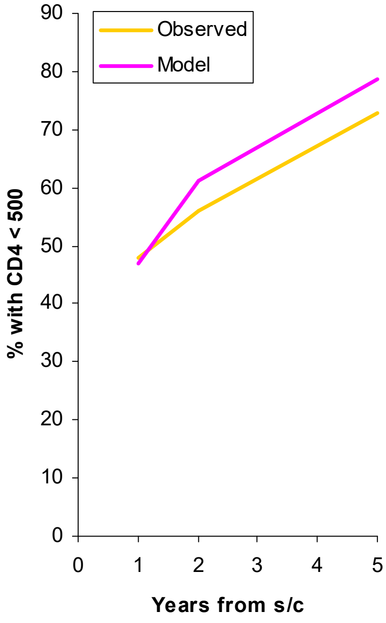


### 2.8.3 Viral load set point and initial CD4 count (after primary infection) Observed data from reference (85)

| | Observed | Model |
|---|---|---|
| Median VL set point: | 4.5 | 4.0 |
| Median CD4: | 570 | 576 |

### 2.8.4 Incubation period AIDS to death (pre-ART era) Observed data from reference (65).

| | % died | |
|---|---|---|
| Years from AIDS diagnosis | observed | Model |
| 1 | 40% | 39% |
| 3 | 84% | 73% |
| Median | 17 months | 18 months |

### 2.8.5 Association between viral load measured close to seroconversion (between 6-24 months) and risk of AIDS, adjusting for CD4 count and age. Observed data from reference (58).

| | Adjusted Relative Hazard Observed (95% confidence interval) | Model |
|---|---|---|
| Viral load (per 0.5 log higher) | 1.87 (1.58 – 2.20) | 2.15 |
| CD4 count (per 100 cells/mm$^3$ lower) | 1.12 (1.02 – 1.24) | 1.14 |
| Age (per 10 years older) | 1.19 (0.96 – 1.47) | 1.28 |

### 2.8.6 Risk of AIDS by CD4 count and viral load and age over 6 years (pre-HAART) Observed data from reference (62).

| | Viral load | Observed | Model |
|---|---|---|---|
| CD4 < 350 | ≤ 1500 - (low n) | | |
| | 1501- 7000 | 19 | 46 |
| | 7001- 20000 | 42 | 59 |
| | 20001- 55000 | 73 | 79 |
| | > 55000 | 92 | 94 |
| CD4 350-500 | ≤ 1500 - (low n) | | |
| | 1501- 7000 | 22 | 18 |
| | 7001- 20000 | 40 | 37 |
| | 20001- 55000 | 57 | 55 |
| | > 55000 | 78 | 74 |
| CD4 > 500 | ≤ 1500 - (low n) | 5 | 5 |
| | 1501- 7000 | 15 | 6 |
| | 7001- 20000 | 26 | 17 |
| | 20001- 55000 | 48 | 28 |
| | > 55000 | 67 | 60 |

* Viral load values used in MACS may need to be multiplied by ~ 2 to approximate to more commonly used Roche assay levels.

### 2.8.7 Median CD4 count at diagnosis of AIDS and at death (pre-HAART era) Observed data from reference(64).

| | AIDS | death |
|---|---|---|
| Observed: | ~ 40 | ~ 0 |
| Model: | 36<br>IQR 10 - 107 | 5<br>IQR 1 - 28 |

# 3 Effect of ART

## 3.1 Adherence

An individual's overall adherence to ART is summarised by the concept of 'effective adherence', which reflects the predicted adequacy of drug levels. Along with other factors, the 'effective adherence' determines the changes in CD4 cell count, viral load and risk of resistance mutation accumulation whilst a patient is on ART (as seen in section 3.3).

The 'effective adherence' depends on a number of components, some of which are fixed for each individual and some of which vary from period-to-period:

'Effective adherence' = Person's underlying tendency to adhere
+ period-to-period variability in person's underlying tendency to adhere
+ effect of calendar year
+ effect of receipt of NNRTI-based regimen
+ effect of experience of an occasional severe drop in adherence

The rationale for including these components and the values uses are described in the sections below.

### 3.1.1 Underlying tendency to adhere and associated period-to-period variability

Each patient has an '*underlying tendency to adhere*' to ART, which is a fixed value for each patient throughout their follow-up.

Each patient also has a '*within person period-to-period variability*' to adhere. The magnitude of this variability is co-determined with the 'underlying tendency to adhere'. Similar to the 'underlying tendency to adhere', the 'within person period-to-period variability' is also a fixed value for each patient throughout their follow-up. The values used for the base model are as shown below.

| Adherence | 'Underlying tendency to adhere' | Probability of given level of 'underlying tendency to adherence' | 'Within person (period-to-period) variability' in adherence (standard deviation) |
|---|---|---|---|
| Low | 0.49 | 3% | 0.2 |
| Moderate | 0.79 | 5% | 0.2 |
| High | 0.9 | 54% | 0.06 |
| Very High | 0.95 | 38% | 0.05 |

A patient's 'underlying tendency to adhere' is restricted to between 0 and 1 inclusive.

**Comment:** These estimates are based partially on observed adherence data(86-91), but also on adherence levels required to produce observed estimates of rates of resistance development and virologic failure (see model fit below) and also data on the proportion of patients at first virologic failure who have no resistance mutations present(92). It is clear from such data in more recent years that the great majority of patients who started ART with three or more drugs are sufficiently adherent that virologic failure rates are low (and so resistance accumulation is also likely to have been slow)(93) .

### 3.1.2 Effect of calendar year

The adherence at time *t* varies from period-to-period. The magnitude of deduction in adherence depends on the current calendar year in the following way:

| Calendar year of infection | Difference in adherence |
|---|---|
| 1996 | -0.12 |
| 1997 | -0.10 |
| 1998 | -0.08 |
| 1999 | -0.07 |
| 2000 | -0.06 |
| 2001 | -0.05 |
| 2002 | -0.04 |
| 2003 | -0.03 |
| 2004 | -0.02 |
| 2005 | -0.01 |
| 2006 | -0.00 |
| 2007 | +0.01 |
| 2008 | +0.02 |
| 2009 | +0.03 |
| 2010 | +0.04 |
| 2011 | +0.04 |
| 2012 | +0.06 |
| 2013 | +0.07 |
| 2014 | +0.08 |
| 2015 | +0.10 |
| 2016 | +0.13 |
| 2017 | +0.16 |
| 2018 | +0.19 |
| 2019 | +0.22 |

Adherence is assumed to have improved over time as the understanding of the need for complete adherence and adherence support has evolved. This is supported by analyses showing improvements over calendar time in observed viral load levels on ART, even for a given specific regimen(93).

### 3.1.3 Effect of receipt of NNRTI-based regimen

If a patient is on an NNRTI-based regimen (i.e. efavirenz or rilpivirine), then the patient's 'effective adherence' increases by 0.1. This effect is included in order to reflect the long half life of NNRTIs(94). This factor is the reason why we refer to the variable as '*effective* adherence'.

### 3.1.4 Effect of experience of an occasional severe drop in adherence

It is assumed that patients on ART are susceptible to occasional severe temporary drops in drug level (effective adherence level) at a rate of 0.02 events per year. On these occasions, the patient's 'effective adherence' declines by 0.6. This leaves them susceptible to viral rebound (but with low risk of resistance, as the effective adherence drop is so profound it would put them in the lowest 'effective adherence' category). This phenomenon is assumed to be 3 times more frequent among those on PI regimens. This latter assumption is what we consider the only plausible means (at least within our model framework) to explain why virologic failure occurring on boosted PI regimens often occurs in the absence of any resistance.

(95-98)

### 3.1.5 Calculation of 'effective adherence'

The 'effective adherence' is calculated for each person at each time point (every 3 months) whilst they are receiving ART.

Once all the components which affect 'effective adherence' (seen in sections 3.1.1 - 3.1.4) have been taken into account, the final value of 'effective adherence' is restricted to between 0 and 1 inclusive.

Example situation

The 'effective adherence' for a person receiving and taking ART in the year 2000, who has a high 'underlying tendency to adhere', and who is on an efavirenz-containing or rilpivirine containing regimen currently, is:

| | |
|---|---|
| 0.9 | [high 'underlying tendency to adhere'] |
| + Normal(0,0.06) | ['Within person (period-to-period) variability'] |
| + (-0.06) | [calendar year = 2000] |
| + 0.1 | [on efavirenz-containing regimen] |

(capped at 0 if this value is < 0 and capped at 1 if this value is > 1).

## 3.2 Use of antiretroviral therapy (ART)

In the GBMSM Transmission model, the following antiretroviral drugs were considered:

| Drug class | Drug name |
|---|---|
| Nucleoside reverse transcriptase inhibitors (NRTIs) | zidovudine |
| | didanosine (ddI) |
| | lamivudine (3tc) |
| | tenofovir |
| Non-nucleoside reverse transcriptase inhibitors (NNRTIs) | efavirenz |
| | rilpivirine |
| Protease Inhibitors (PIs) | indinavir |
| | lopinavir/r |
| | darunavir |
| Integrase Inhibitor | raltegravir |
| | dolutegravir |

**Comment:** These assumptions are based on references(99-102).

## 3.3 Effect of ART

Potent ART regimens are known to reduce viral load, which in turn leads to recovery of CD4 cell counts (49;59;60). Changes in the viral load and CD4 counts are modelled separately when an individual is on ART (whereas in a situation in which the individual is ART-naïve, the CD4 changes are dependent on most recent viral load measurement – see section 2).

### 3.3.1 Determination of viral load and CD4 count changes between t-1 and t

The change in viral load between *t-1* and *t* depends only on the 'effective adherence', the number of active drugs (see section 4 4) and time on the current regimen. The change in CD4 count between *t-1* and *t* depends on the 'effective adherence', the number of active drugs, time on the current regimen, the relative propensity for CD4 rise for that
Risk of development of resistance mutations is also dealt with concomitantly, using the same strata. However, these details are left out of here to make the document more manageable and are considered in section 4.

In the following sections, 'starting current regimen' means starting treatment for the first time as well as any treatment regimen following a treatment interruption.

**Comment:** Changes in viral load and CD4 count are based on observed data and observational studies (and to some extent randomized trials, although responses tend to be better in trial participants), and together with the modelling of development of resistance mutations, provide longer term estimates of virologic failure rates and CD4 count increases in ART. These changes documented here are broadly consistent with observed data (103-108).

### 3.3.1.1 Changes in viral load and CD4 count in first 3 months since starting current regimen.

The initial 3-month change in viral load is described as the mean change from the patient's maximum viral load to that point (*vmax*) on the log scale. This is the mean of a normal distribution with standard deviation 0.2, from which the patient's value/change is sampled.

The change in CD4 count is described as the mean change between periods *t-1* and *t*. This change is then multiplied by a factor which represents each individual's underlying propensity for CD4 count rise whilst on ART (calculation of the factor is described in section 3.3.2.1). If the mean CD4 count change obtained from the table below is positive, then the mean value is subsequently multiplied by this factor.
However, if the CD4 count change in the table is a negative value (i.e. not a CD4 count rise), then it is not multiplied by this factor. Once the mean of the underlying CD4 count is obtained, to obtain the (underlying) CD4 count, variability (SD = 1.2) is added on the square root scale.

| | | Number of active drugs | | | | | | | | | | | |
|---|---|---|---|---|---|---|---|---|---|---|---|---|---|
| | 'Effective adherence' between *t-1* & *t* | 3 | 2.75 | 2.5 | 2.25 | 2 | 1.75 | 1.5 | 1.25 | 1 | 0.75 | 0.5 | 0.25 |
| Viral load (log change from *vmax*) | ≥ 0.8 | -3 | -2.6 | -2.2 | -1.8 | -1.5 | -1.25 | -0.9 | -0.8 | -0.7 | -0.55 | -0.4 | -0.3 |
| | ≥ 0.5, < 0.8 | -2 | -1.6 | -1.2 | -1.1 | -0.9 | -0.8 | -0.6 | -0.5 | -0.4 | -0.25 | -0.1 | -0.05 |
| | < 0.5 | -0.5 | -0.4 | -0.3 | -0.25 | -0.2 | -0.15 | 0 | 0.05 | 0.1 | 0.1 | 0.1 | 0.1 |
| CD4 count change (*t-1* to *t*) | ≥ 0.8 | 180 | 45 | 40 | 35 | 30 | 25 | 20 | 17 | 13 | 10 | 5 | -2 |
| | ≥ 0.5, < 0.8 | 30 | 30 | 23 | 20 | 15 | 13 | 10 | 8 | 5 | 3 | 0 | -7 |
| | < 0.5 | 5 | 4 | 3 | 2 | 1 | -1 | -3 | -6 | -10 | -11 | -12 | -13 |

### 3.3.1.2 Summary of viral load between 3-6 months since starting current regimen and after 6 months if viral load at t-1 > 4 log copies/ml.

This table applies to patients for whom it has been between 3 and 6 months since starting their current regimen, as well as patients who have been on their current regimen for more than 6 months but who have a viral load > 4 log copies/ml (e.g. due to previous poor adherence).

The change in viral load is described as the mean change from the patient's maximum viral load to that point (*vmax*) on the log scale. Otherwise, if the number in the table is underlined, it is the mean absolute value. This is the mean of a normal distribution with standard deviation 0.2, from which the patient's value/change is sampled.

| | | Number of active drugs | | | | | | | | | | | |
|---|---|---|---|---|---|---|---|---|---|---|---|---|---|
| 'Effective adherence' between *t-2* & *t-1* | 'Effective adherence' between *t-1* & *t* | 3 | 2.75 | 2.5 | 2.25 | 2 | 1.75 | 1.5 | 1.25 | 1 | 0.75 | 0.5 | 0.25 |
| ≥ 0.8 | ≥ 0.8 | 0.5 | 0.8 | 1.2 | 1.4 | 2.0 | 2.7 | -1.7 | -1.15 | -0.9 | -0.75 | -0.6 | -0.4 |
| ≥ 0.5, < 0.8 | ≥ 0.8 | 1.2 | 1.2 | 1.2 | 1.4 | -2.0 | -1.6 | -1.2 | -1.05 | -0.9 | -0.7 | -0.5 | -0.35 |
| < 0.5 | ≥ 0.8 | 1.2 | 1.2 | 1.2 | 1.4 | -2.0 | -1.6 | -1.2 | -1.0 | -0.9 | -0.7 | -0.5 | -0.2 |
| ≥ 0.8 | ≥ 0.5, < 0.8 | 1.2 | 1.6 | 1.8 | 2.2 | 2.4 | -2.4 | -1.5 | -0.9 | -0.7 | -0.55 | -0.4 | -0.3 |
| ≥ 0.5, < 0.8 | ≥ 0.5, < 0.8 | 2.5 | 2.5 | 2.5 | 2.5 | -1.2 | -1.1 | -0.8 | -0.65 | -0.5 | -0.35 | -0.2 | -0.05 |
| < 0.5 | ≥ 0.5, < 0.8 | -2.0 | -1.8 | -1.5 | -1.35 | -1.2 | -1.1 | -0.8 | -0.65 | -0.5 | -0.2 | -0.2 | -0.05 |
| ≥ 0.8 | < 0.5 | -0.5 | -0.4 | -0.3 | -0.25 | -0.2 | -0.15 | -0.10 | -0.05 | +0 | +0 | +0 | +0 |
| ≥ 0.5, < 0.8 | < 0.5 | -0.5 | -0.4 | -0.3 | -0.25 | -0.2 | -0.15 | -0.10 | -0.05 | +0 | +0 | +0 | +0 |
| < 0.5 | < 0.5 | -0.5 | -0.4 | -0.3 | -0.25 | -0.2 | -0.15 | -0.10 | -0.05 | +0 | +0 | +0 | +0 |

### 3.3.1.3 Summary of CD4 count change (mean change between t-1 and t) between 3-6 months since starting current regimen and after 6 months if viral load at t-1 > 4 log copies/ml.

This table applies to patients for whom it has been between 3 and 6 months since starting their current regimen, as well as patients who have been on their current regimen for more than 6 months but who have a viral load > 4 log/copies/ml (e.g. due to previous poor adherence).

The change in CD4 count is described as the mean change between periods *t-1* and *t*. This change is then multiplied by a factor which represents each individual's underlying propensity for CD4 count rise whilst on ART (calculation of the factor is described in section 3.3.2.1). If the mean CD4 count change obtained from the table below is positive, then the mean value is subsequently multiplied by this factor.
However, if the CD4 count change in the table is a negative value (i.e. not a CD4 count rise), then it is not multiplied by this factor. Once the mean of the underlying CD4 count is obtained, to obtain the (underlying) CD4 count, variability (SD = 1.2) is added on the square root scale.

| | | Number of active drugs | | | | | | | | | | | |
|---|---|---|---|---|---|---|---|---|---|---|---|---|---|
| 'Effective adherence' between *t-2* & *t-1* | 'Effective adherence' between *t-1* & *t* | 3 | 2.75 | 2.5 | 2.25 | 2 | 1.75 | 1.5 | 1.25 | 1 | 0.75 | 0.5 | 0.25 |
| think≥ 0.8 | ≥ 0.8 | +30 | +28 | +25 | +23 | +21 | +19 | +3 | -5 | -9 | -10.5 | -12 | -14 |
| ≥ 0.5, < 0.8 | ≥ 0.8 | +30 | +28 | +25 | +23 | +7.5 | +1.5 | -4.5 | -7 | -9 | -11 | -13 | -14.5 |
| < 0.5 | ≥ 0.8 | +30 | +28 | +25 | +23 | +7.5 | +1.5 | -4.5 | -7.5 | -9 | -11 | -13 | -16 |
| ≥ 0.8 | ≥ 0.5, < 0.8 | +15 | +13 | +10 | +8 | +7 | +13.5 | +0 | -9 | -11 | -12.5 | -14 | -15 |
| ≥ 0.5, < 0.8 | ≥ 0.5, < 0.8 | +15 | +13 | +10 | +8 | -4.5 | -6 | -10 | -11.5 | -13 | -14.5 | -16 | -17.5 |
| < 0.5 | ≥ 0.5, < 0.8 | +7.5 | +4.5 | +0 | -2 | -4.5 | -6 | -10 | -11.5 | -13 | -16 | -16 | -17.5 |
| ≥ 0.8 | < 0.5 | -13 | -14 | -15 | -15.5 | -16 | -1 | -17 | -17.5 | -18 | -18 | -18 | -18 |
| ≥ 0.5, < 0.8 | < 0.5 | -13 | -14 | -15 | -15.5 | -16 | -16.5 | -17 | -17.5 | -18 | -18 | -18 | -18 |
| < 0.5 | < 0.5 | -13 | -14 | -15 | -15.5 | -16 | -16.5 | -17 | -17.5 | -18 | -18 | -18 | -18 |

### 3.3.1.4 **Summary of viral load (mean change from viral load max), CD4 count change (mean change between t-1 and t), after 6 months, where viral** load at t-1 < 4 log copies/ml.

The change in viral load and CD4 count is as described previously (sections 3.3.1.2 and 3.3.1.3).

| | | Number of active drugs | | | | | | | | | | | |
|---|---|---|---|---|---|---|---|---|---|---|---|---|---|
| | 'Effective adherence' between *t-1* & *t* | 3 | 2.75 | 2.5 | 2.25 | 2 | 1.75 | 1.5 | 1.25 | 1 | 0.75 | 0.5 | 0.25 |
| Viral load (log change from *vmax*) | ≥ 0.8 | 0.5 | 0.9 | 1.2 | 1.4 | -2.5 | -2.0 | -1.4 | -1.15 | -0.9 | -0.75 | -0.6 | -0.3 |
| | ≥ 0.5, < 0.8 | 1.2 | 1.2 | 1.2 | 1.6 | -1.2 | -1.0 | -0.7 | -0.6 | -0.5 | -0.4 | -0.3 | -0.1 |
| | < 0.5 | -0.5 | -0.4 | -0.3 | -0.25 | -0.2 | -0.2 | -0.1 | -0.1 | -0.1 | -0.1 | -0.1 | 0 |
| CD4 count change (*t-1* to *t*) | ≥ 0.8 | +30 | +28 | +25 | +23 | +21 | +19 | +3 | -5 | -9 | -10.5 | -12 | -12 |
| | ≥ 0.5, < 0.8 | +15 | +13 | +10 | +8 | -4.5 | -7.5 | -10 | -12 | -13 | -14 | -15 | -15 |
| | < 0.5 | -13 | -14 | -15 | -15.5 | -16 | -16.5 | -17 | -17 | -18 | -17 | -17 | -17 |

3.3.1.5 <u>Changes in viral load and CD4 count if the number of active drugs in current regimen = 0.</u>

For 0 active drugs, these are the changes regardless of time from start of regimen.

| | | Number of active drugs |
|---|---|---|
| | 'Effective adherence' between *t-1* & *t* | 0 |
| Viral load (log change from *vmax*) | $\geq$ 0.8 | -0.2 |
| | $\geq$ 0.5, < 0.8 | -0.05 |
| | < 0.5 | 0 |
| CD4 count change (*t-1* to *t*) | $\geq$ 0.8 | -15 |
| | $\geq$ 0.5, < 0.8 | -17 |
| | < 0.5 | -18 |

### 3.3.2 Other effects of ART on CD4 count

#### 3.3.2.1 Relative propensity for CD4 count rise

Patients are assumed to vary in their underlying propensity for CD4 count rise whilst on ART. Each person is given a value for their propensity, '*patient_CD4_rise*', which is sampled from exp(Normal(0,0.2)), i.e. log-normal distribution with mean 0 and standard deviation 0.2.

Thus, 'patient_CD4_rise' is distributed approximately as follows:

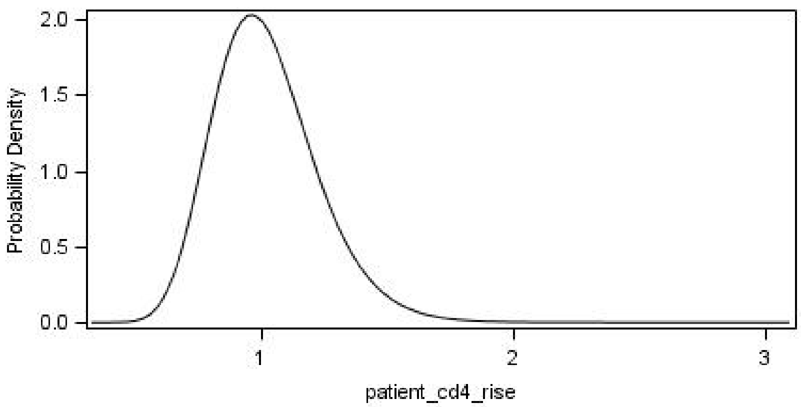


'patient_CD4_rise' is a fixed value that remains constant for the individual over time and is the factor by which the CD4 count change is multiplied by in sections 3.3.1.1 and 3.3.1.3.

If a patient has been on their current regimen for a duration between 1 and 3 years, their underlying propensity for CD4 count rise reduces 1.5-fold and by 2 fold if they have been on their current regimen for more than 3 years, to reflect the fact that the rate of CD4 count increase decreases over time (109, 110).

#### 3.3.2.2 CD4 decline with failing NNRTI or II-regimen

When a patient is on an NNRTI-regimen (i.e. efavirenz or rilpivirine) but not on a PI (i.e. lopinavir, darunavir, indinavir), and they are failing the regimen, then the CD4 depletion is greater (by -6 cells/mm$^3$) in any given 3-month period. Failure of the regimen is defined in this situation by a number of active drugs (concept explained under 4.3.8) being less than or equal to 2.
The same happens in the same situation if a patient is on a II-regimen (i.e. dolutegravir or raltegravir), rather than NNRTI-regimen but not on a PI.

#### 3.3.2.3 CD4 adjustment related to age

The change in CD4 between *t-1* and *t* depends on age, in particular younger people tend to recover CD4 cells quicker than older people. It is calculated using the following formula:

$$[age(t) - 40] * -0.3$$

So it will assume an additional value of 7.5 for people age 15 and a value of -7.2 for people aged 64 to the value calculated in section 3.3.1.

#### 3.3.2.4 CD4 adjustment related to receiving protease inhibitors

People receiving protease inhibitors (IND, LPR or DAR) are assumed to have a quicker CD4 recovery, a value of 3 is added to the CD4 count change calculated in section 3.3.1.

#### 3.3.2.5 CD4 adjustment related to age

Finally women are assumed to have a quicker CD4 recovery than men, a value of 2 is added to the CD4 count change calculated in section 3.3.1.

#### 3.3.2.6 Variability in CD4 count whilst on ART

When a person is ART-naïve, the CD4 count change from period *t-1* to *t* (i.e. in a 3 month period) is given by sampling from a normal distribution with mean dependent on viral load at *t-1*, and standard deviation 1.2 on the square root scale (see section 2.3).

When a person is on ART, the same magnitude of variability (i.e. standard deviation of 1.2 on the square root scale) as for when the person is ART-naïve, is given to the CD4 count change. (This variability has already been described above in sections 3.3.1.1 and 3.3.1.3)

**Comment:** Note that this is not the same as the variability given to *measured* CD4 count (which has a standard deviation of 2.0 on the square root scale, as found in section 2.2.2), which applies to people both on and off ART.

#### 3.3.2.7 Maximum CD4 count

After all the effects of ART (as seen above) have been taken into account to calculate the CD4 count, if the CD4 count is greater than the maximum CD4 count assigned for that person, then a further expression is used to calculate the resulting CD4 count at time *t*:
Final CD4 at time *t* = (Maximum CD4 count) + Normal(0,50)

#### 3.3.2.8 Minimum CD4 count

As mentioned in section 2.3, the minimum CD4 count and minimum measured CD4 count are both defined to be 0.

#### 3.3.2.9 Calculation of measured CD4 count (whilst on ART)

The measured CD4 count is calculated for each person at every time point (3 months). The example below applies only to individuals who are taking ART.

Once all the components which affect measured CD4 count (seen in sections 3.3.1-3.3.2.6) have been taken into account, the final value of measured CD4 count is restricted to between 0 and the maximum CD4 count as calculated in section 3.3.2.7.

Example situation

The measured CD4 count for a person at time *t*, given that their actual CD4 count and viral load at *t-1* was 350 cells/$mm^3$ and 4.1 log copies/ml respectively, and they have been on their current regimen for over 6 months, where the number of active drugs in their regimen totals 2.50, and given that their ‘effective adherence’ has been consistently over 0.8, and their underlying propensity for CD4 count rise (patient_cd4_rise) = 1.1, is:

$$\left\{\sqrt[2]{\sqrt[2]{[350 + (25x1.1)] + Normal(0,1.2)}^{2} + Normal(0,2.0)}\right\}^{2}$$

where +25 is the CD4 change obtained from section 3.3.1.3, Normal(0,1.2) is the variability given to the change in CD4 whilst on ART, and Normal(0,2.0) is the variability given for *measured* CD4 count.

(capped at 0 if this value is < 0 and capped at (Maximum CD4 count) + Normal(0,50) if this value is > assigned maximum CD4 for that person).

## 3.4 Model fits which are relevant to the effect of ART

### 3.4.1 Mean changes from baseline in CD4 cell count and viral load whilst on zidovudine mono-therapy (patients with no previous treatment) Observed data from references(111, 112)

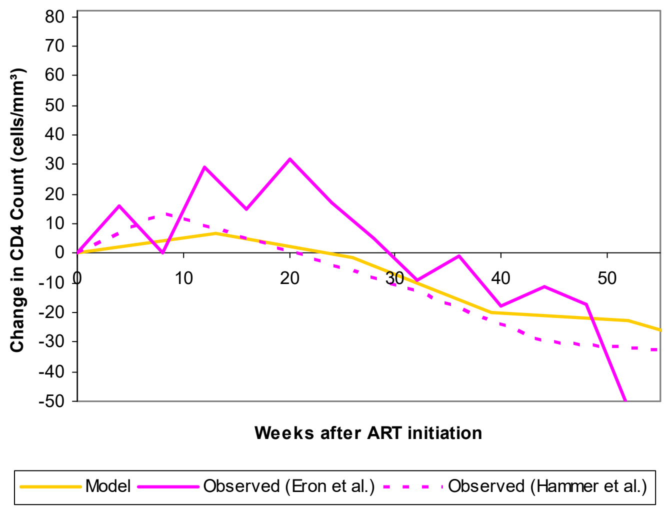


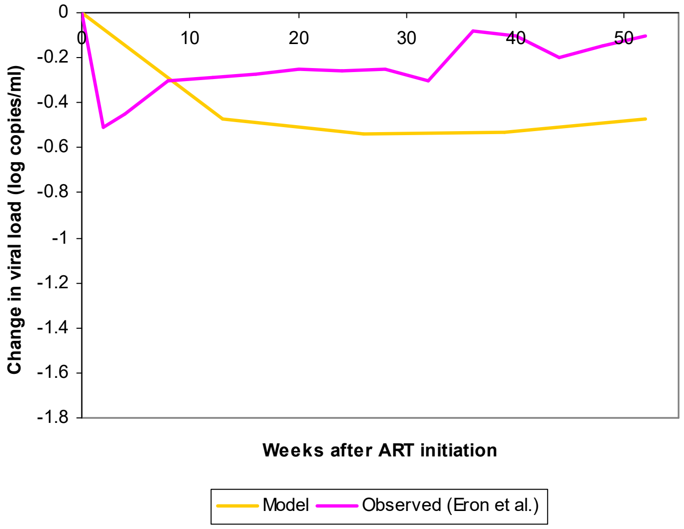


### 3.4.2 Mean changes from baseline in CD4 cell count and viral load whilst on zidovudine/3TC dual-therapy (patients with no previous treatment) Observed data from reference (111)

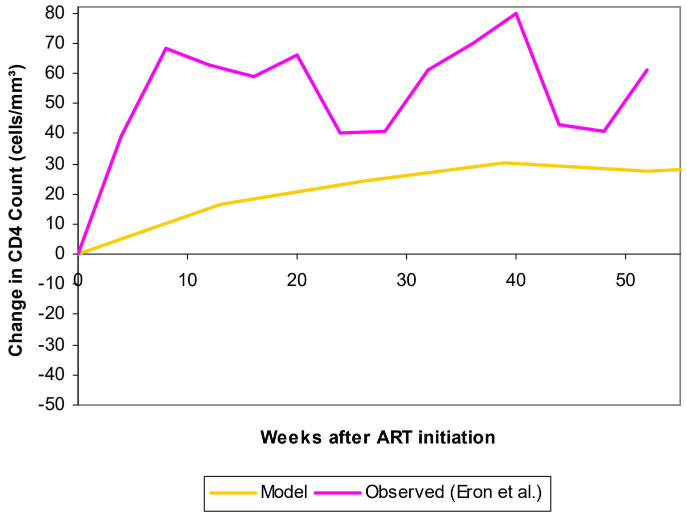


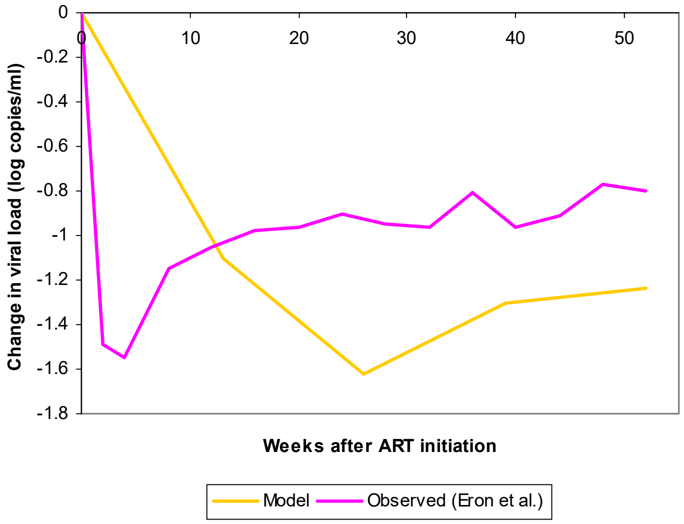

### 3.4.3 3 year percent risk of AIDS after start of ART by baseline CD4 / viral load (age < 50, non-IDU, AIDS-free)

**Observed data from reference(113).**

| Baseline viral load | Baseline CD4 count | Observed | Model |
|---|---|---|---|
| < 100,000 | < 50 | 16% | 23% |
| | 50-99 | 12% | 15% |
| | 100-199 | 9% | 8% |
| | 200-349 | 5% | 6% |
| | ≥ 350 | 3% | 2% |
| ≥ 100,000 | < 50 | 20% | 18% |
| | 50-99 | 16% | 16% |
| | 100-199 | 12% | 4% |
| | 200-349 | 6% | 4% |
| | ≥ 350 | 4% | 0% |

### 3.4.4 Effect of HAART vs no therapy on risk of AIDS and death

**Observed data from reference(114).**

Simulated trial with 5 years follow-up.
Relative hazard of AIDS, (HAART vs no therapy)

| Observed | Model |
|---|---|
| 0.1 | 0.16 |

### 3.4.5 % with virologic failure (viral load > 500 copies/mL, whilst on ART) by time from start of HAART (patients starting with PI/r or NNRTI regimen)

**Observed data from reference(115).**

| Years from start of HAART | Observed | Model |
|---|---|---|
| 1 | 7% | 10% |
| 2 | 13% | 16% |
| 3 | 17% | 19% |
| 4 | 20% | 23% |
| 5 | 22% | 26% |
| 6 | 24% | 28% |
| 7 | 27% | 30% |
| 8 | 29% | 32% |

Observed data may be overestimates due to some unrecognised stopping of ART.

### 3.4.6 Rate of viral rebound in people on 1st line HAART and with viral load < 50 copies/mL

**Observed data from reference(116).**

| | Rate per 100 person -years |
|---|---|
| Observed | 3-6 |
| Model | 5.5 |

### 3.4.7 Median CD4 count change (cells/mm3) at 3 years from start of HAART Observed data from reference(103).

| Observed | Model |
|---|---|
| 273 | **274** |

### 3.4.8 Percent with triple class virologic failure by years from start of HAART (patients naïve before HAART). Observed data from reference(117).

Modelled estimates based on ART start years 1998-2008 inclusive.

| Years from start of HAART | Observed | Model |
|---|---|---|
| 5 | 3.4% | **6.8%** |
| 9 | 8.6% | **12.6%** |

# 4 HIV drug resistance

## 4.1 Resistance mutations; introduction

### 4.1.1 Resistance mutations included in the model

The resistance mutations considered in the model are as follows. We do not specify the mutated amino acid for each position; it is assumed that for a given codon position, the mutations considered are those that confer resistance (e.g. for M184 this is I or V). The exception to this are the mutations at codon 50 of protease inhibitors – we may need to review other mutations to see if they need to be split, but there is a cost to adding any new variables.

The choice of mutations to include reflects a balance between the desire to capture important specific effects and the need to limit the complexity of the model and the number of variables simulated. The IAS-USA resistance guidelines provided the basis for choice of mutations(118).

| Drug class | Resistance mutations | Notes |
|---|---|---|
| NRTI | M184 | |
| | TAMS | TAMS (thymidine analog mutations) are modelled such that it is the number of TAMS which affect the drug activity, rather than the specific mutations itself |
| | K65 | |
| | L74 | |
| | Q151 | |
| NNRTI | nn | A specific resistance mutation (or mutations) which confers resistance to an NNRTI drug |
| PI | D30 | |
| | V32 | |
| | L33 | |
| | M46 | |
| | I47 | |
| | I50V | |
| | I50L | |
| | I54 | |
| | L76 | |
| | V82 | |
| | I84 | |
| | L90 | |
| Integrase Inhibitor | primary | |
| | secondary | |

**Comment:** Note that the possibility of mutations to anticipated drugs is accounted for. This is necessarily crude (as the new drugs that will be licensed and their resistance profiles are as yet uncertain) but conveys the fact that new drugs are under development for which the virus will have to develop new mutations to evade. However, it is clear that we are now in a position to be more specific about mutations to some new drugs, such as raltegravir, rilpivirine and dolutegravir, and therefore update the model.

### 4.1.2 Types of variables used to model resistance mutations

Resistance mutations can be present in majority or minority virus and this is also reflected in the model. In the following sections, *resistancemutation* can represent any of the mutations as listed in the table in section 4.1.1:

c_*resistancemutation* = 1 if mutation is present in majority virus
= 0 if not

e_*resistancemutation* = 1 if virus with mutation is present at all
= 0 if not

Once e_*resistancemutation* takes the value 1, it can never revert to 0.

## 4.2 ART-naïve patients

### 4.2.1 Transmitted drug resistance

There is a possibility of resistance mutations being present in the acquired virus (transmitted drug resistance) at the point of infection.

The viral load group of the man who infected the subject is known, as indicated above (for infection from a short term partner the viral load group of the 6 groups defined in section 1.2.5 is known, while if infected by a longer term partner the viral load is known to be either < 2.7, $\geq$ 2.7 but not primary infection, or primary infection, as described in section 1.2.6). For a subject infected by a person in viral load group v the probability of a resistance mutation being present in the infected person is given by

$$\sum_{v,r=1} L^1(t-1) \Big/ \sum_{v} L^1(t-1)$$

Where $\sum_{v,r=1}$ is the sum over all HIV-infected subjects in viral load group *v* for whom a resistance mutation is present in majority virus and $\sum_{v}$ is the sum over all HIV-infected subjects in viral load group *v*. Again, realization of whether the subject is infected by a person with at least one resistance mutation in majority virus is determined by sampling from Uniform(0,1).

For subjects infected from a source partner with a resistance mutation, the probability that a specific mutation, m, is present in the source is given by

$$\sum_{r=1,m=1} L^1(t-1) \Big/ \sum_{r=1} L^1(t-1)$$

where $\sum_{r=1,m=1}$ is the sum over all HIV-infected subjects with mutation *m* present in majority virus and $\sum_{r=1}$ is the sum over all HIV-infected subjects with at least one resistance mutation in majority virus.

If a given resistance mutation *m* is present in the source partner, the probability that the mutation is both transmitted and survives in the subject (i.e. that its presence will affect future response to drugs for which the mutation confers reduced sensitivity) is mutation specific, as shown in the table below:

| Resistance mutation | Average probability the mutation is both transmitted and survives in the subject* |
|---|---|
| M184 | 0.20 |
| K65 | 0.20 |
| L74 | 0.5 |
| Q151 | 0.5 |
| Thymidine analogue mutations (TAMS) | 0.5 |
| NNRTI mutation | 0.8 |
| PI mutations | 0.5 |
| II primary mutations | 0.80 |
| II secondary mutations | 0.80 |

*Except for M184V and K65R, for the other mutations these probabilities are multiplied by the factor res_trans_factor

**Comments:** Probabilities based on evidence from studies comparing distribution of resistance mutations between treated and antiretroviral naïve populations; e.g. refs(119, 120).

### 4.2.2 Loss of transmitted drug resistance mutations from majority virus

The table below indicates the probability per 3 months of losing from majority virus a specific mutation in people who were infected with that resistance mutations while ART-naïve.

| Resistance mutation | Probability the mutation is lost per 3 month period |
|---|---|
| M184 | 0.04 |
| K65 | 0.04 |
| L74 | 0.04 |
| Q151 | 0.04 |
| Thymidine analogue mutations (TAMS) | 0.04 (the # of TAMS is reduced by 1)_ |
| NNRTI mutation | 0.04 |
| PI mutations | 0.04 |
| II primary mutation | 0.04 |
| II secondary mutations | 0.04 |

Note that if a person was infected with virus with a given mutation, then this mutation is never completely lost.

## 4.3 Patients on ART

### 4.3.1 Abbreviations of antiretroviral drugs used in the model

The list of drugs and their abbreviations are included in 3.2.

### 4.3.2 Determination of acquisition of new resistance mutations between *t-1* and *t*: Stage 1

For people on ART there is a certain risk of development of resistance mutations, which is determined by the number of active drugs in the regimen, viral load, the effective adherence (see section 3.1) and the time since starting (or re-starting after interruption) the current period of continuous therapy.

The probability of acquiring new resistance mutations in a given 3 month period in people on ART (i.e. between *t-1* and *t*) is determined in a series of stages. The first stage is to determine the '*new mutation factor*'. The magnitude of this factor reflects the risk of resistance mutations emerging, but it cannot in itself be interpreted as the risk of resistance emerging. The process for generating these values is described on the following pages. The changes in viral load and CD4 count are also dealt with concomitantly, using the same strata. See section 3.3 for details.

#### 4.3.2.1 ‘New mutation factor’ in first 3 months since starting current period of continuous therapy

The ‘new mutation factor’ is given in the table below.

| ‘Effective adherence’ between *t-1* & *t* | Number of active drugs | | | | | | | | | | | |
|---|---|---|---|---|---|---|---|---|---|---|---|---|
| | 3 | 2.75 | 2.5 | 2.25 | 2 | 1.75 | 1.5 | 1.25 | 1 | 0.75 | 0.5 | 0.25 |
| ≥ 0.8 | 0.002 | 0.01 | 0.03 | 0.05 | 0.1 | 0.15 | 0.2 | 0.3 | 0.4 | 0.45 | 0.5 | 0.5 |
| ≥ 0.5, < 0.8 | 0.15 | 0.15 | 0.2 | 0.25 | 0.3 | 0.3 | 0.3 | 0.35 | 0.4 | 0.45 | 0.5 | 0.5 |
| < 0.5 | 0.05 | 0.05 | 0.05 | 0.05 | 0.05 | 0.05 | 0.05 | 0.05 | 0.05 | 0.05 | 0.05 | 0.05 |

#### 4.3.2.2 Summary of ‘new mutation factor’ between 3-6 months since starting current period of continuous therapy and after 6 months if viral load at t-1 > 4 log copies/ml

This table applies to patients for whom it has been between 3 and 6 months since starting their current period of continuous therapy, as well as for patients whom it has been more than 6 months since their current period of continuous therapy but who have a high viral load (e.g. due to previous poor adherence). The numbers given in the table below correspond to the ‘new mutation factor’.

| ‘Effective adherence’ between *t-2* & *t-1* | ‘Effective adherence’ between *t-1* & *t* | Number of active drugs | | | | | | | | | | | |
|---|---|---|---|---|---|---|---|---|---|---|---|---|---|
| | | 3 | 2.75 | 2.5 | 2.25 | 2 | 1.75 | 1.5 | 1.25 | 1 | 0.75 | 0.5 | 0.25 |
| ≥ 0.8 | ≥ 0.8 | 0.002 | 0.01 | 0.03 | 0.05 | 0.05 | 0.1 | 0.2 | 0.3 | 0.4 | 0.45 | 0.5 | 0.5 |
| ≥ 0.5, < 0.8 | ≥ 0.8 | 0.002 | 0.01 | 0.03 | 0.05 | 0.05 | 0.1 | 0.2 | 0.3 | 0.4 | 0.45 | 0.5 | 0.5 |
| <0.5 | ≥ 0.8 | 0.05 | 0.05 | 0.03 | 0.05 | 0.05 | 0.1 | 0.2 | 0.3 | 0.4 | 0.45 | 0.5 | 0.25 |
| ≥ 0.8 | ≥ 0.5, < 0.8 | 0.10 | 0.15 | 0.2 | 0.2 | 0.3 | 0.3 | 0.3 | 0.35 | 0.4 | 0.45 | 0.5 | 0.5 |
| ≥ 0.5, < 0.8 | ≥ 0.5, < 0.8 | 0.10 | 0.15 | 0.2 | 0.2 | 0.3 | 0.3 | 0.3 | 0.35 | 0.4 | 0.45 | 0.5 | 0.5 |
| < 0.5 | ≥ 0.5, < 0.8 | 0.10 | 0.15 | 0.2 | 0.2 | 0.3 | 0.3 | 0.3 | 0.35 | 0.4 | 0.45 | 0.5 | 0.25 |
| ≥ 0.8 | < 0.5 | 0.05 | 0.05 | 0.05 | 0.05 | 0.05 | 0.05 | 0.05 | 0.05 | 0.05 | 0.05 | 0.05 | 0.05 |
| ≥ 0.5, < 0.8 | < 0.5 | 0.05 | 0.05 | 0.05 | 0.05 | 0.05 | 0.05 | 0.05 | 0.05 | 0.05 | 0.05 | 0.05 | 0.05 |
| < 0.5 | < 0.5 | 0.05 | 0.05 | 0.05 | 0.05 | 0.05 | 0.05 | 0.05 | 0.05 | 0.05 | 0.05 | 0.05 | 0.05 |

4.3.2.3 ‘New mutation factor’ after 6 months since starting current period of continuous therapy, where viral load at t-1 < 4 log copies/ml.

The ‘new mutation factor’ is given in the table below.

| | Number of active drugs | | | | | | | | | | | |
|---|---|---|---|---|---|---|---|---|---|---|---|---|
| ‘Effective adherence’ between *t-1* & *t* | 3 | 2.75 | 2.5 | 2.25 | 2 | 1.75 | 1.5 | 1.25 | 1 | 0.75 | 0.5 | 0.25 |
| $\geq$ 0.8 | 0.002 | 0.01 | 0.03 | 0.08 | 0.1 | 0.15 | 0.2 | 0.3 | 0.4 | 0.45 | 0.5 | 0.5 |
| $\geq$ 0.5, < 0.8 | 0.15 | 0.18 | 0.2 | 0.25 | 0.3 | 0.3 | 0.3 | 0.35 | 0.4 | 0.45 | 0.5 | 0.5 |
| < 0.5 | 0.05 | 0.05 | 0.05 | 0.05 | 0.05 | 0.05 | 0.05 | 0.05 | 0.05 | 0.05 | 0.05 | 0.05 |

**4.3.3**

4.3.3.1 ‘New mutation factor’ if the number of active drugs in current regimen = 0.

For 0 active drugs, these numbers below are the ‘new mutation factor’ regardless of time from start of current period of continuous therapy.

| | Number of active drugs |
|---|---|
| ‘Effective adherence’ between *t-1* & *t* | 0 |
| $\geq$ 0.8 | 0.5 |
| $\geq$ 0.5, < 0.8 | 0.5 |
| < 0.5 | 0.05 |

#### 4.3.3.2 ‘New mutation factor’ when on efavirenz or rilpivirine in particular circumstances

The ‘new mutation factor’ is given in the table below.

| | | | Number of active drugs | | | | | | | | | | | | |
|---|---|---|---|---|---|---|---|---|---|---|---|---|---|---|---|
| Time since starting current period of continuous therapy | ‘Effective adherence’ between *t-2* & t | ‘Effective adherence’ between *t-1* & *t* | 3 | 2.75 | 2.5 | 2.25 | 2 | 1.75 | 1.5 | 1.25 | 1 | 0.75 | 0.5 | 0.25 | 0 |
| First 3 months | na | < 0.5 | 0.15 | 0.15 | 0.2 | 0.25 | 0.3 | 0.3 | 0.3 | 0.3 | 0.4 | 0.45 | 0.5 | 0.5 | 0.5 |
| 3-6 months* | ≥ 0.8 | < 0.5 | 0.1 | 0.15 | 0.2 | 0.2 | 0.3 | 0.3 | 0.3 | 0.35 | 0.4 | 0.45 | 0.5 | 0.5 | Na |
| | ≥ 0.5, < 0.8 | < 0.5 | 0.1 | 0.15 | 0.2 | 0.2 | 0.3 | 0.3 | 0.3 | 0.35 | 0.4 | 0.45 | 0.5 | 0.5 | Na |
| | < 0.5 | < 0.5 | 0.1 | 0.15 | 0.2 | 0.2 | 0.3 | 0.3 | 0.3 | 0.35 | 0.4 | 0.45 | 0.5 | 0.5 | Na |
| After 6 months** | na | < 0.5 | 0.15 | 0.18 | 0.2 | 0.25 | 0.3 | 0.3 | 0.3 | 0.35 | 0.4 | 0.45 | 0.5 | 0.5 | na |

*and after 6 months if viral load at t-1 > 4 log copies/ml; ** where viral load at t-1 < 4 log copies/ml; na: not applicable;

### 4.3.4 Determination of acquisition of new resistance mutations between *t-1* and *t*: Stage 2

The second stage is for the 'new mutation factor' to be multiplied by the log viral load (mean of log viral load at *t-1* and *t*).

This is then converted to a number that can be used as a probability, by truncating at 1.

### 4.3.5 Determination of acquisition of new resistance mutations between *t-1* and *t*: Stage 3

The value determined from Stage 2 (section 4.3.4) is used as a probability to determine whether the third stage is entered. If this chance arises in a given 3 month period, which is determined by sampling from the binomial distribution, then the following criteria operate:

#### 4.3.5.1 NRTIs

| Resistance mutation | Probability of arising | Conditions |
|---|---|---|
| M184 | 80% | if (on 3TC) |
| # TAMS increases by 1 | 20% | if (on ZDV) and (not on 3TC) |
| | 12% | if (on ZDV) and (on 3TC) |
| # TAMS increases by 2 | 1% | if (on ZDV) and (not on 3TC) |
| | 1% | if (on ZDV) and (on 3TC) |
| K65 | 2% | if (on TDF or DDI) and (on ZDV) |
| | 10% | If (on TDF or DDI) and (not on ZDV) |
| L74 | 1% | if (on DDI) |
| Q151 | 2% | if (on DDI or ZDV) |

#### 4.3.5.2 NNRTIs

| Resistance mutation | Probability of arising | Conditions |
|---|---|---|
| nn | 80% | if (on EFA or RPV) |

#### 4.3.5.3 PIs

We assume a different probability of resistance mutation accumulation depending on whether the PI would be boosted or not (which for simplicity, we assume it depends entirely on the current calendar year).

| Resistance mutation | Probability of arising | Conditions |
|---|---|---|
| V32 | 1% | if on LPR |
| M46 | 2% | if on LPR |
| | 12% | If (on IND) and (year of infection < July 2000) |
| | 4% | If (on IND) and (year of infection ≥ July 2000) |
| I47 | 1% | If on LPR |
| I50V | 1% | If on DAR |
| I54 | 2% | If on LPR |
| | 1% | If on DAR |
| L76 | 2% | If on LPR |
| | 1% | If on DAR |
| V82 | 12% | If (on IND) and (year of infection < July 2000) |
| | 4% | If (on IND) and (year of infection ≥ July 2000) |
| | 2% | If on LPR |
| I84 | 12% | If (on IND) and (year of infection < July 2000) |
| | 4% | If (on IND) and (year of infection ≥ July 2000) |
| | 1% | If on DAR |

#### 4.3.5.4 Other classes

| Resistance mutation | Probability of arising | Conditions |
|---|---|---|
| II primary mutation | 80% | If on RAL |
| | 3% | If on DOL |
| II secondary mutation | 3% | If on DOL |

**Comment:** Values of the 'new mutation factor' parameter have been chosen in conjunction with the translation of presence of mutations into reduced drug activity to provide estimates of accumulation of specific classes of resistance mutation broadly consistent with those observed in clinical practice(121, 122). They reflect a greater propensity for some mutations to arise than others. This probability relates to the ability of the virus to replicate without the mutations (e.g. probably very low in the presence of 3TC for virus without M184) as well as the replicative capacity of virus with the mutations in the presence of treatment. Over time as more data accumulate, it may be possible improve these estimates of rates of accumulation of specific mutations.

### 4.3.6 Determination of acquisition of new resistance mutations between t-1 and t: Example calculation

Example

The probability of acquiring a new resistance mutation for a person who has been on their current treatment regimen for between 3-6 months, where their viral load at *t-1* and *t* were 4.5 and 4.7 respectively, who has consistently had an 'effective adherence' of over 0.8 and who has 2.75 active drugs in their current regimen, is:

| | |
|---|---|
| 0.01 | ['new mutation factor' as seen in table 0] |
| x (4.5 + 4.7)/2 | [mean of log viral load at *t-1* and *t*] |

(capped at 1 if this value is > 1).

The resulting number is the probability used in Stage 3 (section 4.3.5) to decide whether there is a chance of any resistance mutations developing in the next 3 month period.

### 4.3.7 Determination of level of activity for each drug

In order to determine the level of activity of each drug it is necessary to determine the level of resistance a certain mutation confers to each drug. The level of resistance to a certain drug, for example ZDV (indicated as r_ZDV) can take the value 0, 0.25, 0.5, 0.75 or 1.

r_ZDV = 0 if there is no resistance to ZDV at time *t*
= 0.25, 0.5 or 0.75 if there is partial resistance to ZDV at time *t*
= 1 if there is complete resistance to ZDV at time *t*

The table below indicate the level of resistance specific mutation confer to specific drugs.

#### 4.3.7.1 NRTI - 3TC

| r_3TC | Mutations | Other conditions |
|---|---|---|
| 0.75 | M184 | - |
| 0.25 | K65 | |
| 0.25 | Q151 | |

The effect of TAMS is the same regardless of presence of r_3TC. This interaction is factored in earlier, at the level of reduced TAM accumulation when on 3TC.

#### 4.3.7.2 NRTI - ZDV

| r_ZDV | Mutations | Other conditions |
|---|---|---|
| 0.5 | 1 ≤ # of TAMS < 3 | Not on 3TC |
| 0.75 | 3 ≤ # of TAMS < 5 | Not on 3TC |
| 1 | # of TAMS ≥ 5 | Not on 3TC |
| 0.25 | 1 ≤ # of TAMS < 3, M184 | On 3TC |
| 0.5 | 3 ≤ # of TAMS < 5, M184 | On 3TC |
| 0.75 | # of TAMS ≥ 5, M184 | On 3TC |
| 0.5 | 1 ≤ # of TAMS < 3 | On 3TC and M184 mutation not present |
| 0.75 | 3 ≤ # of TAMS < 5 | On 3TC and M184 mutation not present |

| | | |
|---|---|---|
| 0.75 | # of TAMS ≥ 5 | On 3TC and M184 mutation not present |
| 0.75 | Q151 | - |

4.3.7.3 NRTI - Tenofovir

| r_TEN | Mutations | Other conditions |
|---|---|---|
| 0.5 | 2 ≤ # of TAMS ≤ 3 | (Not on 3TC<br>or (on 3TC and M184 mutation not present))<br>and K65 mutation not present |
| 0.75 | # of TAMS ≥ 4 | (Not on 3TC<br>or (on 3TC and M184 mutation not present))<br>and K65 mutation not present |
| 0.5 | # of TAMS ≥ 2, M184 | On 3TC and K65 mutation not present |
| 0.75 | K65 | - |

4.3.7.4 NRTI - DDI

| r_DDI | Mutations | Other conditions |
|---|---|---|
| 0.75 | At least 1 mutation of (L74, K65) | - |
| 0.5 | # of TAMS ≥ 3 | - |
| 0.75 | Q151 | - |

4.3.7.5 NNRTIs – Efavirenz

| r_EFA | Mutations | Other conditions |
|---|---|---|
| 1 | nn | - |

4.3.7.6 NNRTIs – Rilpivirine

| r_RPV | Mutations | Other conditions |
|---|---|---|
| 1 | nn | - |

4.3.7.7 PI - Indinavir

Distinguish by pre- and post- boosted.

| r_IND | Mutations | Other conditions |
|---|---|---|
| 0.75 | At least 1 mutation of (M46, V82, I84) | Year of infection < July 2003 |
| 1 | M46, V82, I84 | Year of infection < July 2003 |
| 0.5 | 1 or 2 mutations of (M46, V82, I84) | Year of infection ≥ July 2003 |
| 0.75 | M46, V82, I84 | Year of infection ≥ July 2003 |

#### 4.3.7.8 PI – Lopinavir

| r_LPR | Mutations | Other conditions |
|---|---|---|
| 0.25 | 1 mutation of (V32, L76, V82) | - |
| 0.5 | 2 mutations of (V32, L76, V82) | - |
| 0.75 | 3 mutations of (V32, L76, V82) or I47 | - |
| Max(r_lpr,0.25) | 2 or 3 mutations of (M46, V82, I84, L90) | |
| Max(r_lpr, 0.5) | M46, V82, I84, L90 | - |
| 1 | V32, I47, L76, V82 | |

#### 4.3.7.9 PI – Darunavir

| r_DAR | Mutations | Other conditions |
|---|---|---|
| 0.25 | 2 mutations of (V32,I47, I50V, I54, L76, I84) | - |
| 0.5 | 3 mutations of (V32,I47, I50V, I54, L76, I84) | - |
| 0.75 | At least 4 mutations of (v32,I47, I50V, I54, L76, I84) | - |

#### 4.3.7.10 II - Raltegravir

| r_RAL | Mutations | Other conditions |
|---|---|---|
| 1 | Both primary and secondary II mutations | - |
| 0.75 | Only II primary mutation | - |
| 0.25 | Only II secondary mutation | - |

#### 4.3.7.11 II – Dolutegravir

| r_DOL | | Other conditions |
|---|---|---|
| 1 | Both primary and secondary II mutations | - |
| 0.75 | Only II primary mutation | - |
| 0.25 | Only II secondary mutation | - |

**Comment:** These rules approximately follow the interpretation systems for conversion of mutations present on genotypic resistance test into a predicted level of drug activity (or, equivalently, of resistance). Currently interpretation systems differ in their prediction of activity for some drugs. Over time as more data accumulate and interpretation systems converge it may be possible to refine these rules.

### 4.3.8 Number of active drugs in the regimen

#### 4.3.8.1 Calculation of the number of active drugs in the regimen

The activity level of a drug is given by

activity = 1 - level of resistance to the drug.

The number of active drugs in the regimen at time *t* is the sum of the activity level of each drug in the current regimen. Darunavir and lopinavir are assumed to have double potency compared to the other drugs, while efavirenz, rilpivirine and dolutegravir 50% additional potency.

#### *4.3.8.2* Total number of drugs, *nod*

The total number of drugs the patient is on at time *t*, is simply the sum of the number of antiretroviral drugs they are taking at that time.

In terms of ritonavir-boosted protease inhibitors, we do not count the ritonavir as a separate drug in this case.

### 4.3.9 Loss of resistance mutations

The loss of mutations considered here refers to the loss in resistance mutations from majority virus (and not a complete loss) after stopping a regimen and starting another non-cross-resistant regimen. This section considers only the case of a person who has already started ART, and is not about persistence of transmitted mutations (see section 4.2.2).

#### 4.3.9.1 NRTIs

| Mutation lost | Probability of losing mutation | Conditions |
|---|---|---|
| M184 | 80% | If (M184 mutation is present in majority virus)<br>and (time since stopping 3TC ≥ 3 months, or no previous use of 3TC) |
| K65 | 60% | If (K65 mutation is present in majority virus)<br>and (time since stopping DDI ≥ 3 months, or no previous use of DDI)<br>and (time since stopping TDF ≥ 3 months, or no previous use of TDF) |
| L74 | 60% | If (L74 mutation is present in majority virus)<br>and (time since stopping DDI ≥ 3 months, or no previous use of DDI) |
| Q151 | 60% | If (Q151 mutation is present in majority virus)<br>and (time since stopping DDI ≥ 3 months, or no previous use of DDI)<br>and (time since stopping ZDV ≥ 3 months, or no previous use of ZDV) |
| TAMS* | 40% | If (# of TAMS ≥ 1)<br>and (time since stopping ZDV ≥ 3 months, or no previous use of ZDV)<br>and (time since stopping DDI ≥ 3 months, or no previous use of DDI)<br>and (time since stopping TDF ≥ 3 months, or no previous use of TDF) |

When some loses TAMS mutations, the number of TAMS reverts back to the number of TAMS present at the time of infection.

#### 4.3.9.2 NNRTIs

| Mutation lost | Probability of losing mutation | Conditions |
|---|---|---|
| nn | 20% | If (RTnn mutation is present in majority virus)<br>and (time since stopping EFA ≥ 3 months, or no previous use of EFA)<br>and (time since stopping RPV ≥ 3 months, or no previous use of RPV) |

#### 4.3.9.3 PIs

| Mutation lost | Probability of losing mutation | Conditions |
|---|---|---|
| V32 | 20% | If (V32 mutation is present in majority virus)<br>and (time since stopping LPR ≥ 3 months, or no previous use of LPR) |
| M46 | 20% | If (M46 mutation is present in majority virus)<br>and (time since stopping IND ≥ 3 months, or no previous use of IND) |
| I47 | 20% | If (I47 mutation is present in majority virus)<br>and (time since stopping LPR ≥ 3 months, or no previous use of LPR) |
| I50V | 20% | If (I50V mutation is present in majority virus)<br>and (time since stopping DAR ≥ 3 months, or no previous use of DAR) |
| I54 | 20% | If (I54 mutation is present in majority virus)<br>and (time since stopping DAR ≥ 3 months, or no previous use of DAR) |
| L76 | 20% | If (L76 mutation is present in majority virus)<br>and (time since stopping DAR ≥ 3 months, or no previous use of DAR) |
| V82 | 20% | If (V82 mutation is present in majority virus)<br>and (time since stopping IND ≥ 3 months, or no previous use of IND)<br>and (time since stopping LPR ≥ 3 months, or no previous use of LPR) |
| I84 | 20% | If (I84 mutation is present in majority virus) |

| | | |
|---|---|---|
| | | and (time since stopping IND ≥ 3 months, or no previous use of IND)<br>and (time since stopping DAR ≥ 3 months, or no previous use of DAR) |

4.3.9.4 Other classes

| Mutation lost | Probability of losing mutation | Conditions |
|---|---|---|
| II primary mutation | 20% | If (II primary mutation is present in majority virus)<br>and (time since stopping RAL ≥ 3 months, or no previous use of RAL) |
| | 20% | If (II primary mutation is present in majority virus)<br>and (time since stopping DOL ≥ 3 months, or no previous use of DOL) |
| II secondary mutation | 20% | If (II secondary mutation is present in majority virus)<br>and (time since stopping RAL ≥ 3 months, or no previous use of RAL) |
| | 20% | If (II secondary mutation is present in majority virus)<br>and (time since stopping DOL ≥ 3 months, or no previous use of DOL) |

**Comment:** This is based on evidence from studies in people interrupting ART(124-129).

### 4.3.10 "Regaining" mutations (in majority virus) after restarting ART

Mutations previously present are regained when one of the corresponding drugs is restarted.

### 4.3.11 Arising of new resistance to NNRTI as a result of ART interruption

If a person interrupt treatment while on efavirenz or rilpivirine and did not have resistance to NNRTI there is a 5% chance that it acquires resistance to NNRTI due to the long half life of this class of drugs.

## 4.4 Model fits which are relevant to resistance

### 4.4.1 Risk of resistance mutations (and virologic failure) after start of ART (patients starting with PI/r or NNRTI regimen) Observed data from reference(115).

% with at least one resistance mutation (and virologic failure).

| Years from start of HAART | Observed | Model |
|---|---|---|
| 1 | 4% | 12% |
| 2 | 8% | 16% |
| 3 | 10% | 19% |
| 4 | 11% | 21% |
| 5 | 12% | 23% |
| 6 | 14% | 25% |
| 7 | 16% | 26% |
| 8 | 17% | 27% |

### 4.4.2 Risk of resistance mutations after start of ART *Observed data from reference(122).

% with at least one resistance mutation

| | Years from start of HAART | Observed | Model |
|---|---|---|---|
| M184V mutation | 2 | 6% | 9% |
| (in those starting with 3TC) | 4 | 13% | 14% |
| | 6 | 18% | 17% |
| TAM | 2 | 4% | 8% |
| (in those starting with ZDV or d4T) | 4 | 9% | 11% |
| | 6 | 13% | 13% |
| PI mutation | 2 | 3% | 4% |
| (in those starting with boosted PI regimen) | 4 | 7% | 6% |
| | - | - | - |
| NNRTI mutation | 2 | 8% | 15% |
| (in those starting with NNRTI regimen) | 4 | 14% | 20% |
| | 6 | 21% | 23% |

* Observed data are likely to be underestimates as resistance testing is not always performed at virologic failure.

### 4.4.3 % with at least one resistance mutation for all three main classes (and virologic failure) Observed data from reference(122).

| Years from start of HAART | Observed | Model |
|---|---|---|
| 2 | 1.0% | **0.3%** |
| 4 | 2.7% | **1.6%** |
| 6 | 4.1% | **3.7%** |

### 4.4.4 Risk of death after triple class resistance Observed data from reference(130).

% dead by 3 years (for people with triple class resistance up to July 2004)

| Observed | Model |
|---|---|
| 12% | **21%** |

# 5 Other details

## 5.1 Diagnosis of HIV

Individuals will only be diagnosed post-1984 (which is when HIV testing starts in the model). It is likely there was an increase in testing in the early 2000 with introduction of opt-out testing in GUM clinics(22). We therefore parameterized testing as a fixed rate of testing between 1984 and 1985 ($\alpha_t$ sampled from a 0.03 x exp (Normal(0,0.25)), another fixed rate from 1986 to 2005 ($\alpha_t$ sampled from a 0.28 x exp (Normal(0,0.25)) and a linear change in underlying testing rate from 2006 up to the end of 2021 ($\beta_t$), sampled from a Normal (0.005,0.003)). See Supplementary Figure 5. Afterwards it is assumed to remain fixed at the level achieved.

It is assumed a certain proportion of the population (*rate_*noreached, sampled from a distribution 0.25 x exp (Normal(0,0.25))) cannot be tested unless experiences AIDS and a certain proportion (*rate_hardreached*, sampled from 0.25 x exp (Normal(0,0.25)) ) experience a rate of testing four times lower.
Over time these proportions are reduced, as in each 3 months test a proportion equal to rate_test/50 switches from experience a rate of testing four times lower to non testing unless symptomatic and the same proportion from experiencing a rate of testing four times lower to experiencing the baseline rate of testing.

In addition the probability of testing is further modified if:

- No condomless sex partner in the past year: divided by a factor (*lt*), sampled from a Normal(3.5,1) left truncated at 0.1
- If the subject has at least one condomless sex partner in the past year: multiplied by a factor (*ht*) sampled from Normal (3,1) left truncated at 0.1

This rate of testing applies to people who have not tested in the last year or who had at least one condomless sex short term partner at time *t.*

The probability of someone testing for HIV in any 3 month period depends also on their situation:

| Situation | Probability of HIV testing (per 3 month period) |
|---|---|
| Has an AIDS defining condition | 0.9 |
| Has had TB in the last 3 months, but not an AIDS defining condition | 0.8 |
| Has had a CDC B symptom in the last 3 months, but not TB nor an AIDS defining condition | 0.3 |

If a person living with HIV is tested the person is considered diagnosed with HIV and cannot get tested again. Only once diagnosed with HIV a person can be linked to care (see section 5.1).

## 5.2 Management of people living with HIV in care

### 5.2.1 Linkage to care

Once a person living with HIV gets diagnosed with HIV (see section 5.1) all those presenting with AIDS disease, CDC B symptoms or AIDS are assumed to be linked and staged (i.e. it is assessed whether the person is eligible to be initiated on ART and receive PCP) while for the remaining it is assumed a small proportion (0.01) is lost from care. However they will be able to come back in care later in time (see section 5.3.2.2).

### 5.2.2 ART initiation

#### 5.2.2.1 First line regimen

The calendar year at which highly active ART (HAART) is assumed to become available is mid-1996. It is assumed that before that only ZDV was available and that time at the time HAART becomes available all people in ART are switched to the regimen ZDV+3TC+IND.

From 2000 it is assumed a resistance test is performed before ART initiation and if they have mutations that confer resistance to the standard first line regimen they will be initiated on a different regimen.

| Calendar year | First line regimen | Regimen in case of resistance |
|---|---|---|
| Before highly active ART | ZDV | NA |
| HAART-2000 | ZDV+3TC+IND | NA |
| 2000-2007 | ZDV+3TC+EFA | If resistance to EFA: ZDV+3TC+LPR |
| 2007-2014 | TDF+3TC+EFA | If resistance to EFA: TDF+3TC+LPR |
| | | If resistance to 3TC: ZDV+DDI+EFA |
| | | If resistance to EFA and 3TC: ZDV+DDI+LPR |
| 2014- | TEN+3TC+DOL | If resistance to DOL: TEN+3TC+DAR |
| | | If resistance to TEN: ZDV+3TC+DOL |
| | | If resistance to DOL and TEN: ZDV+3TC+DAR |

#### 5.2.2.2 Eligibility criteria to be initiated on ART

The eligibility criteria to be initiated on ART varied over time and are described in the table below. In people with AIDS the probability of being initiated on ART is 0.9, while for the others unless it is indicated in the table below is 0.7.

| Calendar year of eligibility criteria introduction | Eligibility criteria to be initiated on ART | Notes on probability of being initiated on ART for people in care per 3 month |
|---|---|---|
| 1988- | AIDS disease | |
| 1990- | measured CD4 (in the last 6m) <200 or AIDS | |
| 2006- | measured CD4 (in the last 6m) <350 or AIDS | |
| 2012- | All people diagnosed | 0.075 if measured CD4 above 350 |
| Mid-2015 | All people diagnosed | 0.35 if measured CD4 above 350 |
| Mid-218 | All people diagnosed | 0.7 if measured CD4 above 350 (as for people with lower CD4 and no AIDS) |

For the new eligibility criteria to be fully implemented it is assumed it takes:
- 15 months (20% of the population per 3 months are switched to the new eligibility criteria) in 2006
- 30 months (10% of the population per 3 months are switched to the new eligibility criteria) in 2012
- 9 months (40% of the population per 3 months are switched to the new eligibility criteria) in mid-2015

### 5.2.3 Care for people on ART

#### 5.2.3.1 ART monitoring

People living with HIV (whether ART naïve or ART experienced) when they are in care are assumed to be seen every 3 months and at each visit it is assumed their CD4 and VL is measured. The VL is measured to assess whether they achieve and maintain virological suppression. People who have been on a line treatment for 9 consecutive months or who have initiated treatment more than 6 months before and who have interrupted treatment but the clinic is not aware and have a measured VL above 500 copies/mL are considered to have failed the line of treatment they are currently on.

#### 5.2.3.2 Choice of 2nd line regimen

People who have failed the first line regimen can be switched to a new regimen and this depends on calendar year and whether they have developed toxicities to any drugs and whether they have developed resistance to a certain drug, with the only exception of 3TC. This seemed reasonable given in the UK clinician have access to the resistance history, .

| Calendar year | Toxicities | Regimen |
|---|---|---|
| 2000- | | DDI+3TC+LPR |
| | DDI | ZDV+3TC+LPR |
| | LPR | DDI+3TC+EFA |
| | DDI and LPR | ZDV+3TC+EFA |
| 2003- | | TDF+3TC+LPR |
| | TDF | ZDV+3TC+LPR |
| | LPR | TDF+3TC+EFA |
| | TDF and EFA | ZDV+3TC+EFA |
| 2009- | LPR | TDF+3TC+RAL |
| | TDF, LPR and RAL | ZDV+3TC+RAL |
| 2014- | | TDF+3TC+DAR |
| | TDF | ZDV+3TC+DAR |

#### 5.2.3.3 Choice of 3rd line regimen

People who have failed the first and second line regimen can be switched to a new regimen and this depends on calendar year and whether they have developed toxicities to any drugs.

| Calendar year | Toxicities | Regimen |
|---|---|---|
| 2007- | | TDF+3TC+RAL+DAR |
| | TDF | ZDV+3TC+RAL+DAR |
| | TDF and ZDV | DDI+3TC+RAL+DAR |
| 2011- | | DAR+RPV |
| | TDF and ZDV | DAR+RPV |
| 2014- | | TDF+3TC+DAR+DOL |
| | TDF | ZDV+3TC+DAR+DOL |
| | TDF and ZDV | DAR+DOL |

## 5.3 Interruption of ART

### 5.3.1 Treatment interruption

#### 5.3.1.1 Probability of Interruption (due to choice)

The probability of interruption in a given 3 month period whilst a patient is on ART, depends on the ‘underlying tendency to adhere’ (for more details on how adherence is modelled, see section 3.1.1), presence of any current toxicities (see section 5.4), presence of lactic acidosis and age. The probability of interruptions by underlying tendency to adhere and presence of any current toxicities is calculated as follows:

| ‘Underlying tendency to adhere’ | Presence of any current toxicities | Probability of Interruption (prointer) |
|---|---|---|
| ≥ 0.8 | Yes | 0.02 |
| | No | 0.01 |
| 0.5 – 0.79 | Yes | 0.03 |
| | No | 0.015 |
| < 0.5 | Yes | 0.4 |
| | No | 0.02 |

Therefore, the probability of interruption is greater in patients with low ‘underlying tendency to adhere’ and in the presence of current toxicities.

The effect of age on the chance of interrupting ART is calculated using the following formulas:

- $prointer = prointer * [(40 - age(t)) * 0.11]$ if age(t)<40
- $prointer = prointer * [(age(t) - 40) * 0.11]$ if age(t)>40

where prointer is the probability of interrupting treatment based on the tendency to adhere and the presence of current toxicities.

This probability is halved after a 1 year consecutive on treatment, as there is evidence that rate of discontinuation decrease over time. This is divided by 3 from 2014 onwards.
Finally, it is assumed people with lactic acidosis have a probability of 1 of interrupting treatment

So a person aged 20 with low underlying tendency to adherence and current toxicities but not lactic acidosis will have a probability of interrupting treatment of 0.264, calculated as

$$prointer = 0.12 * [(40 - 20) * 0.11] = 0.264$$

#### 5.3.1.2 Treatment interruption coinciding with patient’s missing clinic visits

There is also a chance that the treatment interruption coincides with patients interrupting visits to the clinic. The probability of this event happening depends on the ‘underlying tendency to adhere’:

- 50% if ‘underlying tendency to adhere’ ≥ 0.8
- 100% if 0.5 ≤ ‘underlying tendency to adhere’ < 0.8
- 100% if ‘underlying tendency to adhere’ < 0.5

#### 5.3.1.3 Clinician aware of treatment interruption

When an individual interrupts treatment, the clinician will not always be aware of the fact that they have stopped treatment. We have incorporated a 50% probability of the clinician not being aware of an incidence of treatment interruption in any 3-month period whilst the individual has stopped treatment. This only affects whether it counts as virologic failure when the patient’s viral load is not suppressed; i.e. if a patient has interrupted ART and the clinic is not aware, then when the viral load shows a rebound, this will be interpreted as virologic failure.

**Comment:** See references(123, 131-135).

### 5.3.2 Loss to follow-up / Clinic visits

#### 5.3.2.1 Loss to follow-up whilst the patient is OFF ART

'Loss to follow-up', implies a period of missing clinic visits, not necessarily indefinite cessation of clinic visits. The probability of being lost to follow-up at any visit whilst the patient is off ART depends on the 'underlying tendency to adhere':

- 5% if 'underlying tendency to adhere' ≥ 0.8
- 7.5% if 0.5 ≤ 'underlying tendency to adhere' < 0.8
- 10% if 'underlying tendency to adhere' < 0.5

#### 5.3.2.2 Returning to clinic after loss to follow-up

The probability of returning to clinic visits after loss to follow-up depends on the ‘underlying tendency to adhere’ and whether the person has AIDS.

If the individual developed AIDS in the previous 3-month period, they have an 80% probability of returning to clinic visits. Otherwise, the probability of someone returning to clinic after loss to follow-up is:

- 20% if ‘underlying tendency to adhere’ ≥ 0.8
- 10% if 0.5 ≤ ‘underlying tendency to adhere’ < 0.8
- 6.7% if ‘underlying tendency to adhere’ < 0.5

### 5.3.3 Viral load and CD4 count changes during ART interruption

#### 5.3.3.1 Viral load changes

When an individual interrupts ART, their viral load returns to the maximum viral load (achieved before the time of interruption) in 3 months and adopts natural history changes thereafter (i.e. those in ART-naïve patients, see section 2.2 for these rates).

#### 5.3.3.2 CD4 count changes

When an individual interrupts ART, their rate of CD4 count decline depends on how long they have interrupted for and also their current viral load. This is summarised in the table below:

| Period of ART interruption | Current viral load (log copies/ml) | Distribution of change in CD4 count (cells/mm$^3$) |
|---|---|---|
| 0-3 months | VL ≥ 5 | Normal (-200,10) |
| | 4.5 ≤ VL < 5 | Normal (-160,10) |
| | VL < 4.5 | Normal (-120,10) |
| 3-6 months | VL ≥ 5 | Normal (-100,10) |
| | 4.5 ≤ VL < 5 | Normal (-90,10) |
| | VL < 4.5 | Normal (-80,10) |
| 6-9 months | VL ≥ 5 | Normal (-80,10) |
| | 4.5 ≤ VL < 5 | Normal (-70,10) |
| | VL < 4.5 | Normal (-60,10) |

If an individual interrupts treatment for more than 3 months, but their CD4 count is over 300 cells/mm$^3$ above their CD4 nadir, then their change in CD4 count over this period of time is the same as for someone who has interrupted for 0-3 months (i.e. first 3 rows in the table).

Once the change in CD4 count has been taken into account, the resulting CD4 count for an individual cannot be less than their CD4 nadir. Their resulting CD4 count will therefore be limited to their CD4 nadir at any time point.

If the individual’s CD4 count reaches the CD4 nadir, then the rate of CD4 count decline adopts natural history changes thereafter (see section 2.3.1 for these rates). However, once the individual

interrupts treatment for at least 9 months, their subsequent rate of CD4 count decline also adopts natural history changes, even if the nadir had not been reached by then.

**Comment:** These values are broadly based on evidence from a number of analyses of the effects of ART interruption (131, 132, 136-144).

### 5.3.4 Re-initiation of ART after interruption

The probability of re-starting therapy after treatment interruption (in a person who is visiting the clinic) is 0.8, unless the patient had a CDC B symptom or an AIDS disease in the last 3 months, in these cases it is 1.

## 5.4 Toxicities

Toxicities including gastrointestinal symptoms, rash, hepatoxicity, CNS toxicity, lipodystrophy, peripheral neuropathy and nephrolithiasis can occur with certain probability when the individual is on certain specific drugs.

### 5.4.1 List of toxicities modelled

| Abbreviation used | Toxicity modelled |
|---|---|
| NAU | Nausea |
| DIA | Diarrhoea |
| RAS | Rash |
| CNS | CNS toxicity |
| LIP | Lipodystrophy |
| PEN | Peripheral neuropathy |
| HEP | Hepatoxicity |
| NEPH | Nephrolithiasis |
| OTX | Generic toxicity, with unknown profile |
| ANE | Anaemia |
| HEAD | Headache |
| PANC | Pancreatitis |
| LAC | Lactic acidosis |
| TOX | Any toxicity |

All these variables are binary, i.e. if the individual develops a certain toxicity in a given 3-month period, it takes the value 1, otherwise 0. The variable TOX takes the value 1 if more than one of any of the other toxicities are present and 0 otherwise.

The variable OTX is included so that it is possible to stop new drugs with unknown adverse event profiles.

### 5.4.2 Incidence of new current toxicity

All individuals do not have any toxicities at the start of simulation (i.e. point of infection). Summarised below is the probability of developing a new current toxicity in any given 3-month period.

| | NAU | DIA | RAS | CNS | LIP | PEN | NEPH | OTX | ANE | HEAD | PANC | LAC |
|---|---|---|---|---|---|---|---|---|---|---|---|---|
| ZDV | 0.03 | | | | 0.015 | | | 0.03 | 0.03 | 0.1 | | 0.002 |
| 3TC | | | | | | | | 0.03 | | | | |
| DDI | 0.03 | 0.05 | | | | 0.01 | | 0.03 | | | 0.001 | 0.002 |
| TEN | | | | | | | 0.0035 | 0.03 | | | | |
| EFA | | | 0.03[a] | 0.1[b] | | | | 0.03 | | | | |
| RPV | | | 0.01[a] | | | | | | | | | |
| IND | | | | | | | | 0.03 | | | | |
| LPR | 0.03 | 0.02 | | | | | | 0.03 | | | | |
| DAR | 0.01 | 0.01 | | | | | | 0.03 | | | | |
| DOL | | | | 0.05[b] | | | | | | | | |

a. If on the drug now, but was not on 6 months ago
b. Have been on current regimen for less than 1 year

The probabilities shown above are independent for each toxicity

There is an increased risk of some toxicities in the first year of starting ART. The raised risk is 5 fold for NAU, 2.5-fold for DIA and 1.5 for PEN, OTX, ANE and HEAD.

### 5.4.3 Continuation of existing toxicity

Summarised below is the probability of retaining an existing toxicity in any given 3-month period. There are some exceptions (see comment below).

| | NAU | DIA | CNS | ANE | HEAD |
|---|---|---|---|---|---|
| ZDV | 0.5 | | | 0.2 | 0.4 |
| 3TC | | | | | |
| DDI | 0.5 | 0.5 | | | |
| TEN | | | | | |
| EFA | | | 0.8* | | |
| IND | | | | | |
| LPR | 0.5 | 0.5 | | | |
| DAR | 0.5 | 0.5 | | | |
| DOL | | | 0.4* | | |

*this applies if patient has been on current regimen for less than 1 year. 0.9 if they have been on current regimen for more than or equal to 1 year.

The interpretation of the above table is as follows. For example, there is a 50% chance of someone with NAU at time *t* still having nausea at *t+1* if (on ZDV or DDI or DAR).

There is an increased risk of some toxicities continuing in the first year of starting ART. The raised risk is 5 fold for NAU, 2.5 fold for DIA, 1.5-fold for ANE and HEAD.

**Comment:** Once an individual has lipodystrophy, they are modeled to always have lipodystrophy, even if they stop ZDV. Once an individual has peripheral neuropathy, they are modeled to have peripheral neuropathy for as long as stay on DDI (i.e. they will not have it anymore as soon as they stop DDI). Once a person has nephrolithiasis the toxicity remains as long as they remain on TDF, if they are not anymore on TDF had they had nephrolithiasis in the last 3 months period they have 90% chance of still having it.

The following toxicities are only given a probability of acquiring it (but not retaining it): rash, hepatoxicity, generic toxicity, pancreatitis, lactic acidosis. The reason for this is because we have modeled it such that once an individual gets one or more of these toxicities, the drug that is causing this toxicity is switched if the toxicity is sufficiently severe and that it will otherwise be resolved.

These probabilities are based broadly on evidence from trials and cohort studies, although there are no common definitions for some conditions which complicate this. Data on toxicities based on drug labels were also used. Further refinement will be possible as more data accumulate.

## 5.5 Regimen switching

The switching of regimens is dealt by first considering whether the patient should be switching and then considering which antiretrovirals to switch to. The probability of switching depends on whether the switching is being considered because the patient has virologically failed their regimen or because they have developed toxicities to their current regimen.

The choice of drug to switch to is dealt separately in this documentation for 'pre-2003' and 'post-2003'. There is no particular reason other than the two being dealt separately also in the model. After 2003, switching was assumed to be fully based on resistance testing.

### 5.5.1 Probability of switching – due to virological failure of regimen

#### 5.5.1.1 Initiation of second line HAART (for people who failed first line and still on first line)

Individuals have a 60% chance of initiating (or switching to) the second line regimen in every 3 month period, after year 2000; 90% from 2014 onwards.

#### 5.5.1.2 Initiation of third line HAART (for people who failed second line and still on second line)

Similar to above, individuals have a 60% chance of initiating (or switching to) the third line regimen in every 3 month period, but only after year 2007; 90% from 2014 onwards.

### 5.5.2 Probability of switching – due to toxicity

If toxicity is present then individual drugs may be switched due to toxicity.

The table below summarises the probability of switching due to toxicity in any given 3-month period. Any blanks in the table imply that the particular drug will not be stopped by the corresponding toxicity.

| | NAU | DIA | RAS | CNS | LIP | PEN | HEP | NEPH | OTX | ANE | HEAD | PANC | LAC |
|---|---|---|---|---|---|---|---|---|---|---|---|---|---|
| ZDV | 0.08 | | | | 0.01 | | | | | 0.05 | 0.02 | | 1 |
| 3TC | | | | | | | | | | | | | |
| DDI | 0.05 | 0.05 | | | | 0.1 | | | | | | 1 | 1 |
| TEN | | | | | | | | 0.8 | 0.1 | | | | 1 |
| EFA | | | 0.05 | 0.02 | | | | | | | | | |
| RPV | | | 0.05 | 0.02 | | | | | | | | | |
| IND | 0.25 | | | | | | | 0.4 | | | | | |
| LPR | 0.05 | 0.05 | | | | | | | | | | | |
| DAR | | | | | | | | | | | | | |
| RAL | | | | | | | | | | | | | |
| DOL | | | | 0.02 | | | | | | | | | |

### 5.5.3 Drugs people living with HIV are switched to due to toxicity

The table below indicates to which drug people are switched to due to toxicity, given the availability of drugs in a certain calendar year and the fact that they are not resistant to it. This is due to the fact that in the UK the clinician has access to resistance history. If the probabilities indicated in the table in section 5.5.2 arise.
Note that the numbers inside the table indicate the order of drugs they can be switched to as long they have not already experience toxicity to those drugs and they are not already receiving them. As an example if they fail ZDV, the calendar year is after 2003, they are not receiving TDF and they have no toxicity to TDF they will be switched to TDF. If one of the conditions above is not fulfilled they will be switched to DDI and if not possible to 3TC.

| | | Drug at time t, to which they are swicthed to (year of availability) | | | | | | | | | | |
|---|---|---|---|---|---|---|---|---|---|---|---|---|
| | | ZDV | 3TC (1994) | DDI (1992) | TDF (2003) | EFA (2000) | RPV (2011) | IND | LPR (2000) | DAR (2008) | RAL (2008) | DOL (2014) |
| Drug | ZDV | | (2) | (3) | (1) | | | | | | | |
| at | 3TC | | | | | | | | | | | |
| time | DDI | (3) | (2) | | (1) | | | | | | | |
| t-1 | TEN | (1) | | | | | | | | | | |
| | EFA | | | | | | | | (2) | (1)* | | |
| | RPV | | | | | | | | (2) | (1)* | | |
| | IND | | | | | (4) | (2) | | (5) | (3)** | | (1) |
| | LPR | | | | | (4) | (2) | | | (3) | | (1) |
| | DAR | | | | | | | | | | | |
| | DOL | | | | | | | | | (1)** | (2) | |

*From 2014; **from 2009

For 3TC, DAR it is assumed that either there are no toxicities to these drugs (3TC) or people are not switched to different drugs because of toxicities.

1. Phillips AN, Cambiano V, Nakagawa F, Brown AE, Lampe F, Rodger A, et al. Increased HIV incidence in men who have sex with men despite high levels of ART-induced viral suppression: analysis of an extensively documented epidemic. PLoS One. 2013;8(2):e55312.
2. Rodger AJ, McCabe L, Phillips AN, Lampe FC, Burns F, Ward D, et al. Free HIV self-test for identification and linkage to care of previously undetected HIV infection in men who have sex with men in England and Wales (SELPHI): an open-label, internet-based, randomised controlled trial. Lancet HIV. 2022;9(12):e838-e47.
3. Miners A, Phillips A, Kreif N, Rodger A, Speakman A, Fisher M, et al. Health-related quality-of-life of people with HIV in the era of combination antiretroviral treatment: a cross-sectional comparison with the general population. Lancet HIV. 2014(1):e32-e40.
4. Salomon JA, Vos T, Hogan DR, Gagnon M, Naghavi M, Mokdad A, et al. Common values in assessing health outcomes from disease and injury: disability weights measurement study for the Global Burden of Disease Study 2010. Lancet. 2012;380(9859):2129-43.
5. Lima VD, Zhu J, Card KG, Lachowsky NJ, Chowell-Puente G, Wu Z, et al. Can the combination of TasP and PrEP eliminate HIV among MSM in British Columbia, Canada? Epidemics. 2021;35:100461.
6. Curtis LAB, A.;. Unit Costs of Health & Social Care 2020. . University of Kent; 2020. Contract No.: 978-1-911353-12-6.
7. Beaumont MA, Zhang W, Balding DJ. Approximate Bayesian computation in population genetics. Genetics. 2002;162(4):11.
8. Rodger AJ, Cambiano V, Bruun T, Vernazza P, Collins S, van Lunzen J, et al. Sexual Activity Without Condoms and Risk of HIV Transmission in Serodifferent Couples When the HIV-Positive Partner Is Using Suppressive Antiretroviral Therapy. JAMA. 2016;316(2):171-81.
9. Bansi L, Sabin C, Delpech V, Hill T, Fisher M, Walsh J, et al. Trends over calendar time in antiretroviral treatment success and failure in HIV clinic populations. HIV Med. 2010;11(7):432-8.
10. Nakagawa F, Lodwick RK, Smith CJ, Smith R, Cambiano V, Lundgren JD, et al. Projected life expectancy of people with HIV according to timing of diagnosis. AIDS. 2012;26(3):335-43.
11. Phillips AN, Sabin C, Pillay D, Lundgren JD. HIV in the UK 1980-2006: reconstruction using a model of HIV infection and the effect of antiretroviral therapy. HIV Med. 2007;8(8):536-46.
12. Phillips AN, Cambiano V, Miners A, Lampe FC, Rodger A, Nakagawa F, et al. Potential impact on HIV incidence of higher HIV testing rates and earlier antiretroviral therapy initiation in MSM. AIDS. 2015;29(14):1855-62.
13. Mercer CH, Fenton KA, Copas AJ, Wellings K, Erens B, McManus S, et al. Increasing prevalence of male homosexual partnerships and practices in Britain 1990-2000: evidence from national probability surveys. AIDS. 2004;18(10):1453-8.
14. Sonnenberg P, Clifton S, Beddows S, Field N, Soldan K, Tanton C, et al. Prevalence, risk factors, and uptake of interventions for sexually transmitted infections in Britain: findings from the National Surveys of Sexual Attitudes and Lifestyles (Natsal). Lancet. 2013;382(9907):1795-806.
15. statistics; Ofn. Figure 2: Age structure of the UK population, mid-2016 and mid-2041 2017 [Available from: https://www.ons.gov.uk/peoplepopulationandcommunity/populationandmigration/populationprojections/bulletins/nationalpopulationprojections/2016basedstatisticalbulletin.
16. Mercer CH, Tanton C, Prah P, Erens B, Sonnenberg P, Clifton S, et al. Changes in sexual attitudes and lifestyles in Britain through the life course and over time: findings from the National Surveys of Sexual Attitudes and Lifestyles (Natsal). Lancet. 2013;382(9907):1781-94.
17. Carne CA, Weller IV, Johnson AM, Loveday C, Pearce F, Hawkins A, et al. Prevalence of antibodies to human immunodeficiency virus, gonorrhoea rates, and changed sexual behaviour in homosexual men in London. Lancet. 1987;1(8534):656-8.
18. Dodds JP, Mercey DE, Parry JV, Johnson AM. Increasing risk behaviour and high levels of undiagnosed HIV infection in a community sample of homosexual men. Sex Transm Infect. 2004;80(3):236-40.

19. Williamson LM, Dodds JP, Mercey DE, Johnson AM, Hart GJ. Increases in HIV-related sexual risk behavior among community samples of gay men in London and Glasgow: how do they compare? J Acquir Immune Defic Syndr. 2006;42(2):238-41.
20. Elford J, Bolding G, Sherr L. High-risk sexual behaviour increases among London gay men between 1998 and 2001: what is the role of HIV optimism? AIDS. 2002;16(11):1537-44.
21. Elford J, Bolding G, Davis M, Sherr L, Hart G. Trends in sexual behaviour among London homosexual men 1998-2003: implications for HIV prevention and sexual health promotion. Sex Transm Infect. 2004;80(6):451-4.
22. Dougan S, Elford J, Chadborn TR, Brown AE, Roy K, Murphy G, et al. Does the recent increase in HIV diagnoses among men who have sex with men in the UK reflect a rise in HIV incidence or increased uptake of HIV testing? Sex Transm Infect. 2007;83(2):120-5; discussion 5.
23. Statistics OfN. Sexual orientation, UK: 2020 2022 [Available from: https://www.ons.gov.uk/peoplepopulationandcommunity/culturalidentity/sexuality/bulletins/sexualidentityuk/2020.
24. Baggaley RF, White RG, Boily MC. HIV transmission risk through anal intercourse: systematic review, meta-analysis and implications for HIV prevention. Int J Epidemiol. 2010;39(4):1048-63.
25. Vittinghoff E, Douglas J, Judson F, McKirnan D, MacQueen K, Buchbinder SP. Per-contact risk of human immunodeficiency virus transmission between male sexual partners. Am J Epidemiol. 1999;150(3):306-11.
26. Hollingsworth TD, Anderson RM, Fraser C. HIV-1 transmission, by stage of infection. J Infect Dis. 2008;198(5):687-93.
27. Jin F, Jansson J, Law M, Prestage GP, Zablotska I, Imrie JC, et al. Per-contact probability of HIV transmission in homosexual men in Sydney in the era of HAART. AIDS. 2010;24(6):907-13.
28. Quinn TC, Wawer MJ, Sewankambo N, Serwadda D, Li C, Wabwire-Mangen F, et al. Viral load and heterosexual transmission of human immunodeficiency virus type 1. Rakai Project Study Group. N Engl J Med. 2000;342(13):921-9.
29. Presanis AM, Gill ON, Chadborn TR, Hill C, Hope V, Logan L, et al. Insights into the rise in HIV infections, 2001 to 2008: a Bayesian synthesis of prevalence evidence. AIDS. 2010;24(18):2849-58.
30. Williamson LM, Dodds JP, Mercey DE, Hart GJ, Johnson AM. Sexual risk behaviour and knowledge of HIV status among community samples of gay men in the UK. AIDS. 2008;22(9):1063-70.
31. Research S. Final Reports.
32. Marks G, Crepaz N, Senterfitt JW, Janssen RS. Meta-analysis of high-risk sexual behavior in persons aware and unaware they are infected with HIV in the United States: implications for HIV prevention programs. J Acquir Immune Defic Syndr. 2005;39(4):446-53.
33. Pharris A, editor 90-90-90 Goals in Europe: In action. 17th European AIDS Conference; 2019; Basel, Switzerland.
34. O'Halloran C SS, Nash S, Brown A, Croxford S, Connor N, Sullivan AK, Delpech V, Gill ON. HIV in the United Kingdom: Towards Zero 2030. 2019 report. London: Public Health England; 2019.
35. Nash S, Desai, S.,Croxford, S.,Guerra,L.,Lowndes,C.,Connor,N.,Gill, O.N.,. Progress towards ending the HIV epidemic in the United Kingdom 2018 report. . London: Public Health England; 2018.
36. Brown AE KP, Chau C, Khawam J, Gill ON, Delpech VC. Towards elimination of HIV transmission, AIDS and HIV-related deaths in the UK – 2017 report. London: Public Health England; 2017.
37. Kirwan PD CC, Brown AE, Gill ON, Delpech VC and contributors. . HIV in the UK - 2016 report. London: Public Health England; 2016.
38. Skingsley A YZ, Kirwan P, Croxford S, Chau C, Conti S, Presanis A, Nardone A, Were J, Ogaz D, Furegato M, Hibbert M, Aghaizu A, Murphy G, Tosswill J, Hughes G, Anderson J, Gill ON, Delpech VC and contributors. HIV in the UK – Situation Report 2015: data to end 2014. London: Public Health England; 2015.

39. Public Health England. Trends in HIV testing, new diagnoses and people receiving HIV-related care in the United Kingdom: data to the end of December 2019. London: Health Protection Report; 2020 03/11/2020. Contract No.: 20.
40. Lester J, Martin V, Shah A, Chau C, Mackay N, Newbigging-Lister A, et al. HIV testing, PrEP, new HIV diagnoses, and care outcomes for people accessing HIV services: 2022 report. The annual official statistics data release (data to end of December 2021). London: UK Health Security Agency; October 2022.
41. Gill ON, Ogaz, D., Blomquist, P., Khawam, J., Kirwan, P., Brizzi, F., Brown, A., Mohammed, H., Birrell, P., DeAngelis, D., Hughes, G., Delpech, V.,, editor The fall in HIV infections in MSM in England during 2012 through 2016: When did it begin and what caused it? IUSTI; 2018; Dublin, Ireland.
42. Gill ON. OFFICIAL: CONFIDENTIAL - Monitoring PrEP effectiveness in England - Calibration of Synthesis model to PHE data. In: Cambiano V, editor. 2019.
43. Health Protection Agency. Time to test for HIV: Expanding HIV testing in healthcare and community services in England. 2011.
44. Health Protection Agency. HIV in the United Kingdom: 2012 Report. Colindale, London; 2012.
45. Martin V SA, Mackay N, Lester J, Newbigging-Lister A, Connor N, Brown AE, Sullivan AK, Delpech VC, and contributors. HIV testing, new HIV diagnoses, outcomes and quality of care for people accessing HIV services: 2021 report. The annual official statistics data release (data to end of December 2020). London: UK Health Security Agency; 2021 December 2021.
46. Public Health Wales Communicable Disease Surveillance Centre. HIV and STI trends in Wales Surveillance Report. Cardiff: Public Health Wales; 2012.
47. Kuller LH, Ockene JK, Meilahn E, Wentworth DN, Svendsen KH, Neaton JD. Cigarette smoking and mortality. MRFIT Research Group. Prev Med. 1991;20(5):638-54.
48. Cohen MS. Sexually transmitted diseases enhance HIV transmission: no longer a hypothesis. Lancet. 1998;351 Suppl 3:5-7.
49. McCormack S, Dunn DT, Desai M, Dolling DI, Gafos M, Gilson R, et al. Pre-exposure prophylaxis to prevent the acquisition of HIV-1 infection (PROUD): effectiveness results from the pilot phase of a pragmatic open-label randomised trial. Lancet. 2016;387(10013):53-60.
50. Cohen SE, Vittinghoff E, Bacon O, Doblecki-Lewis S, Postle BS, Feaster DJ, et al. High interest in preexposure prophylaxis among men who have sex with men at risk for HIV infection: baseline data from the US PrEP demonstration project. J Acquir Immune Defic Syndr. 2015;68(4):439-48.
51. Grant RM, Anderson PL, McMahan V, Liu A, Amico KR, Mehrotra M, et al. Uptake of pre-exposure prophylaxis, sexual practices, and HIV incidence in men and transgender women who have sex with men: a cohort study. Lancet Infect Dis. 2014;14(9):820-9.
52. Centers for Disease Control and Prevention. Pree-exposure prophylaxis for the prevention of HIV infection in the United States - 2014. A clinical practice guideline 2014 [updated 2014.
53. British HIV Association, British Association of Sexual Health and HIV, British Infection Society. UK National Guidelines for HIV Testing 20082008. Available from: http://www.bhiva.org/documents/guidelines/testing/glineshivtest08.pdf.
54. Koot M, Keet IP, Vos AH, de Goede RE, Roos MT, Coutinho RA, et al. Prognostic value of HIV-1 syncytium-inducing phenotype for rate of CD4+ cell depletion and progression to AIDS. Ann Intern Med. 1993;118(9):681-8.
55. Pantazis N, Touloumi G. Bivariate modelling of longitudinal measurements of two human immunodeficiency type 1 disease progression markers in the presence of informative drop-outs. Journal of the Royal Statistical Society. 2005;Series C-Applied Statistics(54):405-23.
56. Touloumi G, Pantazis N, Babiker AG, Walker SA, Katsarou O, Karafoulidou A, et al. Differences in HIV RNA levels before the initiation of antiretroviral therapy among 1864 individuals with known HIV-1 seroconversion dates. AIDS. 2004;18(12):1697-705.
57. Sabin CA, Devereux H, Phillips AN, Hill A, Janossy G, Lee CA, et al. Course of viral load throughout HIV-1 infection. J Acquir Immune Defic Syndr. 2000;23(2):172-7.

58. Hubert JB, Burgard M, Dussaix E, Tamalet C, Deveau C, Le Chenadec J, et al. Natural history of serum HIV-1 RNA levels in 330 patients with a known date of infection. The SEROCO Study Group. AIDS. 2000;14(2):123-31.
59. O'Brien TR, Rosenberg PS, Yellin F, Goedert JJ. Longitudinal HIV-1 RNA levels in a cohort of homosexual men. J Acquir Immune Defic Syndr Hum Retrovirol. 1998;18(2):155-61.
60. Henrard DR, Phillips JF, Muenz LR, Blattner WA, Wiesner D, Eyster ME, et al. Natural history of HIV-1 cell-free viremia. JAMA. 1995;274(7):554-8.
61. Lyles RH, Munoz A, Yamashita TE, Bazmi H, Detels R, Rinaldo CR, et al. Natural history of human immunodeficiency virus type 1 viremia after seroconversion and proximal to AIDS in a large cohort of homosexual men. Multicenter AIDS Cohort Study. J Infect Dis. 2000;181(3):872-80.
62. Mellors JW, Munoz A, Giorgi JV, Margolick JB, Tassoni CJ, Gupta P, et al. Plasma viral load and CD4+ lymphocytes as prognostic markers of HIV-1 infection. Ann Intern Med. 1997;126(12):946-54.
63. Touloumi G, Karafoulidou A, Gialeraki A, Katsarou O, Milona I, Kapsimali V, et al. Determinants of progression of HIV infection in a Greek hemophilia cohort followed for up to 16 years after seroconversion. J Acquir Immune Defic Syndr Hum Retrovirol. 1998;19(1):89-97.
64. Phillips AN, Elford J, Sabin C, Bofill M, Janossy G, Lee CA. Immunodeficiency and the risk of death in HIV infection. JAMA. 1992;268(19):2662-6.
65. Lundgren JD, Pedersen C, Clumeck N, Gatell JM, Johnson AM, Ledergerber B, et al. Survival differences in European patients with AIDS, 1979-89. The AIDS in Europe Study Group. BMJ. 1994;308(6936):1068-73.
66. Wright TL, Hollander H, Pu X, Held MJ, Lipson P, Quan S, et al. Hepatitis C in HIV-infected patients with and without AIDS: prevalence and relationship to patient survival. Hepatology. 1994;20(5):1152-5.
67. Eyster ME, Diamondstone LS, Lien JM, Ehmann WC, Quan S, Goedert JJ. Natural history of hepatitis C virus infection in multitransfused hemophiliacs: effect of coinfection with human immunodeficiency virus. The Multicenter Hemophilia Cohort Study. J Acquir Immune Defic Syndr. 1993;6(6):602-10.
68. Soriano V, Garcia-Samaniego J, Valencia E, Rodriguez-Rosado R, Munoz F, Gonzalez-Lahoz J. Impact of chronic liver disease due to hepatitis viruses as cause of hospital admission and death in HIV-infected drug users. Eur J Epidemiol. 1999;15(1):1-4.
69. Graham CS, Baden LR, Yu E, Mrus JM, Carnie J, Heeren T, et al. Influence of human immunodeficiency virus infection on the course of hepatitis C virus infection: a meta-analysis. Clin Infect Dis. 2001;33(4):562-9.
70. Touloumi G, Hatzakis A, Rosenberg PS, O'Brien TR, Goedert JJ. Effects of age at seroconversion and baseline HIV RNA level on the loss of CD4+ cells among persons with hemophilia. Multicenter Hemophilia Cohort Study. AIDS. 1998;12(13):1691-7.
71. Ledergerber B, Lundgren JD, Walker AS, Sabin C, Justice A, Reiss P, et al. Predictors of trend in CD4-positive T-cell count and mortality among HIV-1-infected individuals with virological failure to all three antiretroviral-drug classes. Lancet. 2004;364(9428):51-62.
72. Phillips AN, Lee CA, Elford J, Webster A, Janossy G, Timms A, et al. More rapid progression to AIDS in older HIV-infected people: the role of CD4+ T-cell counts. J Acquir Immune Defic Syndr. 1991;4(10):970-5.
73. Phillips A, Pezzotti P, Collaboration C. Short-term risk of AIDS according to current CD4 cell count and viral load in antiretroviral drug-naive individuals and those treated in the monotherapy era. AIDS. 2004;18(1):51-8.
74. Mallolas J, Zamora L, Gatell JM, Miro JM, Vernet E, Valls ME, et al. Primary prophylaxis for Pneumocystis carinii pneumonia: a randomized trial comparing cotrimoxazole, aerosolized pentamidine and dapsone plus pyrimethamine. AIDS. 1993;7(1):59-64.
75. Deeks SG, Phillips AN. HIV infection, antiretroviral treatment, ageing, and non-AIDS related morbidity. BMJ. 2009;338:a3172.

76. Phillips AN, Neaton J, Lundgren JD. The role of HIV in serious diseases other than AIDS. AIDS. 2008;22(18):2409-18.
77. Frisch M, Biggar RJ, Engels EA, Goedert JJ, Group AI-CMRS. Association of cancer with AIDS-related immunosuppression in adults. JAMA. 2001;285(13):1736-45.
78. Herida M, Mary-Krause M, Kaphan R, Cadranel J, Poizot-Martin I, Rabaud C, et al. Incidence of non-AIDS-defining cancers before and during the highly active antiretroviral therapy era in a cohort of human immunodeficiency virus-infected patients. J Clin Oncol. 2003;21(18):3447-53.
79. Maggi P, Quirino T, Ricci E, De Socio GV, Gadaleta A, Ingrassia F, et al. Cardiovascular risk assessment in antiretroviral-naive HIV patients. AIDS Patient Care STDS. 2009;23(10):809-13.
80. Francisci D, Giannini S, Baldelli F, Leone M, Belfiori B, Guglielmini G, et al. HIV type 1 infection, and not short-term HAART, induces endothelial dysfunction. AIDS. 2009;23(5):589-96.
81. Lewden C, Chene G, Morlat P, Raffi F, Dupon M, Dellamonica P, et al. HIV-infected adults with a CD4 cell count greater than 500 cells/mm3 on long-term combination antiretroviral therapy reach same mortality rates as the general population. J Acquir Immune Defic Syndr. 2007;46(1):72-7.
82. Study Group on Death Rates at High CDCiANP, Lodwick RK, Sabin CA, Porter K, Ledergerber B, van Sighem A, et al. Death rates in HIV-positive antiretroviral-naive patients with CD4 count greater than 350 cells per microL in Europe and North America: a pooled cohort observational study. Lancet. 2010;376(9738):340-5.
83. Time from HIV-1 seroconversion to AIDS and death before widespread use of highly-active antiretroviral therapy: a collaborative re-analysis. Collaborative Group on AIDS Incubation and HIV Survival including the CASCADE EU Concerted Action. Concerted Action on SeroConversion to AIDS and Death in Europe. Lancet. 2000;355(9210):1131-7.
84. Lodi S, Phillips A, Touloumi G, Geskus R, Meyer L, Thiebaut R, et al. Time from human immunodeficiency virus seroconversion to reaching CD4+ cell count thresholds <200, <350, and <500 Cells/mm(3): assessment of need following changes in treatment guidelines. Clin Infect Dis. 2011;53(8):817-25.
85. Dorrucci M, Rezza G, Porter K, Phillips A, Concerted Action on Seroconversion to A, Death in Europe C. Temporal trends in postseroconversion CD4 cell count and HIV load: the Concerted Action on Seroconversion to AIDS and Death in Europe Collaboration, 1985-2002. J Infect Dis. 2007;195(4):525-34.
86. Carrieri MP, Raffi F, Lewden C, Sobel A, Michelet C, Cailleton V, et al. Impact of early versus late adherence to highly active antiretroviral therapy on immuno-virological response: a 3-year follow-up study. Antivir Ther. 2003;8(6):585-94.
87. Paterson DL, Swindells S, Mohr J, Brester M, Vergis EN, Squier C, et al. Adherence to protease inhibitor therapy and outcomes in patients with HIV infection. Ann Intern Med. 2000;133(1):21-30.
88. Nieuwkerk P, Gisolf E, Sprangers M, Danner S, Prometheus Study G. Adherence over 48 weeks in an antiretroviral clinical trial: variable within patients, affected by toxicities and independently predictive of virological response. Antivir Ther. 2001;6(2):97-103.
89. Walsh JC, Mandalia S, Gazzard BG. Responses to a 1 month self-report on adherence to antiretroviral therapy are consistent with electronic data and virological treatment outcome. AIDS. 2002;16(2):269-77.
90. Bangsberg DR, Porco TC, Kagay C, Charlebois ED, Deeks SG, Guzman D, et al. Modeling the HIV protease inhibitor adherence-resistance curve by use of empirically derived estimates. J Infect Dis. 2004;190(1):162-5.
91. Bangsberg DR, Moss AR, Deeks SG. Paradoxes of adherence and drug resistance to HIV antiretroviral therapy. J Antimicrob Chemother. 2004;53(5):696-9.
92. Bannister WP, Kirk O, Gatell JM, Knysz B, Viard JP, Mens H, et al. Regional changes over time in initial virologic response rates to combination antiretroviral therapy across Europe. J Acquir Immune Defic Syndr. 2006;42(2):229-37.

93. Lampe FC, Gatell JM, Staszewski S, Johnson MA, Pradier C, Gill MJ, et al. Changes over time in risk of initial virological failure of combination antiretroviral therapy: a multicohort analysis, 1996 to 2002. Arch Intern Med. 2006;166(5):521-8.
94. de Bethune MP. Non-nucleoside reverse transcriptase inhibitors (NNRTIs), their discovery, development, and use in the treatment of HIV-1 infection: a review of the last 20 years (1989-2009). Antiviral Res. 2010;85(1):75-90.
95. Roge BT, Barfod TS, Kirk O, Katzenstein TL, Obel N, Nielsen H, et al. Resistance profiles and adherence at primary virological failure in three different highly active antiretroviral therapy regimens: analysis of failure rates in a randomized study. HIV Med. 2004;5(5):344-51.
96. King M, Brun S, Tschampa J, Moseley J, Kempf D. Exploring the effects of adherence on resistance: use of local linear regression to reveal relationships between adherence and resistance in antiretroviral-naive patients treated with lopinavir/ritonavir or nelfinavir. Antivir Ther 2003;8(3):U103-U4.
97. Beck EJ, Mandalia S, Sangha R, Sharott P, Youle M, Baily G, et al. The cost-effectiveness of early access to HIV services and starting cART in the UK 1996-2008. PLoS One. 2011;6(12):e27830.
98. De Meyer S, Lathouwers E, Dierynck I, De Paepe E, Van Baelen B, Vangeneugden T, et al. Characterization of virologic failure patients on darunavir/ritonavir in treatment-experienced patients. AIDS. 2009;23(14):1829-40.
99. King JR, Wynn H, Brundage R, Acosta EP. Pharmacokinetic enhancement of protease inhibitor therapy. Clin Pharmacokinet. 2004;43(5):291-310.
100. Buss N, Snell P, Bock J, Hsu A, Jorga K. Saquinavir and ritonavir pharmacokinetics following combined ritonavir and saquinavir (soft gelatin capsules) administration. Br J Clin Pharmacol. 2001;52(3):255-64.
101. BHIVA Writing Committee on behalf of the BHIVA Executive Committee. British HIV Association (BHIVA) guidelines for the treatment of HIV-infected adults with antiretroviral therapy. HIV Medicine. 2001;2(4).
102. BHIVA Writing Committee on behalf of the BHIVA Executive Committee. British HIV Association (BHIVA) guidelines for the treatment of HIV-infected adults with antiretroviral therapy. HIV Medicine. 2003;4(Suppl 1).
103. Gallant JE, Staszewski S, Pozniak AL, DeJesus E, Suleiman JM, Miller MD, et al. Efficacy and safety of tenofovir DF vs stavudine in combination therapy in antiretroviral-naive patients: a 3-year randomized trial. JAMA. 2004;292(2):191-201.
104. Phillips AN, Staszewski S, Weber R, Kirk O, Francioli P, Miller V, et al. HIV viral load response to antiretroviral therapy according to the baseline CD4 cell count and viral load. JAMA. 2001;286(20):2560-7.
105. Staszewski S, Miller V, Sabin C, Carlebach A, Berger AM, Weidmann E, et al. Virological response to protease inhibitor therapy in an HIV clinic cohort. AIDS. 1999;13(3):367-73.
106. Staszewski S, Miller V, Sabin C, Schlecht C, Gute P, Stamm S, et al. Determinants of sustainable CD4 lymphocyte count increases in response to antiretroviral therapy. AIDS. 1999;13(8):951-6.
107. van Leth F, Phanuphak P, Ruxrungtham K, Baraldi E, Miller S, Gazzard B, et al. Comparison of first-line antiretroviral therapy with regimens including nevirapine, efavirenz, or both drugs, plus stavudine and lamivudine: a randomised open-label trial, the 2NN Study. Lancet. 2004;363(9417):1253-63.
108. Ledergerber B, Egger M, Opravil M, Telenti A, Hirschel B, Battegay M, et al. Clinical progression and virological failure on highly active antiretroviral therapy in HIV-1 patients: a prospective cohort study. Swiss HIV Cohort Study. Lancet. 1999;353(9156):863-8.
109. Mocroft A, Phillips AN, Gatell J, Ledergerber B, Fisher M, Clumeck N, et al. Normalisation of CD4 counts in patients with HIV-1 infection and maximum virological suppression who are taking combination antiretroviral therapy: an observational cohort study. Lancet. 2007;370(9585):407-13.

110. Gras L, Kesselring AM, Griffin JT, van Sighem AI, Fraser C, Ghani AC, et al. CD4 cell counts of 800 cells/mm3 or greater after 7 years of highly active antiretroviral therapy are feasible in most patients starting with 350 cells/mm3 or greater. J Acquir Immune Defic Syndr. 2007;45(2):183-92.
111. Eron JJ, Benoit SL, Jemsek J, MacArthur RD, Santana J, Quinn JB, et al. Treatment with lamivudine, zidovudine, or both in HIV-positive patients with 200 to 500 CD4+ cells per cubic millimeter. North American HIV Working Party. N Engl J Med. 1995;333(25):1662-9.
112. Hammer SM, Katzenstein DA, Hughes MD, Gundacker H, Schooley RT, Haubrich RH, et al. A trial comparing nucleoside monotherapy with combination therapy in HIV-infected adults with CD4 cell counts from 200 to 500 per cubic millimeter. AIDS Clinical Trials Group Study 175 Study Team. N Engl J Med. 1996;335(15):1081-90.
113. Egger M, May M, Chene G, Phillips AN, Ledergerber B, Dabis F, et al. Prognosis of HIV-1-infected patients starting highly active antiretroviral therapy: a collaborative analysis of prospective studies. Lancet. 2002;360(9327):119-29.
114. Sterne JA, Hernan MA, Ledergerber B, Tilling K, Weber R, Sendi P, et al. Long-term effectiveness of potent antiretroviral therapy in preventing AIDS and death: a prospective cohort study. Lancet. 2005;366(9483):378-84.
115. Resistance UKCGoHD, Group UCS. Long-term probability of detecting drug-resistant HIV in treatment-naive patients initiating combination antiretroviral therapy. Clin Infect Dis. 2010;50(9):1275-85.
116. Smith CJ, Phillips AN, Hill T, Fisher M, Gazzard B, Porter K, et al. The rate of viral rebound after attainment of an HIV load <50 copies/mL according to specific antiretroviral drugs in use: results from a multicenter cohort study. J Infect Dis. 2005;192(8):1387-97.
117. Pursuing Later Treatment Options IIPTftCoOHIVERE, Lodwick R, Costagliola D, Reiss P, Torti C, Teira R, et al. Triple-class virologic failure in HIV-infected patients undergoing antiretroviral therapy for up to 10 years. Arch Intern Med. 2010;170(5):410-9.
118. Johnson VA, Calvez V, Günthard HF, Paredes R, Pillay D, Shafer RW, et al. Update of the Drug Resistance Mutations in HIV-1. Top Antivir Med. 2011;19(4):156-64.
119. Corvasce S, Violin M, Romano L, Razzolini F, Vicenti I, Galli A, et al. Evidence of differential selection of HIV-1 variants carrying drug-resistant mutations in seroconverters. Antivir Ther. 2006;11(3):329-34.
120. Turner D, Brenner B, Routy JP, Moisi D, Rosberger Z, Roger M, et al. Diminished representation of HIV-1 variants containing select drug resistance-conferring mutations in primary HIV-1 infection. J Acquir Immune Defic Syndr. 2004;37(5):1627-31.
121. Harrigan PR, Hogg RS, Dong WW, Yip B, Wynhoven B, Woodward J, et al. Predictors of HIV drug-resistance mutations in a large antiretroviral-naive cohort initiating triple antiretroviral therapy. J Infect Dis. 2005;191(3):339-47.
122. Phillips AN, Dunn D, Sabin C, Pozniak A, Matthias R, Geretti AM, et al. Long term probability of detection of HIV-1 drug resistance after starting antiretroviral therapy in routine clinical practice. AIDS. 2005;19(5):487-94.
123. DeGruttola V, Dix L, D'Aquila R, Holder D, Phillips A, Ait-Khaled M, et al. The relation between baseline HIV drug resistance and response to antiretroviral therapy: re-analysis of retrospective and prospective studies using a standardized data analysis plan. Antivir Ther. 2000;5(1):41-8.
124. Devereux HL, Emery VC, Johnson MA, Loveday C. Replicative fitness in vivo of HIV-1 variants with multiple drug resistance-associated mutations. J Med Virol. 2001;65(2):218-24.
125. Birk M, Svedhem V, Sonnerborg A. Kinetics of HIV-1 RNA and resistance-associated mutations after cessation of antiretroviral combination therapy. AIDS. 2001;15(11):1359-68.
126. Deeks SG, Grant RM, Wrin T, Paxinos EE, Liegler T, Hoh R, et al. Persistence of drug-resistant HIV-1 after a structured treatment interruption and its impact on treatment response. AIDS. 2003;17(3):361-70.

127. Walter H, Low P, Harrer T, Schmitt M, Schwingel E, Tschochner M, et al. No evidence for persistence of multidrug-resistant viral strains after a 7-month treatment interruption in an HIV-1-infected individual. J Acquir Immune Defic Syndr. 2002;31(2):137-46.
128. Hance AJ, Lemiale V, Izopet J, Lecossier D, Joly V, Massip P, et al. Changes in human immunodeficiency virus type 1 populations after treatment interruption in patients failing antiretroviral therapy. J Virol. 2001;75(14):6410-7.
129. Tarwater PM, Parish M, Gallant JE. Prolonged treatment interruption after immunologic response to highly active antiretroviral therapy. Clin Infect Dis. 2003;37(11):1541-8.
130. Grover D, Copas A, Green H, Edwards SG, Dunn DT, Sabin C, et al. What is the risk of mortality following diagnosis of multidrug-resistant HIV-1? J Antimicrob Chemother. 2008;61(3):705-13.
131. Li X, Margolick JB, Conover CS, Badri S, Riddler SA, Witt MD, et al. Interruption and discontinuation of highly active antiretroviral therapy in the multicenter AIDS cohort study. J Acquir Immune Defic Syndr. 2005;38(3):320-8.
132. d'arminio Monforte A, Cozzi-Lepri A, Phillips A, De Luca A, Murri R, Mussini C, et al. Interruption of highly active antiretroviral therapy in HIV clinical practice: results from the Italian Cohort of Antiretroviral-Naive Patients. J Acquir Immune Defic Syndr. 2005;38(4):407-16.
133. Mocroft A, Youle M, Moore A, Sabin CA, Madge S, Lepri AC, et al. Reasons for modification and discontinuation of antiretrovirals: results from a single treatment centre. AIDS. 2001;15(2):185-94.
134. Concorde: MRC/ANRS randomised double-blind controlled trial of immediate and deferred zidovudine in symptom-free HIV infection. Concorde Coordinating Committee. Lancet. 1994;343(8902):871-81.
135. Cotton P. Use of antiretroviral drugs in HIV disease declines following preliminary results from Concorde trial. JAMA. 1994;271(7):488-9.
136. Skiest DJ, Morrow P, Allen B, McKinsey J, Crosby C, Foster B, et al. It is safe to stop antiretroviral therapy in patients with preantiretroviral CD4 cell counts >250 cells/microL. J Acquir Immune Defic Syndr. 2004;37(3):1351-7.
137. Youle M, Janossy G, Turnbull W, Tilling R, Loveday C, Mocroft A, et al. Changes in CD4 lymphocyte counts after interruption of therapy in patients with viral failure on protease inhibitor-containing regimens. Royal Free Centre for HIV Medicine. AIDS. 2000;14(12):1717-20.
138. Lawrence J, Mayers DL, Hullsiek KH, Collins G, Abrams DI, Reisler RB, et al. Structured treatment interruption in patients with multidrug-resistant human immunodeficiency virus. N Engl J Med. 2003;349(9):837-46.
139. Tebas P, Henry K, Mondy K, Deeks S, Valdez H, Cohen C, et al. Effect of prolonged discontinuation of successful antiretroviral therapy on CD4+ T cell decline in human immunodeficiency virus-infected patients: implications for intermittent therapeutic strategies. J Infect Dis. 2002;186(6):851-4.
140. Fischer M, Hafner R, Schneider C, Trkola A, Joos B, Joller H, et al. HIV RNA in plasma rebounds within days during structured treatment interruptions. AIDS. 2003;17(2):195-9.
141. Boschi A, Tinelli C, Ortolani P, Moscatelli G, Morigi G, Arlotti M. CD4+ cell-count-guided treatment interruptions in chronic HIV-infected patients with good response to highly active antiretroviral therapy. AIDS. 2004;18(18):2381-9.
142. Achenbach CJ, Till M, Palella FJ, Knoll MD, Terp SM, Kalnins AU, et al. Extended antiretroviral treatment interruption in HIV-infected patients with long-term suppression of plasma HIV RNA. HIV Med. 2005;6(1):7-12.
143. Thiebaut R, Pellegrin I, Chene G, Viallard JF, Fleury H, Moreau JF, et al. Immunological markers after long-term treatment interruption in chronically HIV-1 infected patients with CD4 cell count above 400 x 10(6) cells/l. AIDS. 2005;19(1):53-61.

144. Wit FW, Blanckenberg DH, Brinkman K, Prins JM, van der Ende ME, Schneider MM, et al. Safety of long-term interruption of successful antiretroviral therapy: the ATHENA cohort study. AIDS. 2005;19(3):345-8.
145. Mocroft A, Phillips AN, Soriano V, Rockstroh J, Blaxhult A, Katlama C, et al. Reasons for stopping antiretrovirals used in an initial highly active antiretroviral regimen: increased incidence of stopping due to toxicity or patient/physician choice in patients with hepatitis C coinfection. AIDS Res Hum Retroviruses. 2005;21(6):527-36.
